# Immune Response Elicited by Recombinant Adenovirus-Delivered Glycoprotein B and Nucleocapsid Protein UL18 and UL25 of HSV-1 in Mice

**DOI:** 10.3390/ijms252413486

**Published:** 2024-12-16

**Authors:** Haobo Zhang, Qi Li, Yun Liao, Danjing Ma, Fengyuan Zeng, Zhenxiao Zhang, Li Yu, Rong Yue, Xinghang Li, Yuansheng Liao, Dandan Li, Guorun Jang, Heng Zhao, Xin Zhao, Huiwen Zheng, Heng Li, Longding Liu, Ying Zhang

**Affiliations:** 1 Yunnan Key Laboratory of Vaccine Research and Development for Severe Infectious Diseases, Institute of Medical Biology, Chinese Academy of Medicine Sciences & Peking Union Medical College, Kunming 650118, China; s1051187712@163.com (H.Z.); iyoliqi@163.com (Q.L.); liaoyun@imbcams.com.cn (Y.L.); ynyxmdj@student.pumc.edu.cn (D.M.); 12020214294@imbcams.com.cn (F.Z.); zhangzhenxiao@student.pumc.edu.cn (Z.Z.); yuli@imbcams.com.cn (L.Y.); s2020018024@student.pumc.edu.cn (R.Y.); lixinghang@student.pumc.edu.cn (X.L.); lysS2022018021@imb.cams.cn (Y.L.); lidandan@imbcams.com.cn (D.L.); jgr@imbcams.com.cn (G.J.); zhaoheng@imbcams.com.cn (H.Z.); xz479633385@163.com (X.Z.); zhenghuiwen@imbcams.com.cn (H.Z.); liheng@imbcams.com.cn (H.L.); 2 School of Life Sciences, Yunnan University, Kunming 650500, China

**Keywords:** herpes simplex virus type 1, nucleocapsid protein, UL18, UL25, glycoprotein B

## Abstract

Due to the complex pathogenic and immune escape mechanisms of herpes simplex virus type 1 (HSV-1), especially the failure of induced immune responses to block the initial cell-to-cell transmission of the virus from skin cells to neurons, the body struggles to establish effective prevention and control methods, resulting in the failure of currently developed vaccines. Previous studies have highlighted the crucial roles of surface glycoproteins and nucleocapsid proteins in activating the body’s immune defense system against HSV-1 infection. In this study, recombinant adenoviruses were used as vectors to generate adenoviruses carrying the nucleocapsid protein genes UL18 and UL25, as well as the surface glycoprotein gene gB. This approach aimed to mimic the protein expression process that occurs following viral infection of the host and to investigate the immune response characteristics induced by UL18, UL25, and gB proteins. The findings revealed that UL18, UL25, and gB proteins could all trigger the expression of genes associated with innate immune responses; however, the specific genes induced varied in type and level. Furthermore, all three proteins were capable of promoting the proliferation of CD8+ T cells in the lymph nodes. Notably, only UL18 and gB could elicit a Th1 cell immune response. Interestingly, among these proteins, only UL18 could also induce a relatively higher IL-4 level, indicating a Th2 cell immune response. In addition to cellular immunity, all three proteins stimulated the production of specific IgG antibodies. Notably, UL18 induced higher and more sustained levels of specific IgG antibodies in mice. By contrast, only glycoprotein gB induced lower levels of neutralizing antibodies in mice. Moreover, when these mice were challenged with HSV-1, the co-immunization with UL18 and gB provided better protection than gB alone. In conclusion, HSV-1 surface glycoproteins and nucleocapsid proteins exhibit differences in their ability to induce innate and adaptive immunity in the body, suggesting potential avenues for vaccine design by leveraging their complementary advantages.

## 1. Introduction

Herpes simplex virus 1 (HSV-1), a member of the subfamily Alphaherpesvirinae, is an enveloped double-stranded DNA virus [1]. Humans are its only natural hosts, and the infection rate is remarkably high; according to the World Health Organization, about 67% of the global population has been infected with HSV-1 [2]. The most common clinical manifestation of this infection is primary herpetic gingivostomatitis (PHGS) [3]. By binding to specific cell receptors to trigger membrane fusion, viruses enter the initial target cell [4] subsequently spread to infect innervating sensory neurons [5,6], and then are transported retrogradely through axons to the dorsal root ganglia particularly the trigeminal ganglion, establishing a lifelong latency [6,7]. During this latent phase, the virus does not express proteins necessary for replication but instead shows low expression of the latency-associated transcript (LAT) gene, allowing it to coexist with the host in a dormant state for life [8]. Reactivation can occur under certain conditions or due to non-specific triggers such as cold, fever, or a weakened immune response, leading to a return to a lytic state. This reactivation causes the virus to travel back down to infect several dermatomes, such as the skin or mucous membranes, resulting in tissue damage and recurrent outbreaks. In rare cases, reactivated virus particles can reach the brain, causing severe diffuse acute infections known as herpes simplex encephalitis [9,10]. The impact of HSV-1 infection and its recurrences significantly affects patients’ quality of life and mental well-being [8]. Current clinical treatments for HSV primarily consist of antiviral medications such as valacyclovir, famciclovir, acyclovir, penciclovir, trifluridine, phosphonoformate, iododeoxyuridine, vidarabine, and cidofovir [11]. However, these drugs can have toxic side effects [12], and drug-resistant strains of HSV have emerged in immunocompromised patients [13]. Consequently, vaccine development is seen as a promising solution to combat HSV infections.

Over the years, several vaccines against HSV-1 have been developed. Earlier approaches involved inactivating the virus using methods like formalin treatment, ultraviolet irradiation, or heating to create vaccines. However, these inactivated vaccines exhibited low immunogenicity and raised concerns regarding potential oncogenicity, limiting their use in practice [14,15]. Advances in molecular biology have facilitated a deeper understanding of various HSV-1 genes and proteins, identifying new targets for vaccine development. Some vaccines have been designed by deleting genes associated with viral infection, replication, or latency, such as ICP0, ICP4, and ICP27 [16]. While these vaccines can stimulate humoral and cellular immune responses, there is a risk that strains with deleted genes may regain their pathogenicity. Thus, safety concerns surrounding these vaccines necessitate thorough evaluation during development [17]. Subunit vaccines, which express HSV-1 viral proteins in large quantities through prokaryotic or eukaryotic expression systems followed by purification and co-administration with adjuvants, have gained attention for their safety profile. HSV-1 envelope glycoproteins, located on the outermost layer of the virus particles, play crucial roles in mediating viral attachment and entry into host cells. These glycoproteins are also the first point of contact with the host immune system, triggering an immune response against the virus [18]. Consequently, glycoproteins C (gC) and D (gD) are considered promising target proteins for subunit vaccine development [19]. Despite this potential, results from large clinical trials have shown that subunit vaccines primarily targeting gC/gD provide limited protection against HSV [17,20]. Similarly, nucleic acid vaccines also focus on glycoprotein genes like gC and gD but have not demonstrated sufficient efficacy in preventing HSV infection or recurrence in clinical trials [17,21,22]. As a result, researchers are increasingly exploring other viral proteins as candidate antigen targets for future vaccine development [23].

On the basis of the life cycle of HSV-1 and previous research findings, it is understood that after the nucleocapsid is released into the cytoplasm following the fusion of the HSV-1 envelope, a second interaction occurs between the nucleocapsid proteins and the host cell contents. This interaction triggers an immune response against the virus within the cells [24]. However, limited information is available regarding the differences in host immune responses elicited by these two steps. Understanding these differences is crucial for comprehensively grasping the relationship between HSV and host antiviral immunity, which can inform vaccine development efforts.

To compare the immune responses induced by various viral proteins, several methods are available for introducing target proteins into host cells. Among these, the replication-defective human recombinant adenovirus 5 (ΔAd5) is particularly noteworthy. It can deliver target genes into cells to express antigens, thereby activating both innate and adaptive immunity. Although ΔAd5 cannot replicate within cells, it is widely used in vaccine research and development due to its favorable safety profile [25,26].

In this study, we selected an envelope glycoprotein gB because it contains T-cell epitopes [27,28,29] and is reported to have stronger immunogenicity, similar to gD, among the envelope glycoproteins [27,30,31]. We also included two nucleocapsid proteins, UL18 and UL25. UL18 is an important capsid protein and has a numerical advantage in viral particles [32], while UL25 was identified in our previous study as the dominant antigen for antibody production in HSV-infected mice. Our goal was to evaluate the characteristics of the immune response induced by the envelope glycoproteins and nucleocapsid proteins. To induce an effective systemic immune response against HSV [27,33,34,35], mice were intranasally inoculated with recombinant adenoviruses containing the gene for the envelope glycoprotein B (gB) and the nucleocapsid protein genes UL18 or UL25. The focus was on detecting specific T-cell responses and antibodies induced by these three proteins presented via ΔAd5 inoculation in vivo. This approach allows for an analysis of the characteristics and differences in the interactions between the virus and the host immune response in the presence of these proteins.

## 2. Results

### 2.1. Recombinant Adenovirus Expressing UL18, UL25, and gB Protein in 293T Cells

**Figure 1:**
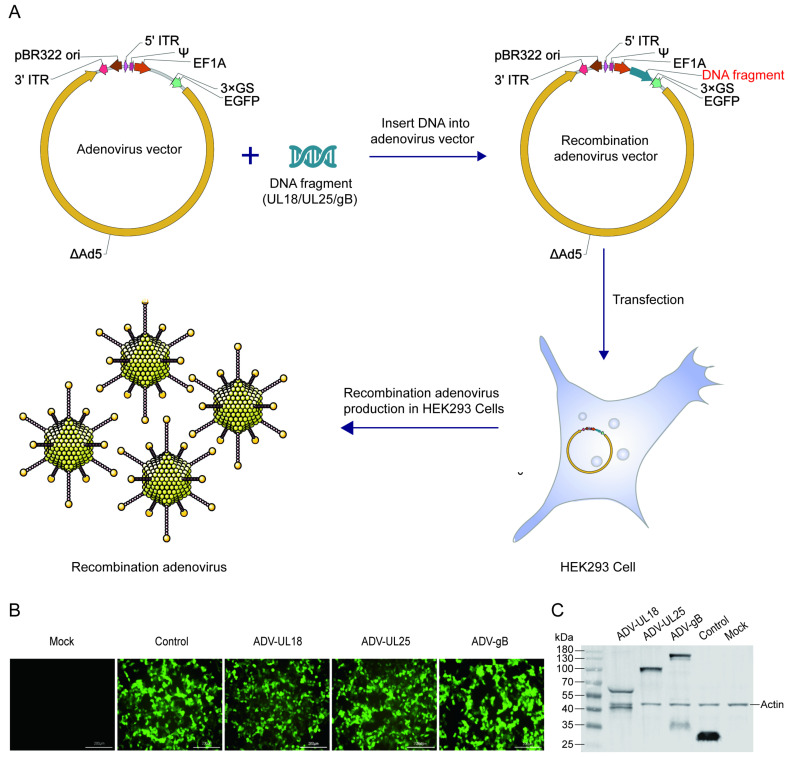
The expression of recombinant adenoviruses ADV-UL18, ADV-UL25, and ADV-gB in vitro. (**A**) Schematic diagram of the construction of recombinant adenoviruses ADV-UL18, ADV-UL25, and ADV-gB. (**B**) Fluorescence detection of protein expressions. (**C**) Western blotting to confirm protein expression using anti-EGFP antibody for protein detection. UL18-EGFP is approximately 62 kDa, UL25-EGFP is approximately 90 kDa, gB-EGFP is approximately 126.5 kDa, and EGFP is approximately 26 kDa. “Mock” represents the blank control (untreated 293T cells), while “Control” represents the adenovirus vector-transduced 293T cells. All samples were collected 24 h after transduction.

Three recombinant adenoviruses were developed using ΔAd5 as the vector, with UL18, UL25, or gB fused with EGFP as reporters. The resulting recombinant adenovirus vectors were transfected into HEK-293 cells, which served as packaging cells, leading to the production of ADV-UL18, ADV-UL25, and ADV-gB (Figure 1A). After amplification, purification, and titer assessment, these recombinant adenoviruses were used for subsequent experimental research. Twenty-four hours after transducing 293T cells with the recombinant adenoviruses, the expression of UL18, UL25, and gB proteins was verified using fluorescence imaging (Figure 1B) and Western blot analysis (Figure 1C).

### 2.2. Expression of UL18, ADV-UL25, and ADV-gB in Mice Intranasally Inoculated with Recombinant ADV-UL18, ADV-UL25, and ADV-gB

**Figure 2:**
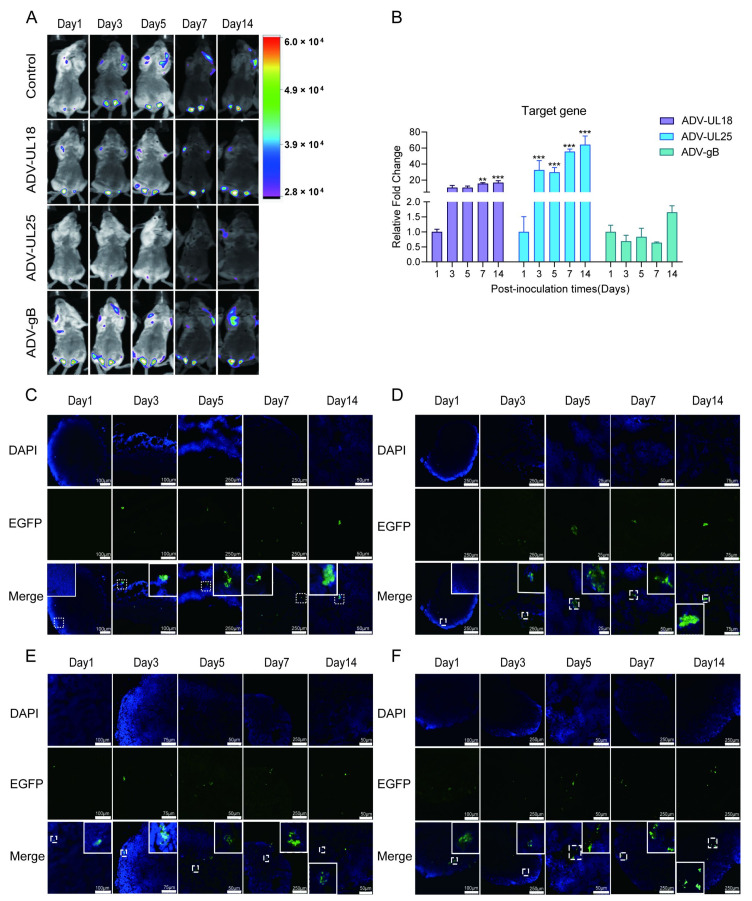
The expression of recombinant adenoviruses ADV-UL18, ADV-UL25, and ADV-gB in vivo. (**A**) In vivo imaging detects protein expression in mice. The mice were intranasally inoculated with recombinant adenovirus. Red in the heat map indicates relatively high expression levels, while purple indicates relatively low expression levels. (**B**) Transcription of UL18, UL25, and UL27 (gB) genes in the lymph nodes of mice inoculated intranasally with recombinant adenovirus. (**C**) Detection of EGFP protein expression in the lymph nodes by immunofluorescence. (**D**) Detection of UL18 protein expression in the lymph nodes by immunofluorescence. (**E**) Detection of UL25 protein expression in the lymph nodes by immunofluorescence. (**F**) Detection of gB protein expression in the lymph nodes by immunofluorescence. The relative Ct (ΔΔCt) method was used to normalize the relative expression levels of mRNA at each time point in each group to the corresponding mRNA expression level on day 1 in each group. The Scheir–Ray–Hare test was performed. Data are presented as the mean ± SD based on data from three independent experiments. ** *p* < 0.01, *** *p* < 0.001.

To determine whether these recombinant adenoviruses can deliver the target gene in vivo and express the target protein, we intranasally inoculated 6-week-old female Balb/c mice with the blank adenoviral vector (control), ADV-UL18, ADV-UL25, and ADV-gB (7 × 10^9^ VP/mouse). In vivo imaging indicated that UL18, UL25, and gB proteins were primarily detected in the lymph nodes, including the cervical, brachial, axillary, and inguinal lymph nodes (Figure 2A). To further validate the expression levels of the target genes in the mouse lymph nodes, we isolated the lymph nodes (superficial cervical, axillary, brachial, and inguinal) and assessed the transcription of the UL18, UL25, and UL27 (gB) genes using qRT-PCR. Our findings revealed a significant increase in the transcription of UL18 and UL25 genes from 3 to 14 days post-inoculation (d.p.i.). (Figure 2B, *p* < 0.05), while the UL27 (gB) gene exhibited a general upward trend without a significant difference compared to the control (Figure 2B, *p* > 0.05). Immunofluorescence results also demonstrated that the expression of UL18 (Figure 2D), UL25 (Figure 2E), and gB (Figure 2F) proteins in the lymph nodes increased with prolonged inoculation time. These results confirm that ADV-UL18, ADV-UL25, and ADV-gB can effectively carry and express the target genes in vivo.

### 2.3. Innate Immune Response Induced by UL18, UL25, and gB Proteins

**Figure 3:**
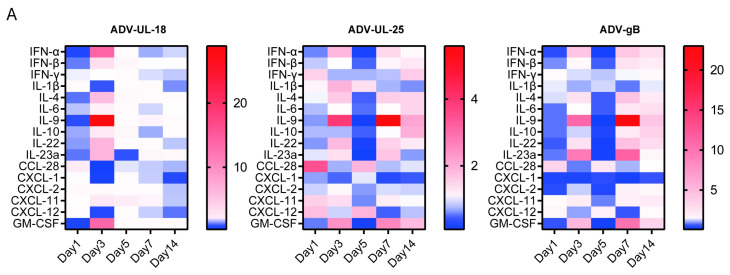

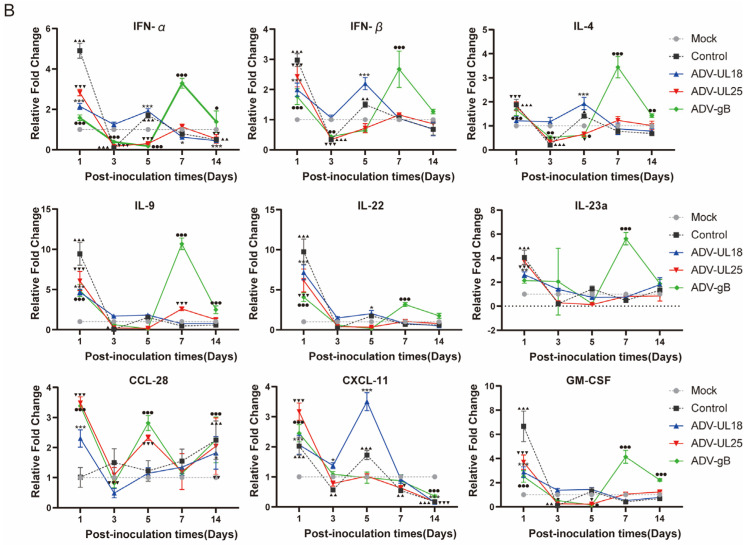

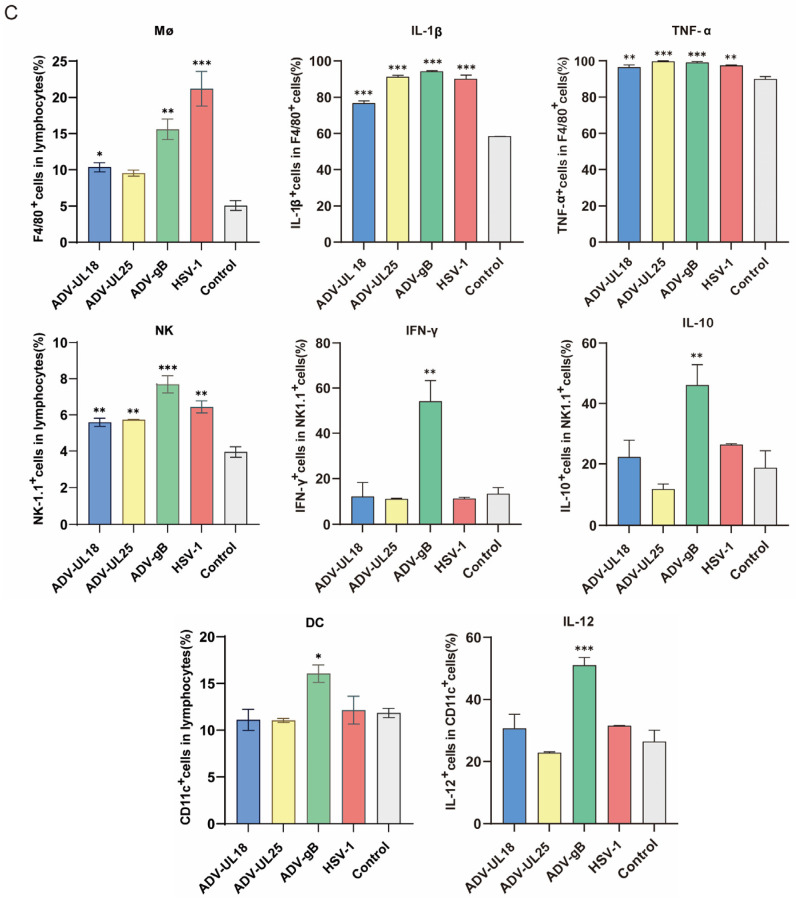
Innate immune response triggered by UL18, UL25, and gB in mice. (**A**) Heatmap showing the expression of innate immune factors at different time points after inoculation with recombinant adenovirus. The data for each group were compared with the control group (inoculated with blank adenoviral vector). (**B**) Expression of nine major factors at different time points after inoculation with recombinant adenovirus. The comparative Ct (ΔΔCt) method was utilized to standardize the relative mRNA expression levels in the experimental group to those of the mock group (the naïve mice group) at each time point. The Scheir–Ray–Hare test was conducted. Data are presented as the mean ± SD based on three independent experiments. ▲, Control vs. Mock. *, ADV-UL18 vs. Mock. ▼, ADV-UL25 vs. Mock. ●, ADV-gB vs. Mock. ▲ *p* < 0.05, ▲▲ *p* < 0.01, ▲▲▲ *p* < 0.001; * *p* < 0.05, ** *p* < 0.01, *** *p* < 0.001; ▼ *p* < 0.05, ▼▼ *p* < 0.01, ▼▼▼ *p* < 0.001; ● *p* < 0.05, ●● *p* < 0.01, ●●● *p* < 0.001. (**C**) The percentage of innate immune cells among total lymphocytes in the spleen of mice and the factors they secrete on the third day after inoculated with recombinant adenovirus. The data for each group were compared with the control group (inoculated with blank adenoviral vector). * *p* < 0.05, ** *p* < 0.01, *** *p* < 0.001.

It was essential to address any potential safety concerns related to the recombinant adenovirus. We monitored and recorded the clinical symptoms, body weight, and survival status of mice following the intranasal injection of the recombinant adenovirus (7 × 10^9^ VP/mouse). The results showed that mice inoculated intranasally with the recombinant adenovirus did not display any serious adverse reactions (Supplementary Figure S1). This indicates that these recombinant adenoviruses can be used for further research on the immune characteristics of the target proteins in vivo. Viral infections prompt the host to secrete various innate immune cytokines and chemokines, recruit immune cells, trigger an inflammatory response, and initiate an adaptive immune response against the viruses. To assess the impact of HSV-1 UL18, UL25, and gB on innate immune response levels, we used qRT-PCR to detect changes in the mRNA levels of several innate immune factors and flow cytometry to analyze changes in innate immune cells and their secretory factors after mice were inoculated with the recombinant adenovirus via the nasal cavity. In the lymph nodes (superficial cervical, axillary, brachial, and inguinal), the results indicated that UL18, UL25, and gB significantly upregulated certain factors, including antiviral immune activation-related signaling molecules (such as IFN-α and IL-22), inflammatory response-related signaling molecules (such as IL-9 and CCL-28), and chemokines (such as CXCL-11) (Figure 3A). Additionally, both UL18 and gB significantly enhanced the expression of IFN-β, IL-4, IL-23a, and GM-CSF as antiviral immune activation-related signaling molecules, as well as CXCL-11 as a chemokine (Figure 3B). These findings suggest that UL18, UL25, and gB can activate the antiviral immune response and increase the expression levels of inflammatory factors and chemokines in vivo. Among these proteins, the innate immune response induced by UL18 appeared earliest, peaking for these nine factors around the fifth day, while gB peaked later, around the seventh day. Throughout the entire observation period, the innate immune response triggered by UL25 remained relatively mild and did not show a significant peak (Figure 3B). In the spleens, the proportions of innate immune cells varied among different groups. UL18 and gB significantly increased the proportion of macrophages (Mø), while UL18, UL25, and gB all promoted macrophages to secrete IL- 1β and TNF-α. Additionally, UL18, UL25, and gB significantly increased the proportion of natural killer (NK) cells; however, only gB promoted NK cells to secrete IFN-γ and IL-10. gB also increased the proportion of dendritic cells (DC) and stimulated the secretion of IL-12 (Figure 3C). These results suggest that UL18, UL25, and gB can enhance the level of the innate immune response to a certain extent.

### 2.4. T- and B-Cell Subsets Affected by UL18, UL25, and gB Proteins

**Figure 4:**
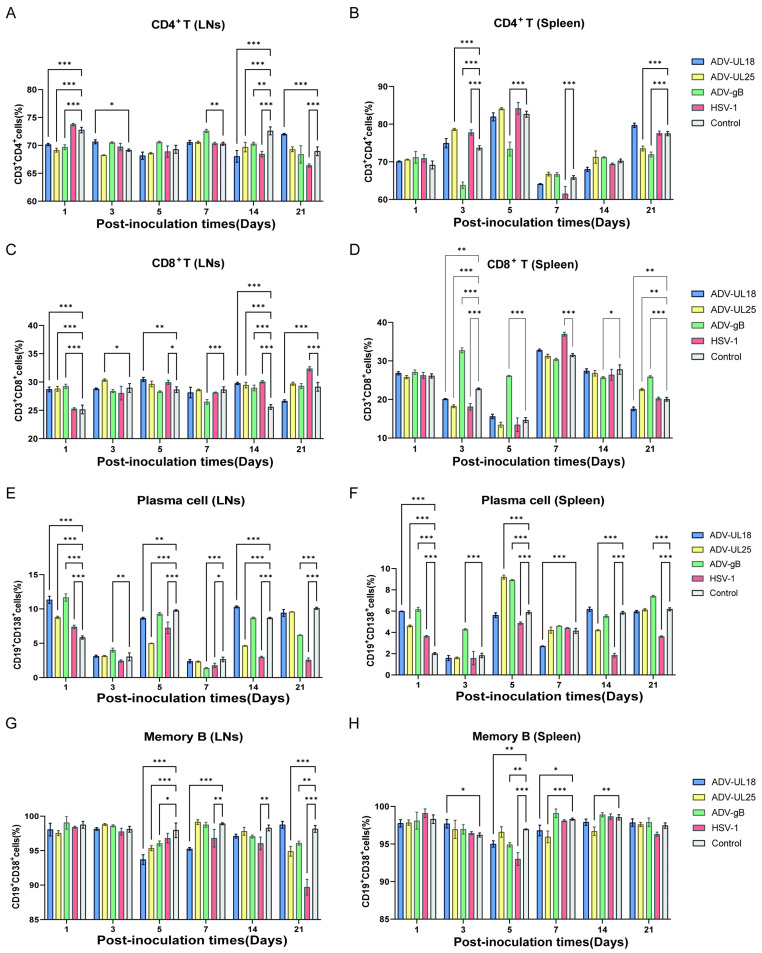
Effects of UL18, UL25, and gB on T- and B-cell subsets in mice. (**A**) Percentage of CD4+ T cells out of total T cells in the lymph nodes. (**B**) Percentage of CD4+ T cells out of total T cells in the spleen. (**C**) Percentage of CD8+ T cells out of total T cells in the lymph nodes. (**D**) Percentage of CD8+ T cells out of total T cells in the spleen. (**E**) Percentage of plasma cells out of total B cells in the lymph nodes. (**F**) Percentage of plasma cells out of total B cells in the spleen. (**G**) Percentage of memory B cells out of total B cells in the lymph nodes. (**H**) Percentage of memory B cells out of total B cells in the spleen. The Scheir–Ray–Hare test was conducted. Data are presented as the mean ± SD based on data from two independent experiments. Data at each time point in each group were compared with those of the control group (inoculated with a blank adenovirus vector). * *p* < 0.05, ** *p* < 0.01, *** *p* < 0.001.

In addition to chemokines and cytokines related to the innate immune response, UL18, UL25, and gB may also affect the antiviral effects of T and B cells. In the lymph nodes, a noticeable trend was observed: CD4+ T cells decreased, while CD8+ T cells increased in all three groups expressing the Ad-delivered target proteins on the first day of inoculation. However, the proportions of CD4+ and CD8+ T cells in each group remained relatively stable at subsequent time points (Figure 4A,C). Similarly, while the proportion of plasma cells increased in the three groups on the first day post-inoculation, changes in each group at later time points were largely stable or even suppressed compared with the control group (Figure 4E). From spleen lymphocytes, we found that gB significantly reduced the proportion of CD4+ T cells while increasing the proportion of CD8+ T cells between days 3 and 5 post-inoculation. By day 21, there was a slight decrease in CD4+ T cells and an increase in CD8+ T cells (Figure 4B,D). Additionally, gB substantially elevated plasma cell proportions from days 1 to 5 post-inoculation and showed a slight increase by day 21 (Figure 4F).

Interestingly, in both the lymph nodes and spleen, the HSV-1-infected group exhibited a notable decrease in plasma cell proportions between days 14 and 21 post-infection, while the protein transfection groups did not show this decline, remaining similar to the control group (except for the ADV-UL25 group on day 14) (Figure 4E,F). Furthermore, in the lymph nodes, the HSV1-infected group significantly reduced plasma cell proportions on day 21, while the protein transfection groups were generally comparable to the control group (Figure 4G). These findings suggest that these three proteins may influence antiviral effectiveness by affecting T- and B-cell subsets.

### 2.5. Specific T-Cell Responses Induced by UL18 and gB Protein in Mice

**Figure 5:**
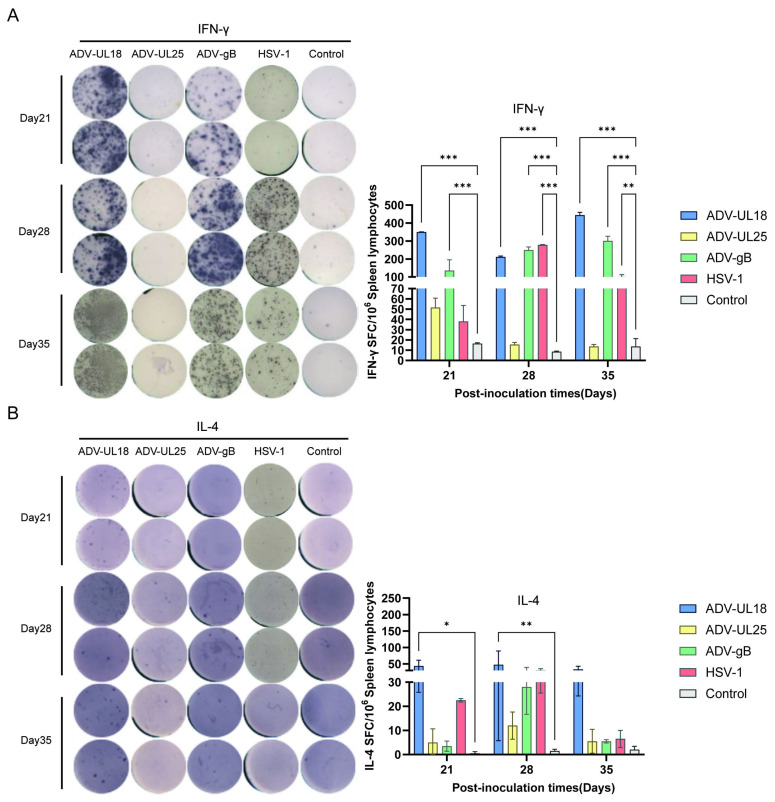
Analysis of specific T-cell responses elicited by UL18, UL25, and gB using ELISpot assay. (**A**) Statistical analysis of spots (left) and numbers (right) in each well on the IFN-γ ELISpot plate. (**B**) Statistical analysis of spots (left) and numbers (right) in each well on the IL-4 ELISpot plate. Stimulants: UL18 recombinant protein (3 µg/well) in the ADV-UL18 group, UL25 recombinant protein (3 µg/well) in the ADV-UL25 group, gB recombinant protein (3 µg/well) in the ADV-gB group, UL18 + UL25 + gB recombinant proteins (1 µg/well per protein, total 3 µg/well) in the control group and HSV-1 group. The data at each time point in each group were compared with those of the control group (inoculated with a blank adenovirus vector). * *p* < 0.05, ** *p* < 0.01, *** *p* < 0.001.

To further evaluate the cellular immunity elicited by these three proteins, spleens from mice intranasally inoculated with ADV-UL18, ADV-UL25, and ADV-gB were collected on days 21, 28, and 35 to assess the specific T-cell responses induced by the target proteins using ELISpot. The findings show that both UL18 and gB can stimulate mice to generate antigen-specific IFN-γ-secreting T-cell proliferation (Figure 5A), which is a critical factor produced by Th1 cells. Additionally, UL18 can also induce antigen-specific IL-4-secreting T-cell proliferation (Figure 5B), a key factor secreted by Th2 cells. By contrast, UL25 did not promote the proliferation of either IFN-γ- or IL-4-secreting T cells. Notably, the capsid protein UL18 elicited a strong Th1 response and a moderate Th2 response, while the glycoprotein gB primarily induced a significant Th1 response.

### 2.6. Specific Antibody Responses Elicited by UL18, UL25, and gB Proteins in Mice

**Figure 6:**
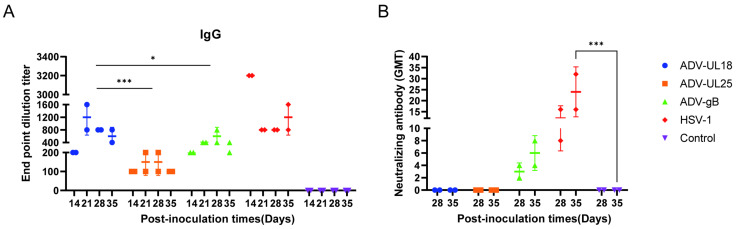
UL18, UL25, and gB can elicit antibody responses in mice. (**A**) Specific IgG antibodies were induced by three groups of recombinant adenoviruses. ELISA plate coating: The ADV-UL18 group was coated with UL18 protein, the ADV-UL25 group with UL25 protein, and the ADV-gB group with gB protein, while the control group and HSV-1 group were coated with UL18 + UL25 + gB protein. (**B**) Neutralizing antibodies against the HSV-1 McKrae strain were induced by three groups of recombinant adenoviruses. The data at each time point in each group were compared with those of the control group (inoculated with a blank adenovirus vector). * *p* < 0.05, *** *p* < 0.001.

To further explore the impact of the proteins encoded by UL18, UL25, and gB on antibody production in the humoral immune response of mice, sera were collected from mice intranasally inoculated with ADV-UL18, ADV-UL25, and ADV-gB on days 14, 21, 28, and 35 for specific IgG antibody detection via ELISA. Starting from day 14, all three proteins induced specific IgG antibodies. From days 21 to 28, the levels of specific IgG antibodies induced by UL18 (GMT ≥ 800) were significantly higher than those induced by UL25 (GMT ≤ 200) and gB (GMT ≤ 800), remaining elevated (GMT ≥ 400) until day 35. Furthermore, on day 21, the specific antibody level induced by UL18 (800 ≤ GMT ≤ 1600) surpassed that induced by HSV-1 (GMT = 800) (Figure 6A). Neutralizing antibodies were also evaluated on days 28 and 35. We observed that the specific antibodies induced by UL18 and UL25 were unable to neutralize the McKrae strain (GMT < 4). Only the ADV-gB or HSV infection could elicit neutralizing antibodies, as expected (Figure 6B).

These findings suggest that compared with UL25 and gB, UL18 induces higher levels of specific IgG in mice that persist longer but does not exhibit a neutralizing effect. Conversely, glycoprotein gB induces lower levels of neutralizing antibodies in mice.

### 2.7. Protection Elicited by UL18, UL25, and gB Proteins in Mice

**Figure 7:**
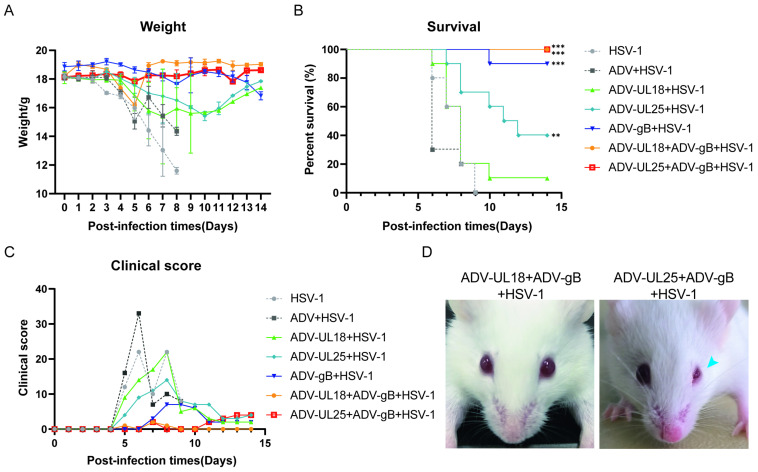

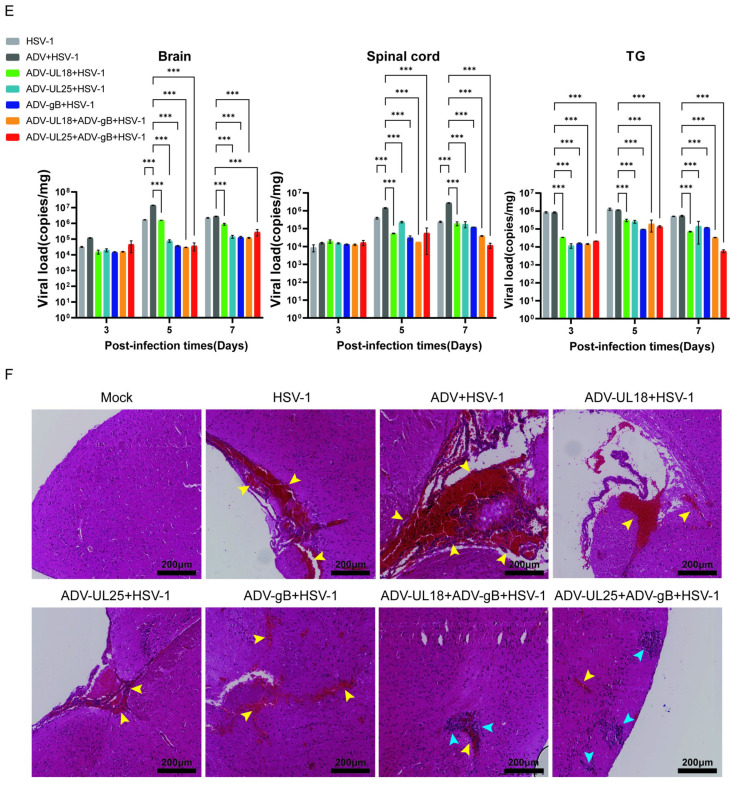
Protection elicited by UL18, UL25, and gB proteins in mice. (**A**) Changes in body weight of mice infected with HSV-1 (n = 10/group). (**B**) Survival rate of mice infected with HSV-1 (n = 10/group). The data of each group were compared with those of the HSV-1 group. ** *p* < 0.01, *** *p* < 0.001. (**C**) Total scores of clinical symptoms of HSV-1 infected mice in each group (0, healthy; 1, inverted hair/back arching; 2, blindness; 3, limb paralysis; 4, death) (n = 10/group). (**D**) Ocular symptoms of mice in the ADV-UL18 + ADV-gB and ADV-UL25 + ADV-gB groups after HSV-1 infection (blue arrow indicates blindness). (**E**) Viral load in the brain, spinal cord, and trigeminal ganglia (TG) of infected mice (n = 3/group). Data are presented as the mean ± SD from three independent mice. Data at each time point for each group were compared with those of the control group (ADV + HSV-1). *** *p* < 0.001. (**F**) Brain histopathology analysis by H&E staining (n = 3/group) (yellow arrow indicates tissue bleeding; blue arrow indicates inflammatory cell infiltration).

To evaluate the protective effects of the proteins encoded by UL18, UL25, and gB, mice inoculated with ADV-UL18, ADV-UL25, and ADV-gB were challenged with the wild HSV-1 McKrae strain on day 21 post-inoculation. Besides these groups, two co-immunization groups (ADV-UL18 + ADV-gB and ADV-UL25 + ADV-gB) were established simultaneously due to the immunogenicity of gB [27,28,29,30,31,36], which is the only component that can induce neutralizing antibody responses (Figure 6B). After infection with HSV-1, the weights of mice in the control groups (HSV-1, ADV + HSV-1), most of the single immunization groups (ADV-UL18, ADV-UL25) and one co-immunization group (ADV-UL18 + ADV-gB) showed a sharp decline starting from 3 d.p.i. By contrast, the mice in the single immunization group (ADV-gB) and the co-immunization group (ADV-UL25 + ADV-gB) continued to gain weight (Figure 7A). In terms of mice survival, deaths occurred in two control groups and three single immunization groups, but not in the two co-immunization groups, within 14 days of viral infection. The majority of deaths were concentrated between days 6 and 12 post-infection, with the mortality rates ranked from highest to lowest as follows: ADV + HSV-1 > HSV-1 > UL18 + HSV-1 > UL25 + HSV-1 > gB + HSV-1 > UL18 + gB + HSV-1/UL25 + gB + HSV-1 (Figure 7B). Except for the UL18 single immunization group, the mortality rate was reduced, and clinical symptoms were alleviated to varying degrees in the other single-immunization groups and all co-immunization groups (Figure 7B,C). During the 14-day HSV-1 challenge period, mice in the ADV-UL18 + ADV-gB co-immunization group showed no obvious symptoms, while 20% of the mice in the ADV-UL25 + ADV-gB co-immunization group exhibited mild ocular symptoms (Figure 7D). Additionally, the viral load in the brain, spinal cord, and trigeminal ganglia of mice in the immune and control groups was measured on days 3, 5, and 7 after the HSV-1 challenge. The results indicated that viral proliferation in these three tissues was slowed down in all treated groups compared with the control groups (HSV-1 and ADV + HSV-1). Notably, co-immunization with ADV-UL18 + ADV-gB or ADV-UL25 + ADV-gB appeared to have a more pronounced effect (Figure 7E). Histopathological observations of brain tissue on day 7 after HSV-1 infection revealed severe injury symptoms, such as massive hemorrhage in the meninges and choroid plexus, in the non-immunized group (HSV-1, ADV + HSV-1). Mice in the single immunization groups (ADV-UL18, ADV-UL25, ADV-gB) exhibited milder injury symptoms than those in the non-immunized group, including hemorrhage in the choroid plexus and meningeal or brain parenchyma. By contrast, mice in the combined immunization groups (ADV-UL18 + ADV-gB, ADV-UL25 + ADV-gB) showed only mild injury symptoms such as inflammatory cell infiltration, or minimal bleeding (Figure 7F). These results suggest that the combinations with gB appeared to be protective against HSV-1 challenge, although they did not seem to significantly enhance the results.

## 3. Discussion

The fusion of the HSV-1 envelope, the virus’s outermost structure, with the host cell membrane is essential for viral entry and the activation of the host immune system. Among the at least ten viral envelope proteins [37,38,39], gC and gD are the most frequently targeted due to their significant immunological roles, including the induction of neutralizing antibodies [40,41] and specific cytotoxic T-lymphocyte (CTL) responses [42,43,44]. Furthermore, analysis of the antigenic epitopes of gB and studies on its immune-related properties confirm that gB exhibits strong immunogenicity similar to that of gC and gD [27,28,30,36,45,46]. Notably, some reports suggest that gB and gD have the highest immunogenic potential [29]. In our study, we demonstrated that gB can induce specific IgG antibodies, achieving a geometric mean titer (GMT) of up to 800, as well as neutralizing antibodies with a GMT of approximately 6 at 35 days post-inoculation with recombinant adenovirus. Additionally, we observed a robust specific IFN-γ-secreting T-cell immune response. These findings indicate that the immune system can recognize HSV-1 envelope glycoproteins upon first exposure and trigger specific immune responses.

In addition to envelope proteins, the conservation of HSV nucleocapsid proteins and their essential roles in viral replication have prompted increased research into their functions in immune response modulation [23,47,48]. Our findings indicate that nucleocapsid proteins UL18 and UL25 can specifically stimulate IgG antibody production. This is consistent with previous studies identifying UL18 as containing an HLA B3503 CD8 epitope, while UL25 has two CD8 epitopes (HLA A0201 and B1402) and two HLA A2902-restricted CD8 epitopes [49,50,51,52]. Notably, among the proteins tested in our experiments, UL18 not only induced the highest levels of IgG but also maintained this response over time. Furthermore, UL18 surpassed gB in stimulating both specific IFN-γ-secreting T-cell proliferation and IL-4-secreting T-cell responses. Additionally, HSV-2 capsid protein UL25 has been reported to elicit CD8+ T-cell responses [49], while capsid protein VP5 (UL19) can induce CD4+ T-cell responses [53,54]. Collectively, these results suggest that HSV capsid proteins possess strong immunogenicity and could serve as candidate vaccine antigens.

Currently, HSV vaccine development largely focuses on humoral immunity, as most studies indicate that neutralizing antibodies effectively combat viral infections and vertical transmission [55,56]. Our research found that the specific neutralizing antibody response induced by gB can partially mitigate damage caused by lethal virus doses, reducing lethality from 100% in control groups to 10% in the gB group, along with alleviating tissue damage and clinical symptoms following HSV-1 infection. However, mice inoculated with nucleocapsid proteins UL18 and UL25 did not generate effective neutralizing antibodies, resulting in less significant protective effects against HSV-1 compared with those inoculated with ADV-gB. Nevertheless, mice receiving both envelope and nucleocapsid proteins showed no fatalities after lethal viral exposure, with lighter clinical symptoms and inflammatory responses than those receiving ADV-gB alone. This suggests that the immune response from nucleocapsid proteins complements that from envelope proteins. Notably, UL18 induced higher and more sustained specific IgG antibody levels than gB and stimulated a stronger cellular immune response. As a result, mice in the ADV-UL18 + ADV-gB group exhibited superior protection to those in the ADV-gB group and even better than those in the ADV-UL25 + ADV-gB group. Possible explanations for this observation are that UL18 is more abundant and widely distributed within HSV virus particles than UL25 or that UL18 is positioned in a way that makes it easier for the immune system to recognize compared with UL25 [57,58]. The challenges faced in HSV vaccine development indicate that relying solely on neutralizing antibodies—particularly those induced by glycoproteins—may not suffice for preventing HSV-related diseases or controlling recurrences [59,60,61,62,63]. Our findings highlight the importance of considering immune responses beyond neutralizing antibodies, such as cellular immunity, in designing effective HSV vaccines [64]. Evidence from various studies supports the critical role of cellular immunity in limiting or preventing HSV infection, replication, or pathogenicity [65,66,67,68,69,70,71,72,73]. Therefore, our strategy of combining envelope and nucleocapsid proteins—leveraging both humoral immunity (especially neutralizing antibodies) and cellular immunity—may offer a promising approach for HSV vaccine development.

## 4. Materials and Methods

### 4.1. Cells and Viruses

Human embryonic kidney cells (293T cells) and African green monkey kidney cells (Vero cells) were supplied by the Virus Immunology Laboratory of the Institute of Medical Biology, Chinese Academy of Medical Sciences. The 293T cells were cultured in Dulbecco’s modified Eagle’s medium (DMEM; Gibco, Grand Island, NY, USA) supplemented with 10% fetal bovine serum (FBS; Sigma, St. Louis, MO, USA), while the Vero cells were cultured in Eagle’s medium (MEM, Thermo Fisher Scientific, Waltham, MA, USA) with 10% newborn bovine serum (NBS; Sigma, St. Louis, MO, USA). The cells were maintained in a cell culture incubator at 37 °C with 5% CO_2_. Recombinant adenoviruses containing the target protein and carrying the EGFP tracer fluorescent protein named pAV-UL18-3 × GS-EGFP (ADV-UL18), pAV-UL25-3 × GS-EGFP (ADV-UL25), and pAV-gB-3 × GS-EGFP (ADV-gB) were constructed, packaged, and purified by Yunzhou Biotechnology (Guangzhou, China). The process was as follows. The target gene (UL18/UL25/gB) was connected with the EGFP gene with a 3 × GS linker and inserted between the Kozak (translation initiation sequence) and TKpA (Herpes simplex virus thymidine kinase polyadenylation signal) on the pAV[Exp]-EF1A transfer plasmid (an adenovirus transfer vector containing the human eukaryotic translation elongation factor 1 α1 promoter (EF1A) for the expression of the target gene). The transfer plasmid carrying the target gene was linearized by endonuclease PacI, and then the linearized plasmid was transfected into packaging cells to produce recombinant adenovirus particles. The obtained adenovirus particles were again transduced into packaging cells for amplification, and finally, the adenovirus particles were collected and further purified and concentrated by cesium chloride (CsCl) density gradient centrifugation. The HSV-1 McKrae strain was acquired from the Virus Immunology Laboratory of the Institute of Medical Biology, Chinese Academy of Medical Sciences.

### 4.2. Viral Transduction

The 293T cells were seeded into a 6-well plate at a density of 3 × 10^5^ cells per well and maintained at 37 °C with 5% CO_2_ until reaching 30–50% confluence. Subsequently, the 293T cells were transduced with a recombinant adenovirus (containing the target protein and carrying the EGFP tracer fluorescent protein) at a concentration of 4.5 × 10^8^ VP per well and incubated at 37 °C with 5% CO_2_. Fluorescence was observed using a fluorescence microscope (Nikon, Tokyo, Japan) 24 h post-transduction.

### 4.3. Western Blot

The cells were lysed by RIPA (Solarbio, Beijing, China) to extract total protein. The proteins were separated by 4–20% SDS-PAGE (SurePage; GenScript, Nanjing, China) and subsequently transferred to a PVDF membrane. The PVDF membrane was blocked with 5% skim milk (BD, Sparks, MD, USA), incubated with an anti-eGFP antibody (Mouse mAb) (ABclonal, Wuhan, China), and then probed with horseradish peroxidase (HRP)-conjugated goat anti-mouse IgG (H + L) (Beyotime, Shanghai, China). Finally, the PVDF membrane was treated with ECL ultrasensitive chemiluminescence reagent (Beyotime, Shanghai, China) and exposed using a gel imager (Bio-Rad, Hercules, CA, USA).

### 4.4. Virus Titer Determination

Infectivity titers were expressed as 50% cell culture infectious doses (CCID_50_) per milliliter. CCID_50_ assays were performed in 96-well plates. Vero cells were seeded into a 96-well plate at a density of 3 × 10^5^ cells per well (100 μL) and incubated in a 37℃, 5% CO_2_ cell culture incubator overnight. The viruses were serially diluted. Various dilutions of the virus solution (100 μL per well) were added to the plate containing the cells and then cultured at 37℃ with 5% CO_2_ for 7 days. Eight parallel wells were set up for each dilution. Cytopathic effects (CPEs) were observed and documented using an inverted microscope (Nikon, Tokyo, Japan). The CCID_50_ per milliliter was determined using the Reed and Muench method, as described previously [74], and the unit of calculated virus titer was CCID_50_/mL.

### 4.5. Animals and Ethics

All 6-week-old female BALB/c mice (SPF grade, weighing 15–17 g) were procured from Vital River (Beijing, China). The animals were provided with sufficient food and water and received care from veterinarians at CAMS IMB. All animal experiments underwent review and approval by the CAMS IMB Institutional Animal Care and Use Committee (approval number: DWSP202312015).

### 4.6. Animal Inoculation

Mice were intranasally inoculated with recombinant adenoviruses ADV-UL18, ADV-UL25, ADV-gB, and an empty adenovirus vector (Control) at a dosage of 7 × 10^9^ VP per mouse (10 μL). The positive control group (HSV-1) was intranasally infected with HSV-1 McKrae at a dosage of 2 × 10^4^ CCID_50_ per mouse (10 μL). The blank control group (Mock) was intranasally administered PBS (10 μL per mouse). Lymph nodes (superficial cervical, axillary, brachial, and inguinal), spleens, and serum samples were collected from the mice on days 1, 3, 5, 7, 14, 21, 28, and 35 post-inoculation (n = 3/group).

### 4.7. Small Animal In Vivo Imaging (Bioluminescence Imaging)

The inoculated mice were anesthetized with 2% isoflurane (RWD, Shenzhen, China) on days 1, 3, 5, 7, and 14 and positioned on the imaging platform. The mice were subjected to imaging with an exposure time of 30 s, ensuring that the acquired signal fell within the detection range. Bioluminescence intensity was quantified by measuring the total emission flux (photons/second) in the region of interest using the In Vivo Imaging Software (Bruker, Billerica, MA, USA) (n = 3/group).

### 4.8. qRT-PCR

Total RNAs from mouse lymph nodes (superficial cervical, axillary, brachial, and inguinal) were extracted using TRIzol (TaKaRa, Beijing, China). Following the protocol, primers specific for UL18, UL25, and gB in HSV-1 and cytokines (Supplementary Table S1) were designed for qRT-PCR. The qRT-PCR was conducted using the One Step TB Green^®^ PrimeScript™ PLUS RT-PCR Kit (TaKaRa, Beijing, China), with the following cycling conditions: 42 °C for 5 min, 95 °C for 5 s, and 60 °C for 20 s for 40 cycles. Total DNA was extracted from tissue samples from the experimental mice with an Axygen^®^ AxyPrep Body Fluid Viral DNA/RNA Miniprep Kit (Axygen^®^, Union City, CA, USA). According to the protocol, the primers used for q-RT-PCR were designed to be specific for the gD sequences in the HSV-1 genome (Supplementary Table S1). qRT-PCR was performed using the Takara Premix Ex Taq™ (Probe qPCR) Kit (TaKaRa, Beijing, China) with the following cycling conditions: 95 °C for 30 s, 95 °C for 5 s, and 60 °C for 30 s for 40 cycles.

### 4.9. Immunofluorescence

Lymph nodes (superficial cervical, axillary, brachial, and inguinal) were embedded in an OCT embedding agent (Tissue-Tek OCT Compound 4583; Sakura, Shanghai, China) and rapidly frozen in liquid nitrogen. They were then sectioned into 3 μm-thick slices using a freezing microtome (Leica, Wetzlar, Germany). The sections were fixed with a 4% paraformaldehyde solution (Servicebio, Wuhan, China), mounted with an anti-fade fluorescence mounting medium containing DAPI (Abcam, Cambridge, UK), and examined using confocal microscopy (TCS SP8; Leica, Wetzlar, Germany).

### 4.10. Isolate and Obtain Lymphocytes

Lymphocytes from the mouse spleen and lymph nodes (superficial cervical, axillary, brachial, and inguinal) were isolated following the instructions provided with the mouse lymphocyte isolation medium (Dakewe Biotech, Beijing, China). Red blood cells were eliminated, and the isolated cells were washed and resuspended in RPMI-1640 medium (Gibco, Grand Island, NY, USA) supplemented with 2% heat-inactivated fetal bovine serum (FBS; Sigma, St. Louis, MO, USA).

### 4.11. Flow Cytometry Analysis

The lymphocytes were aliquoted into tubes at 1 × 10^6^ cells (100 μL per tube), and 1 μL of each of the following flow cytometry antibodies was added: FITC-CD3 (Cat#100204; BioLegend, San Diego, CA, USA), PE-CD4 (Cat#116006; BioLegend, San Diego, CA, USA), APC-CD8α (Cat#100712; BioLegend, San Diego, CA, USA), PE/Cy7-CD19 (Cat#152418; BioLegend, San Diego, CA, USA), BV421-CD38 (Cat#102732; BioLegend, San Diego, CA, USA), APC-CD138 (Cat#142506; BioLegend, San Diego, CA, USA), F4/80-AF700 (Cat#123130; BioLegend, San Diego, CA, USA), CD11c-PE (Cat#557401; BioLegend, San Diego, CA, USA), and NK-1.1-APC-Cy7 (Cat#108724; BioLegend, San Diego, CA, USA). The samples were incubated at 4 °C for 30 min in the dark and were fixed and permeabilized with a BD Cytofix/Cytoperm™ Fixation/Permeabilization Kit (BD, Sparks, MD, USA). Then, 1 μL of each of the following flow cytometry antibodies was added: IL-10-PE (Cat#505008; BioLegend, San Diego, CA, USA), TNF-α-APC (Cat#506308; BioLegend, San Diego, CA, USA), GM-CSF-PE (Cat#554406; BioLegend, San Diego, CA, USA), IFN-γ-PerCP-Cy5.5 (Cat#505822; BioLegend, San Diego, CA, USA), IL-1beta-PE (Cat#12-7114-82; Invitrogen, MA, USA), and IL-12-AF488 (Cat#53-7123-82; Invitrogen, MA, USA). Subsequently, the cells were washed two times, transferred to a new tube, and analyzed using a flow cytometer (BD, Sparks, MD, USA). 

### 4.12. ELISpot Assay

ELISpot assays were performed using a mouse IFN-γ/IL-4 ELISpot kit (Mabtech, Cincinnati, OH, USA) according to the manufacturer’s protocol. First, lymphocytes were extracted from mouse spleens and seeded at a density of 1 × 10^6^ cells (100 μL) into each well of an ELISpot plate pre-coated with IFN-γ and IL-4. Purified UL18, UL25 (partial), and gB (partial) recombinant proteins (Cusabio, Wuhan, China) at a concentration of 3 μg/3 μL/well and positive stimulator phytohemagglutinin (PHA) at a concentration of 0.25 μg/10 μL/well were added to the ELISpot plate pre-coated with IFN-γ and IL-4. Only RPMI-1640 medium (Gibco, Grand Island, NY, USA) was added as a blank control. The culture dish, covered with tin foil, was then placed in a CO_2_ incubator at 37 °C and 5% CO_2_ for 36 h. Subsequently, the cells were discarded, and specific antibodies and enzyme-labeled avidin ALP were added to visualize the spots according to the protocol of the IFN-γ/IL-4 ELISpot kit. The colored spots were counted using an ELISpot reader (CTL, Shaker Heights, OH, USA).

### 4.13. ELISA

The ELISA plates (Corning Costar; Corning, NY, USA) were coated with UL18, UL25 (partial), and gB (partial) recombinant proteins (Cusabio, Wuhan, China) at a concentration of 0.1 μg per well (100 μL) and left to incubate overnight at 4 °C. During the experiment, the ELISA plates were blocked with 5% BSA–phosphate-buffered saline (PBS), then incubated with serially diluted serum samples (100 μL per well) at 37 ℃ for 1 h. The plates were visualized by reacting with an HRP-conjugated goat anti-mouse IgG (H + L) antibody (Beyotime, Shanghai, China) and TMB substrate (100 µL, Solarbio, Beijing, China). The absorbance of each well at 450 nm was measured using an ELISA plate reader (Gene Company, Beijing, China). Samples with OD values at least 2.1 times higher than the negative control were considered positive. Endpoint titers were determined as the highest serum dilutions producing positive OD values. Geometric mean titers (GMTs) were calculated as the geometric mean of the endpoint titers of positive serum samples in each group.

### 4.14. Neutralizing Antibody Assay

Serum samples were heat-inactivated at 55 ℃ for 30 min, then serially diluted 2-fold and co-incubated with live virus (100 CCID_50_ per well, 50 μL) for 2 h at 37 °C with 5% CO_2_. Following the incubation, 2 × 10^4^ Vero cells (100 μL) were added to each well containing serum-virus mixture and were further incubated at 37 °C with 5% CO_2_ for 7 days. Simultaneously, the experiment included a negative control group with only Vero cells and a virus control group with both Vero cells and HSV-1. Cytopathic effects (CPEs) were examined using an inverted microscope (Nikon Solutions Co., Ltd., Tokyo, Japan) to determine serum-neutralizing antibody titers. Geometric mean titers (GMTs) of neutralizing antibodies were calculated. The results of the neutralizing antibody measurement were obtained when cells in the negative control well and the virus control well at a dilution of 10^−2^ did not exhibit CPEs, while all cells in the original virus concentration well displayed CPEs. Additionally, cells in the virus control well at a dilution of 10^−1^ showed approximately 20–70% CPEs.

### 4.15. Immunization and Viral Challenge

Mice were randomly divided into seven groups and intranasally immunized with ADV (an empty adenovirus vector), recombinant adenoviruses ADV-UL18, ADV-UL25 and ADV-gB (7 × 10^9^ VP/10μL/mouse), ADV-UL18 + ADV-gB (3.5 × 10^9^ VP ADV-UL18 + 3.5 × 10^9^ VP ADV-gB/10 μL/mouse), and ADV-UL25 + ADV-gB (3.5 × 10^9^ VP ADV-UL25 + 3.5 × 10^9^ VP ADV-gB/10 μL/mouse). The blank control group received intranasal administration of PBS (10μL/mouse). At 21 days post-immunization, all mice were challenged with the HSV-1 wild-type strain McKrae (2 × 10^4^ CCID_50_/10 μL/mouse) via the intranasal route. Mice were weighed and observed for clinical symptoms daily (n = 10/group). The survival rate was assessed, and clinical scores were evaluated on the basis of an established scoring criterion (0, healthy; 1, inverted hair/back arching; 2, blindness; 3, limb paralysis; 4, death) over a 14-day period (n = 10/group). Tissue samples were obtained at 3, 5, and 7 days post-viral challenge for assessments of viral load and organ pathology (n = 3/group).

### 4.16. Histopathological Examination

Mouse organs were fixed in 10% formalin and embedded in paraffin to create tissue blocks. Approximately two slides per organ were stained with hematoxylin and eosin (H&E) to assess morphology.

### 4.17. Statistical Analysis

All data are presented as the mean ± standard deviation (SD). The Scheirer–Ray–Hare test in SPSS 25.0, one-way ANOVA, or two-way ANOVA (and nonparametric or mixed methods in GraphPad Prism 9.5) were performed to determine the *p*-values. The level of statistical significance is indicated by asterisks (* *p*  <  0.05, ** *p*  <  0.01, *** *p*  <  0.001). Charts and graphs were created using GraphPad Prism 9.5.

## Data Availability

All authors declare that the data files used in the current study can be made available upon reasonable request.

## Supplementary Materials

The following supporting information can be downloaded at: https://www.mdpi.com/article/10.3390/ijms252413486/s1.
