# Supplementary material for: Immune Response Elicited by Recombinant Adenovirus-Delivered Glycoprotein B and Nucleocapsid Protein UL18 and UL25 of HSV-1 in Mice
Source: Int J Mol Sci. 2024 Dec 16;25(24):13486. doi: 10.3390/ijms252413486 (PMC11678876; doi:10.3390/ijms252413486)
Supplement: Supplementary file 1 [file ijms-25-13486-s001.zip › Supplementary Files/Supplement FigureS1 and Table S1.pdf]

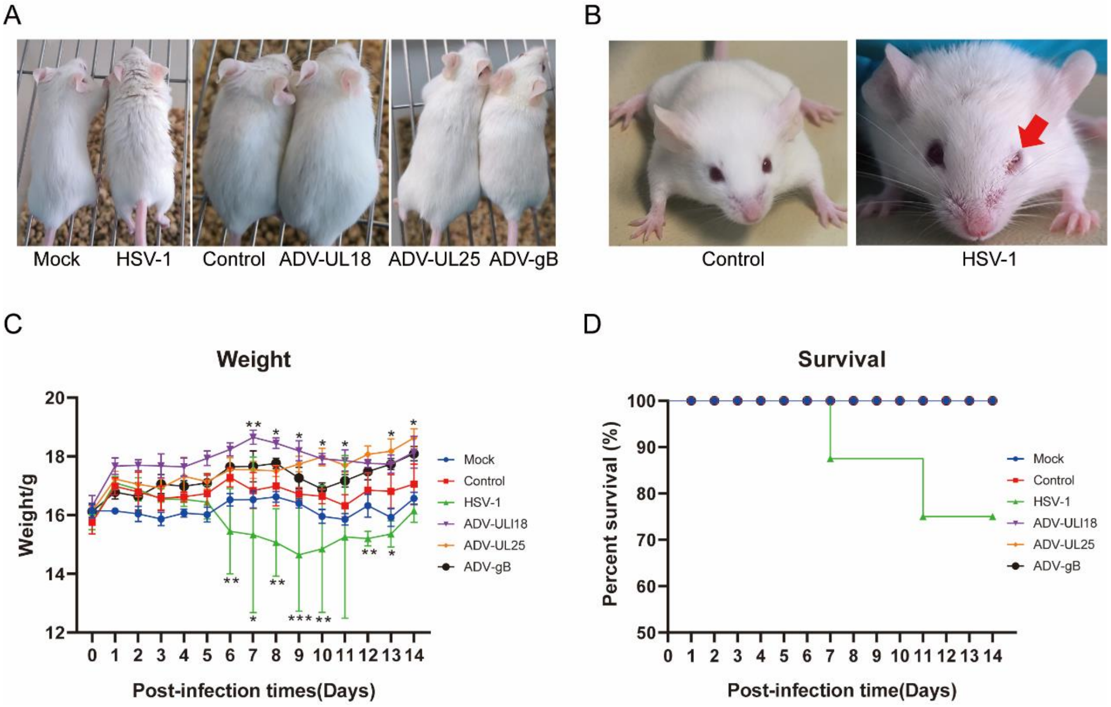

Figure S1. Clinical symptoms of mice infected with recombinant adenovirus ADV-UL18, ADV-UL25, and ADV-gB. (**A**) Mice infected with recombinant adenovirus did not show arched backs or erect hair. (**B**) Typical eye inflammation (marked by red arrows) in mice infected with HSV-1. (**C**) Body weight changes of mice infected with recombinant adenovirus. The body weights of each group at each time point were compared with those of the Control group (infected with blank adenovirus vector). * p<0.05, ** p<0.01, *** p<0.001. (**D**) The survival rate of mice infected with recombinant adenovirus remained unchanged. n=8.

Table S1. The primer sequence of qRT-PCR

Gene	Sequence（5'-3'）
UL18	F: CGGACGGCTTTGAAACTGAC<br>R: ATGGTCGGCAGGAATACCAC
UL25	F: GACCCGTACTGCCCATTTGA<br>R: GAGGTTAAAGACGGGCGACA
gB	F: CCGACCTCAAGTACAACCCC<br>R: GTAGCCGTAAAACGGGGACA
GAPDH	F: AGGTCGGTGTGAACGGATTTG<br>R: TGTAGACCATGTAGTTGAGGTCA
IFN-α	F CTTCCTCAGACTCATAACCT<br>R: AGTCCTTCCTGTCCTTCA
IFN-β	F: GATGAACTCCACCAGCAGACAGTG<br>R: CACCATCCAGGCGTAGCTGTTG
IFN-γ	F: ATCAGGCCATCAGCAACAACA<br>R: CGTCTCACCTCAAACTTGGCA
TNF-a	F: GCCAACGGCATGGATCTCAA

	R: TCTTGACGGCAGAGAGGAGG
IL-1β	F: GCAACTGTTCCTGAACTCAACCT<br>R: ATCTTTTGGGGTCCGTCAAC
CD160	F: CCTGAGACCAACTTAGAACA<br>R: ACACCAACTGAGATGACTT
OX4OL	F: TGCTTCTGTGCTTCATCTAT<br>R: ATCTGGTAACTGCTCCTCT
BTLA	F: GCCAGGACAGGAGAGTTA<br>R: CTTACACCAAGTCACATTAGG
RANKL	F: TCACTCTGTCCTCTTGGTA<br>R: CAGGTAATAGAAGCCATCTTG
LIGHT	F: TTCTGAGCACCTACATTCC<br>R: CTTCTGACCAACCATTCCT
GMCSF	F: GGCCTTGGAAGCATGTAGAGG<br>R: GGAGAACTCGTTAGAGACGACTT
IL-2	F: GAGCAGGATGGAGAATTACAGG<br>R: GTCCAAGTTCATCTTCTAGGCAC
IL-4	F: GTGAGCTCGTCTGTAGGGCT<br>R: CCGCTTACCGATGAATCCAGG
IL-5	F: CTCTGTTGACAAGCAATGAGACG<br>R: TCTTCAGTATGTCTAGCCCCTG
IL-6	F: TAGTCCTTCCTACCCCAATTTCC<br>R: TTGGTCCTTAGCCACTCCTTC
IL-9	F: ATGTTGGTGACATACATCCTTGC<br>R: GACGGTGGATCATCCTTCAG
IL-12	F: CCTCCTGTGGGAGAAGCAGA<br>R: CTTGAGCCTTTCAGGCGGAG
IL-13	F: CCTCATGGCGCTTITGTTGAC<br>R: TCTGGTTCTGGGTGATGTTGA
IL-17	F: AGATTACTACAACCGATCCACCT<br>R: GGGGACAGAGTTCATGTGGTA
IL-22	F: GCTTGACAAGTCCAACTTCCA<br>R: GCTCACTCATACTGACTCCGT
CCL-28	F: GTGTGTGGCTTTTCAAACCTCA<br>R: TGCATGAACTCACTCTTTCCAG
CXCL-12	F: TGCATCAGTGACGGTAAACCA<br>R: TTCTTCAGCCGTGCAACAATC
CXCL-2	F: CCAACCACCAGGCTACAGG<br>R: GCGTCACACTCAAGCTCTG
CXCL-11	F: GGCTTCCTTATGTTCAAACAGGG<br>R: GCCGTTACTCGGGTAAATTACA
CXCL-1	F: CTGGCCACAGGGGCG<br>R: CCTGAGGGCAACACCTTCAA
IL-23a	F: AATAATGTGCCCCGTATCCAGT<br>R: GCTCCCCTTTGAAGATGTCAG
4-1BBL	F: AAGCCTCAGGTAGATGACT<br>R: GAACGGTCCACTAACTTGT
IKK-α	F: TGGAAGAGACTGCTGACA<br>R: ATGAAGAACACTTGCTGAGA
IKK-β	F: CTCCGAAGATACTTGAACCA<br>R: CGATGCGATGTCACTCAG

TAK	F: CCATCACTTACACAGCAATC<br>R: CCTCACAGATACATACACAGA
HSV-1 gD	F: CCTGGTCATTTGCGGAATTG<br>R: GAGGCGTATGCGCTTTGG<br>Probe:5′-6FAM-ATGCGCCGCCACACTCAAAAAGC-BHQ1-3′

F: Forward primer; R: Reverse primer.
