# Supplementary material for: Immune Response Elicited by Recombinant Adenovirus-Delivered Glycoprotein B and Nucleocapsid Protein UL18 and UL25 of HSV-1 in Mice
Source: Int J Mol Sci. 2024 Dec 16;25(24):13486. doi: 10.3390/ijms252413486 (PMC11678876; doi:10.3390/ijms252413486)
Supplement: Supplementary file 1 [file ijms-25-13486-s001.zip › Supplementary Files/Vector Map and Sequence.pdf]

pAV-UL18-3×GS-EGFP(ADV-UL18)

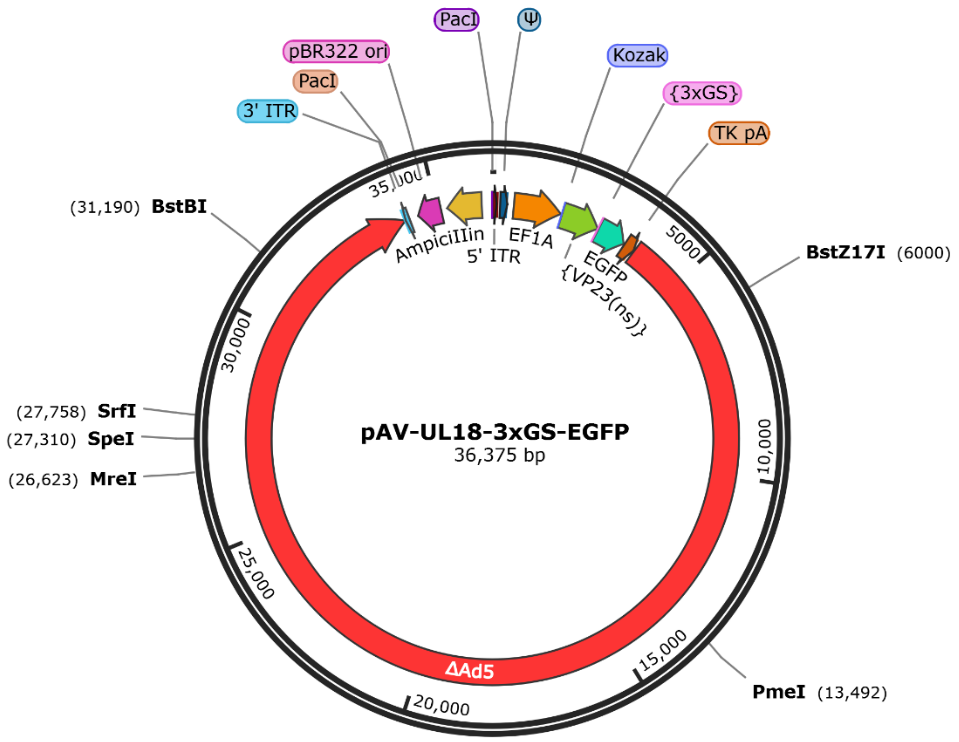

CATCATCAATAATATACCTTATTTTGGATTGAAGCCAATATGATAATGAGGGGGTGGAGTTTGTGACGTGGCGCGGGGCGT
GGGAACGGGGCGGGTGACGTAGTAGTGTGGCGGAAGTGTGATGTTGCAAGTGTGGCGGAACACATGTAAGCGACGGATG
TGGCAAAAGTGACGTTTTTGGTGTGCGCCGGTGTACACAGGAAGTGACAATTTTCGCGCGGTTTTAGGCGGATGTTGTAGT
AAATTTGGGCGTAACCGAGTAAGATTTGGCCATTTTCGCGGGAAAACTGAATAAGAGGAAGTGAAATCTGAATAATTTTGT
GTTACTCATAGCGCGTAATATTTGTCTAGGGCCGCGGGGACTTTGACCGTTTACGTGGAGACTCGCCCAGGTGTTTTTCTCA
GGTGTTTTCCGCGTTCCGGGTCAAAGTTGGCGTTTTATTATTATAGTCAGTCGAAGCTTGGATCCGGTACCTCTAGAATTCTC
GAGCGGCCGCTAGCGACATCGATCAACTTTGTATAGAAAAGTTGGGCTCCGGTGCCCGTCAGTGGGCAGAGCGCACATCG
CCCACAGTCCCCGAGAAGTTGGGGGGAGGGGTCGGCAATTGAACCGGTGCCTAGAGAAGGTGGCGCGGGGTAAACTGG
GAAAGTGATGTCGTGTACTGGCTCCGCCTTTTTCCCGAGGGTGGGGGAGAACCGTATATAAGTGCAGTAGTCGCCGTGAA
CGTTCTTTTTCGCAACGGGTTTGCCGCCAGAACACAGGTAAGTGCCGTGTGTGGTTCCCGCGGGCCTGGCCTCTTTACGGG
TTATGGCCCTTGCGTGCCTTGAATTACTTCCACCTGGCTGCAGTACGTGATTCTTGATCCCGAGCTTCGGGTTGGAAGTGGG
TGGGAGAGTTCGAGGCCTTGCGCTTAAGGAGCCCCTTCGCCTCGTGCTTGAGTTGAGGCCTGGCCTGGGCGCTGGGGCCG
CCGCGTGCGAATCTGGTGGCACCTTCGCGCCTGTCTCGCTGCTTTCGATAAGTCTCTAGCCATTTAAAATTTTTGATGACCT
GCTGCGACGCTTTTTTTCTGGCAAGATAGTCTTGTAAATGCGGGCCAAGATCTGCACACTGGTATTTCGGTTTTTGGGGCCG
CGGGCGGCGACGGGGCCCGTGCGTCCCAGCGCACATGTTCGGCGAGGCGGGGCCTGCGAGCGCGGCCACCGAGAATCG
GACGGGGGTAGTCTCAAGCTGGCCGGCCTGCTCTGGTGCCTGGTCTCGCGCCGCCGTGTATCGCCCCGCCCTGGGCGGC
AAGGCTGGCCCGGTCGGCACCAGTTGCGTGAGCGGAAAGATGGCCGCTTCCCGGCCCTGCTGCAGGGAGCTCAAAATGG
AGGACGCGGCGCTCGGGAGAGCGGGCGGGTGAGTCACCCACACAAAGGAAAAGGGCCTTTCCGTCCTCAGCCGTCGCTT
CATGTGACTCCACGGAGTACCGGGCGCCGTCCAGGCACCTCGATTAGTTCTCGAGCTTTTGGAGTACGTCGTCTTTAGGTT
GGGGGGAGGGGTTTTATGCGATGGAGTTTCCCCACACTGAGTGGGTGGAGACTGAAGTTAGGCCAGCTTGGCACTTGATG
TAATTCTCCTTGGAATTTGCCCTTTTTGAGTTTGGATCTTGGTTCATTCTCAAGCCTCAGACAGTGGTTCAAAGTTTTTTTCTT
CCATTTCAGGTGTCGTGACAAGTTTGTACAAAAAAGCAGGCTGCCACCATGCTGGCGGACGGCTTTGAAACTGACATCGC
GATACCCTCGGGCATCTCGCGCCCCGATGCGGCGGCGCTGCAGCGCTGCGAAGGGCGGGTGGTATTCCTGCCGACCATC
CGCCGGCAACTGACGCTGGCCGACGTGGCGCACGAATCCTTCGTCTCCGGAGGCGTCAGTCCCGACACGTTGGGGTTGTT
GCTGGCGTACCGAAGGCGCTTCCCCGCGGTCATCACCCGGGTGCTTCCCACGCGAATCGTCGCCTGCCCCCTGGACGTGG
GCCTCACCCACGCCGGCACCGTTAACCTTCGCAACACCTCCCCCGTAGATCTCTGTAACGGGGACCCCATCAGCCTCGTC

CCGCCCGTGTTCGAGGGCCAAGCGACGGACGTGCGCCTGGATTCGCTGGACCTCACGTTGCGGTTTCCCGTTCCGCTTCC
ATCGCCCCTGGCGCGCGAAATCGTGGCGCGGCTCGTGGCCAGGGGCATCCGGGACCTGAACCCCAGCCCCAGAAACCCC
GGAGGGCTGCCAGACCTCAACGTGCTGTACTACAACGGGAGTCGCCTCTCGCTGCTGGCGGACGTCCAACAACTCGGTCC
CGTAAACGCCGAGCTGCGATCGCTGGTCCTTAACATGGTTTACTCGATCACGGAGGGAACCACCATCATCCTTACGCTAAT
CCCCCGGCTCTTTGCGCTAAGTGCCCAGGACGGGTACGTGAACGCTCTACTGCAGATGCAGAGTGTCACGCGGGAGGCC
GCCCAGCTCATTCACCCCGAAGCCCCGGCCCTGATGCAGGATGGAGAGCGAAGGCTGCCGCTTTACGAGGCGCTCGTCG
CCTGGCTGACCCACGCGGGCCAACTAGGAGACACCTTGGCCCTGGCTCCCGTGGTTCGGGTGTGCACCTTTGACGGCGCG
GCCGTTGTGCGGTCCGGAGACATGGCCCCCGTTATACGCTATCCCGGCAGCGGCAGCGGCAGCATGGTGAGCAAGGGCG
AGGAGCTGTTCACCGGGGTGGTGCCCATCCTGGTCGAGCTGGACGGCGACGTAAACGGCCACAAGTTCAGCGTGTCCGG
CGAGGGCGAGGGCGATGCCACCTACGGCAAGCTGACCCTGAAGTTCATCTGCACCACCGGCAAGCTGCCCGTGCCCTGG
CCCACCCTCGTGACCACCCTGACCTACGGCGTGCAGTGCTTCAGCCGCTACCCCGACCACATGAAGCAGCACGACTTCTT
CAAGTCCGCCATGCCCGAAGGCTACGTCCAGGAGCGCACCATCTTCTTCAAGGACGACGGCAACTACAAGACCCGCGCC
GAGGTGAAGTTCGAGGGCGACACCCTGGTGAACCGCATCGAGCTGAAGGGCATCGACTTCAAGGAGGACGGCAACATCC
TGGGGCACAAGCTGGAGTACAACTACAACAGCCACAACGTCTATATCATGGCCGACAAGCAGAAGAACGGCATCAAGGT
GAACTTCAAGATCCGCCACAACATCGAGGACGGCAGCGTGCAGCTCGCCGACCACTACCAGCAGAACACCCCCATCGGC
GACGGCCCCGTGCTGCTGCCCGACAACCACTACCTGAGCACCCAGTCCGCCCTGAGCAAAGACCCCAACGAGAAGCGCG
ATCACATGGTCCTGCTGGAGTTCGTGACCGCCGCCGGGATCACTCTCGGCATGGACGAGCTGTACAAGTAAACCCAGCTT
TCTTGTACAAAGTGGTGGGGGAGGCTAACTGAAACACGGAAGGAGACAATACCGGAAGGAACCCGCGCTATGACGGCAA
TAAAAAGACAGAATAAAACGCACGGGTGTTGGGTCGTTTGTTCATAAACGCGGGGTTCGGTCCCAGGGCTGGCACTCTGT
CGATACCCCACCGAGACCCCATTGGGGCCAATACGCCCGCGTTTCTTCCTTTTCCCCACCCCACCCCCCAAGTTCGGGTGA
AGGCCCAGGGCTCGCAGCCAACGTCGGGGCGGCAGGCCCTGCCATAGCGATCGATTCGACAGATCACTGAAATGTGTGG
GCGTGGCTTAAGGGTGGGAAAGAATATATAAGGTGGGGGTCTTATGTAGTTTTGTATCTGTTTTGCAGCAGCCGCCGCCGC
CATGAGCACCAACTCGTTTGATGGAAGCATTGTGAGCTCATATTTGACAACGCGCATGCCCCCATGGGCCGGGGTGCGTC
AGAATGTGATGGGCTCCAGCATTGATGGTCGCCCCGTCCTGCCCGCAAACTCTACTACCTTGACCTACGAGACCGTGTCTG
GAACGCCGTTGGAGACTGCAGCCTCCGCCGCCGCTTCAGCCGCTGCAGCCACCGCCCGCGGGATTGTGACTGACTTTGCT
TTCCTGAGCCCGCTTGCAAGCAGTGCAGCTTCCCGTTCATCCGCCCGCGATGACAAGTTGACGGCTCTTTTGGCACAATTG
GATTCTTTGACCCGGGAACTTAATGTCGTTTCTCAGCAGCTGTTGGATCTGCGCCAGCAGGTTTCTGCCCTGAAGGCTTCCT
CCCCTCCCAATGCGGTTTAAAACATAAATAAAAAACCAGACTCTGTTTGGATTTGGATCAAGCAAGTGTCTTGCTGTCTTTA
TTTAGGGGTTTTGCGCGCGCGGTAGGCCCGGGACCAGCGGTCTCGGTCGTTGAGGGTCCTGTGTATTTTTTCCAGGACGTG
GTAAAGGTGACTCTGGATGTTCAGATACATGGGCATAAGCCCGTCTCTGGGGTGGAGGTAGCACCACTGCAGAGCTTCAT
GCTGCGGGGTGGTGTTGTAGATGATCCAGTCGTAGCAGGAGCGCTGGGCGTGGTGCCTAAAAATGTCTTTCAGTAGCAAG
CTGATTGCCAGGGGCAGGCCCTTGGTGTAAGTGTTTACAAAGCGGTTAAGCTGGGATGGGTGCATACGTGGGGATATGAG
ATGCATCTTGGACTGTATTTTTAGGTTGGCTATGTTCCCAGCCATATCCCTCCGGGGATTCATGTTGTGCAGAACCACCAGC
ACAGTGTATCCGGTGCACTTGGGAAATTTGTCATGTAGCTTAGAAGGAAATGCGTGGAAGAACTTGGAGACGCCCTTGTG
ACCTCCAAGATTTTCCATGCATTCGTCCATAATGATGGCAATGGGCCCACGGGCGGCGGCCTGGGCGAAGATATTTCTGG
GATCACTAACGTCATAGTTGTGTTCCAGGATGAGATCGTCATAGGCCATTTTTACAAAGCGCGGGCGGAGGGTGCCAGAC
TGCGGTATAATGGTTCCATCCGGCCCAGGGGCGTAGTTACCCTCACAGATTTGCATTTCCCACGCTTTGAGTTCAGATGGG
GGGATCATGTCTACCTGCGGGGCGATGAAGAAAACGGTTTCCGGGGTAGGGGAGATCAGCTGGGAAGAAAGCAGGTTCC
TGAGCAGCTGCGACTTACCGCAGCCGGTGGGCCCGTAAATCACACCTATTACCGGCTGCAACTGGTAGTTAAGAGAGCTG
CAGCTGCCGTCATCCCTGAGCAGGGGGGCCACTTCGTTAAGCATGTCCCTGACTCGCATGTTTTCCCTGACCAAATCCGCC
AGAAGGCGCTCGCCGCCCAGCGATAGCAGTTCTTGCAAGGAAGCAAAGTTTTTCAACGGTTTGAGACCGTCCGCCGTAGG
CATGCTTTTGAGCGTTTGACCAAGCAGTTCCAGGCGGTCCCACAGCTCGGTCACCTGCTCTACGGCATCTCGATCCAGCAT
ATCTCCTCGTTTCGCGGGTTGGGGCGGCTTTCGCTGTACGGCAGTAGTCGGTGCTCGTCCAGACGGGCCAGGGTCATGTCT
TTCCACGGGCGCAGGGTCCTCGTCAGCGTAGTCTGGGTCACGGTGAAGGGGTGCGCTCCGGGCTGCGCGCTGGCCAGGG
TGCGCTTGAGGCTGGTCCTGCTGGTGCTGAAGCGCTGCCGGTCTTCGCCCTGCGCGTCGGCCAGGTAGCATTTGACCATG
GTGTCATAGTCCAGCCCCTCCGCGGCGTGGCCCTTGGCGCGCAGCTTGCCCTTGGAGGAGGCGCCGCACGAGGGGCAGT
GCAGACTTTTGAGGGCGTAGAGCTTGGGCGCGAGAAATACCGATTCCGGGGAGTAGGCATCCGCGCCGCAGGCCCCGCA
GACGGTCTCGCATTCCACGAGCCAGGTGAGCTCTGGCCGTTCGGGGTCAAAAACCAGGTTTCCCCCATGCTTTTTGATGCG
TTTCTTACCTCTGGTTTCCATGAGCCGGTGTCCACGCTCGGTGACGAAAAGGCTGTCCGTGTCCCCGTATACAGACTTGAG

AGGCCTGTCCTCGAGCGGTGTTCCGCGGTCCTCCTCGTATAGAAACTCGGACCACTCTGAGACAAAGGCTCGCGTCCAGG
CCAGCACGAAGGAGGCTAAGTGGGAGGGGTAGCGGTCGTTGTCCACTAGGGGGTCCACTCGCTCCAGGGTGTGAAGACA
CATGTCGCCCTCTTCGGCATCAAGGAAGGTGATTGGTTTGTAGGTGTAGGCCACGTGACCGGGTGTTCCTGAAGGGGGGC
TATAAAAGGGGGTGGGGGCGCGTTCGTCCTCACTCTCTTCCGCATCGCTGTCTGCGAGGGCCAGCTGTTGGGGTGAGTAC
TCCCTCTGAAAAGCGGGCATGACTTCTGCGCTAAGATTGTCAGTTTCCAAAAACGAGGAGGATTTGATATTCACCTGGCCC
GCGGTGATGCCTTTGAGGGTGGCCGCATCCATCTGGTCAGAAAAGACAATCTTTTTGTTGTCAAGCTTGGTGGCAAACGAC
CCGTAGAGGGCGTTGGACAGCAACTTGGCGATGGAGCGCAGGGTTTGGTTTTTGTCGCGATCGGCGCGCTCCTTGGCCGC
GATGTTTAGCTGCACGTATTCGCGCGCAACGCACCGCCATTCGGGAAAGACGGTGGTGCGCTCGTCGGGCACCAGGTGC
ACGCGCCAACCGCGGTTGTGCAGGGTGACAAGGTCAACGCTGGTGGCTACCTCTCCGCGTAGGCGCTCGTTGGTCCAGCA
GAGGCGGCCGCCCTTGCGCGAGCAGAATGGCGGTAGGGGGTCTAGCTGCGTCTCGTCCGGGGGGTCTGCGTCCACGGTA
AAGACCCCGGGCAGCAGGCGCGCGTCGAAGTAGTCTATCTTGCATCCTTGCAAGTCTAGCGCCTGCTGCCATGCGCGGGC
GGCAAGCGCGCGCTCGTATGGGTTGAGTGGGGGACCCCATGGCATGGGGTGGGTGAGCGCGGAGGCGTACATGCCGCA
AATGTCGTAAACGTAGAGGGGCTCTCTGAGTATTCCAAGATATGTAGGGTAGCATCTTCCACCGCGGATGCTGGCGCGCA
CGTAATCGTATAGTTCGTGCGAGGGAGCGAGGAGGTCGGGACCGAGGTTGCTACGGGCGGGCTGCTCTGCTCGGAAGAC
TATCTGCCTGAAGATGGCATGTGAGTTGGATGATATGGTTGGACGCTGGAAGACGTTGAAGCTGGCGTCTGTGAGACCTA
CCGCGTCACGCACGAAGGAGGCGTAGGAGTCGCGCAGCTTGTTGACCAGCTCGGCGGTGACCTGCACGTCTAGGGCGCA
GTAGTCCAGGGTTTCCTTGATGATGTCATACTTATCCTGTCCCTTTTTTTTCCACAGCTCGCGGTTGAGGACAAACTCTTCGC
GGTCTTTCCAGTACTCTTGGATCGGAAACCCGTCGGCCTCCGAACGGTAAGAGCCTAGCATGTAGAACTGGTTGACGGCC
TGGTAGGCGCAGCATCCCTTTTCTACGGGTAGCGCGTATGCCTGCGCGGCCTTCCGGAGCGAGGTGTGGGTGAGCGCAAA
GGTGTCCCTGACCATGACTTTGAGGTACTGGTATTTGAAGTCAGTGTCGTCGCATCCGCCCTGCTCCCAGAGCAAAAAGTC
CGTGCGCTTTTTGGAACGCGGATTTGGCAGGGCGAAGGTGACATCGTTGAAGAGTATCTTTCCCGCGCGAGGCATAAAGT
TGCGTGTGATGCGGAAGGGTCCCGGCACCTCGGAACGGTTGTTAATTACCTGGGCGGCGAGCACGATCTCGTCAAAGCCG
TTGATGTTGTGGCCCACAATGTAAAGTTCCAAGAAGCGCGGGATGCCCTTGATGGAAGGCAATTTTTTAAGTTCCTCGTAG
GTGAGCTCTTCAGGGGAGCTGAGCCCGTGCTCTGAAAGGGCCCAGTCTGCAAGATGAGGGTTGGAAGCGACGAATGAGC
TCCACAGGTCACGGGCCATTAGCATTTGCAGGTGGTCGCGAAAGGTCCTAAACTGGCGACCTATGGCCATTTTTTCTGGGG
TGATGCAGTAGAAGGTAAGCGGGTCTTGTTCCCAGCGGTCCCATCCAAGGTTCGCGGCTAGGTCTCGCGCGGCAGTCACT
AGAGGCTCATCTCCGCCGAACTTCATGACCAGCATGAAGGGCACGAGCTGCTTCCCAAAGGCCCCCATCCAAGTATAGGT
CTCTACATCGTAGGTGACAAAGAGACGCTCGGTGCGAGGATGCGAGCCGATCGGGAAGAACTGGATCTCCCGCCACCAA
TTGGAGGAGTGGCTATTGATGTGGTGAAAGTAGAAGTCCCTGCGACGGGCCGAACACTCGTGCTGGCTTTTGTAAAAACG
TGCGCAGTACTGGCAGCGGTGCACGGGCTGTACATCCTGCACGAGGTTGACCTGACGACCGCGCACAAGGAAGCAGAGT
GGGAATTTGAGCCCCTCGCCTGGCGGGTTTGGCTGGTGGTCTTCTACTTCGGCTGCTTGTCCTTGACCGTCTGGCTGCTCGA
GGGGAGTTACGGTGGATCGGACCACCACGCCGCGCGAGCCCAAAGTCCAGATGTCCGCGCGCGGCGGTCGGAGCTTGAT
GACAACATCGCGCAGATGGGAGCTGTCCATGGTCTGGAGCTCCCGCGGCGTCAGGTCAGGCGGGAGCTCCTGCAGGTTT
ACCTCGCATAGACGGGTCAGGGCGCGGGCTAGATCCAGGTGATACCTAATTTCCAGGGGCTGGTTGGTGGCGGCGTCGAT
GGCTTGCAAGAGGCCGCATCCCCGCGGCGCGACTACGGTACCGCGCGGCGGGCGGTGGGCCGCGGGGGTGTCCTTGGA
TGATGCATCTAAAAGCGGTGACGCGGGCGAGCCCCCGGAGGTAGGGGGGGCTCCGGACCCGCCGGGAGAGGGGGCAG
GGGCACGTCGGCGCCGCGCGCGGGCAGGAGCTGGTGCTGCGCGCGTAGGTTGCTGGCGAACGCGACGACGCGGCGGTT
GATCTCCTGAATCTGGCGCCTCTGCGTGAAGACGACGGGCCCGGTGAGCTTGAACCTGAAAGAGAGTTCGACAGAATCAA
TTTCGGTGTCGTTGACGGCGGCCTGGCGCAAAATCTCCTGCACGTCTCCTGAGTTGTCTTGATAGGCGATCTCGGCCATGA
ACTGCTCGATCTCTTCCTCCTGGAGATCTCCGCGTCCGGCTCGCTCCACGGTGGCGGCGAGGTCGTTGGAAATGCGGGCC
ATGAGCTGCGAGAAGGCGTTGAGGCCTCCCTCGTTCCAGACGCGGCTGTAGACCACGCCCCCTTCGGCATCGCGGGCGC
GCATGACCACCTGCGCGAGATTGAGCTCCACGTGCCGGGCGAAGACGGCGTAGTTTCGCAGGCGCTGAAAGAGGTAGTT
GAGGGTGGTGGCGGTGTGTTCTGCCACGAAGAAGTACATAACCCAGCGTCGCAACGTGGATTCGTTGATATCCCCCAAGG
CCTCAAGGCGCTCCATGGCCTCGTAGAAGTCCACGGCGAAGTTGAAAAACTGGGAGTTGCGCGCCGACACGGTTAACTCC
TCCTCCAGAAGACGGATGAGCTCGGCGACAGTGTCGCGCACCTCGCGCTCAAAGGCTACAGGGGCCTCTTCTTCTTCTTCA
ATCTCCTCTTCCATAAGGGCCTCCCCTTCTTCTTCTTCTGGCGGCGGTGGGGGAGGGGGGACACGGCGGCGACGACGGCG
CACCGGGAGGCGGTCGACAAAGCGCTCGATCATCTCCCCGCGGCGACGGCGCATGGTCTCGGTGACGGCGCGGCCGTTC
TCGCGGGGGCGCAGTTGGAAGACGCCGCCCGTCATGTCCCGGTTATGGGTTGGCGGGGGGCTGCCATGCGGCAGGGATA
CGGCGCTAACGATGCATCTCAACAATTGTTGTGTAGGTACTCCGCCGCCGAGGGACCTGAGCGAGTCCGCATCGACCGGA

TCGGAAAACCTCTCGAGAAAGGCGTCTAACCAGTCACAGTCGCAAGGTAGGCTGAGCACCGTGGCGGGCGGCAGCGGG
CGGCGGTCGGGGTTGTTTCTGGCGGAGGTGCTGCTGATGATGTAATTAAAGTAGGCGGTCTTGAGACGGCGGATGGTCGA
CAGAAGCACCATGTCCTTGGGTCCGGCCTGCTGAATGCGCAGGCGGTCGGCCATGCCCCAGGCTTCGTTTTGACATCGGC
GCAGGTCTTTGTAGTAGTCTTGCATGAGCCTTTCTACCGGCACTTCTTCTTCTCCTTCCTCTTGTCCTGCATCTCTTGCATCTA
TCGCTGCGGCGGCGGCGGAGTTTGGCCGTAGGTGGCGCCCTCTTCCTCCCATGCGTGTGACCCCGAAGCCCCTCATCGGC
TGAAGCAGGGCTAGGTCGGCGACAACGCGCTCGGCTAATATGGCCTGCTGCACCTGCGTGAGGGTAGACTGGAAGTCAT
CCATGTCCACAAAGCGGTGGTATGCGCCCGTGTTGATGGTGTAAGTGCAGTTGGCCATAACGGACCAGTTAACGGTCTGG
TGACCCGGCTGCGAGAGCTCGGTGTACCTGAGACGCGAGTAAGCCCTCGAGTCAAATACGTAGTCGTTGCAAGTCCGCAC
CAGGTACTGGTATCCCACCAAAAAGTGCGGCGGCGGCTGGCGGTAGAGGGGCCAGCGTAGGGTGGCCGGGGCTCCGGG
GGCGAGATCTTCCAACATAAGGCGATGATATCCGTAGATGTACCTGGACATCCAGGTGATGCCGGCGGCGGTGGTGGAG
GCGCGCGGAAAGTCGCGGACGCGGTTCCAGATGTTGCGCAGCGGCAAAAAGTGCTCCATGGTCGGGACGCTCTGGCCGG
TCAGGCGCGCGCAATCGTTGACGCTCTAGACCGTGCAAAAGGAGAGCCTGTAAGCGGGCACTCTTCCGTGGTCTGGTGGA
TAAATTCGCAAGGGTATCATGGCGGACGACCGGGGTTCGAGCCCCGTATCCGGCCGTCCGCCGTGATCCATGCGGTTACC
GCCCGCGTGTCGAACCCAGGTGTGCGACGTCAGACAACGGGGGAGTGCTCCTTTTGGCTTCCTTCCAGGCGCGGCGGCTG
CTGCGCTAGCTTTTTTGGCCACTGGCCGCGCGCAGCGTAAGCGGTTAGGCTGGAAAGCGAAAGCATTAAGTGGCTCGCTC
CCTGTAGCCGGAGGGTTATTTTCCAAGGGTTGAGTCGCGGGACCCCCGGTTCGAGTCTCGGACCGGCCGGACTGCGGCG
AACGGGGGTTTGCCTCCCCGTCATGCAAGACCCCGCTTGCAAATTCCTCCGGAAACAGGGACGAGCCCCTTTTTTGCTTTT
CCCAGATGCATCCGGTGCTGCGGCAGATGCGCCCCCCTCCTCAGCAGCGGCAAGAGCAAGAGCAGCGGCAGACATGCA
GGGCACCCTCCCCTCCTCCTACCGCGTCAGGAGGGGCGACATCCGCGGTTGACGCGGCAGCAGATGGTGATTACGAACC
CCCGCGGCGCCGGGCCCGGCACTACCTGGACTTGGAGGAGGGCGAGGGCCTGGCGCGGCTAGGAGCGCCCTCTCCTGA
GCGGCACCCAAGGGTGCAGCTGAAGCGTGATACGCGTGAGGCGTACGTGCCGCGGCAGAACCTGTTTCGCGACCGCGAG
GGAGAGGAGCCCGAGGAGATGCGGGATCGAAAGTTCCACGCAGGGCGCGAGCTGCGGCATGGCCTGAATCGCGAGCGG
TTGCTGCGCGAGGAGGACTTTGAGCCCGACGCGCGAACCGGGATTAGTCCCGCGCGCGCACACGTGGCGGCCGCCGACC
TGGTAACCGCATACGAGCAGACGGTGAACCAGGAGATTAACTTTCAAAAAAGCTTTAACAACCACGTGCGTACGCTTGTG
GCGCGCGAGGAGGTGGCTATAGGACTGATGCATCTGTGGGACTTTGTAAGCGCGCTGGAGCAAAACCCAAATAGCAAGC
CGCTCATGGCGCAGCTGTTCCTTATAGTGCAGCACAGCAGGGACAACGAGGCATTCAGGGATGCGCTGCTAAACATAGTA
GAGCCCGAGGGCCGCTGGCTGCTCGATTTGATAAACATCCTGCAGAGCATAGTGGTGCAGGAGCGCAGCTTGAGCCTGG
CTGACAAGGTGGCCGCCATCAACTATTCCATGCTTAGCCTGGGCAAGTTTTACGCCCGCAAGATATACCATACCCCTTACG
TTCCCATAGACAAGGAGGTAAAGATCGAGGGGTTCTACATGCGCATGGCGCTGAAGGTGCTTACCTTGAGCGACGACCTG
GGCGTTTATCGCAACGAGCGCATCCACAAGGCCGTGAGCGTGAGCCGGCGGCGCGAGCTCAGCGACCGCGAGCTGATG
CACAGCCTGCAAAGGGCCCTGGCTGGCACGGGCAGCGGCGATAGAGAGGCCGAGTCCTACTTTGACGCGGGCGCTGAC
CTGCGCTGGGCCCCAAGCCGACGCGCCCTGGAGGCAGCTGGGGCCGGACCTGGGCTGGCGGTGGCACCCGCGCGCGCT
GGCAACGTCGGCGGCGTGGAGGAATATGACGAGGACGATGAGTACGAGCCAGAGGACGGCGAGTACTAAGCGGTGATG
TTTCTGATCAGATGATGCAAGACGCAACGGACCCGGCGGTGCGGGCGGCGCTGCAGAGCCAGCCGTCCGGCCTTAACTC
CACGGACGACTGGCGCCAGGTCATGGACCGCATCATGTCGCTGACTGCGCGCAATCCTGACGCGTTCCGGCAGCAGCCG
CAGGCCAACCGGCTCTCCGCAATTCTGGAAGCGGTGGTCCCGGCGCGCGCAAACCCCACGCACGAGAAGGTGCTGGCGA
TCGTAAACGCGCTGGCCGAAAACAGGGCCATCCGGCCCGACGAGGCCGGCCTGGTCTACGACGCGCTGCTTCAGCGCGT
GGCTCGTTACAACAGCGGCAACGTGCAGACCAACCTGGACCGGCTGGTGGGGGATGTGCGCGAGGCCGTGGCGCAGCG
TGAGCGCGCGCAGCAGCAGGGCAACCTGGGCTCCATGGTTGCACTAAACGCCTTCCTGAGTACACAGCCCGCCAACGTG
CCGCGGGGACAGGAGGACTACACCAACTTTGTGAGCGCACTGCGGCTAATGGTGACTGAGACACCGCAAAGTGAGGTGT
ACCAGTCTGGGCCAGACTATTTTTTCCAGACCAGTAGACAAGGCCTGCAGACCGTAAACCTGAGCCAGGCTTTCAAAAACT
TGCAGGGGCTGTGGGGGGTGCGGGCTCCCACAGGCGACCGCGCGACCGTGTCTAGCTTGCTGACGCCCAACTCGCGCCT
GTTGCTGCTGCTAATAGCGCCCTTCACGGACAGTGGCAGCGTGTCCCGGGACACATACCTAGGTCACTTGCTGACACTGTA
CCGCGAGGCCATAGGTCAGGCGCATGTGGACGAGCATACTTTCCAGGAGATTACAAGTGTCAGCCGCGCGCTGGGGCAG
GAGGACACGGGCAGCCTGGAGGCAACCCTAAACTACCTGCTGACCAACCGGCGGCAGAAGATCCCCTCGTTGCACAGTT
TAAACAGCGAGGAGGAGCGCATTTTGCGCTACGTGCAGCAGAGCGTGAGCCTTAACCTGATGCGCGACGGGGTAACGCC
CAGCGTGGCGCTGGACATGACCGCGCGCAACATGGAACCGGGCATGTATGCCTCAAACCGGCCGTTTATCAACCGCCTAA
TGGACTACTTGCATCGCGCGGCCGCCGTGAACCCCGAGTATTTCACCAATGCCATCTTGAACCCGCACTGGCTACCGCCC
CCTGGTTTCTACACCGGGGGATTCGAGGTGCCCGAGGGTAACGATGGATTCCTCTGGGACGACATAGACGACAGCGTGTT

TTCCCCGCAACCGCAGACCCTGCTAGAGTTGCAACAGCGCGAGCAGGCAGAGGCGGCGCTGCGAAAGGAAAGCTTCCGC
AGGCCAAGCAGCTTGTCCGATCTAGGCGCTGCGGCCCCGCGGTCAGATGCTAGTAGCCCATTTCCAAGCTTGATAGGGTC
TCTTACCAGCACTCGCACCACCCGCCCGCGCCTGCTGGGCGAGGAGGAGTACCTAAACAACTCGCTGCTGCAGCCGCAG
CGCGAAAAAAACCTGCCTCCGGCATTTCCCAACAACGGGATAGAGAGCCTAGTGGACAAGATGAGTAGATGGAAGACGT
ACGCGCAGGAGCACAGGGACGTGCCAGGCCCGCGCCCGCCCACCCGTCGTCAAAGGCACGACCGTCAGCGGGGTCTGG
TGTGGGAGGACGATGACTCGGCAGACGACAGCAGCGTCCTGGATTTGGGAGGGAGTGGCAACCCGTTTGCGCACCTTCG
CCCCAGGCTGGGGAGAATGTTTTAAAAAAAAAAAAAAGCATGATGCAAAATAAAAAACTCACCAAGGCCATGGCACCGAG
CGTTGGTTTTCTTGTATTCCCCTTAGTATGCGGCGCGCGGCGATGTATGAGGAAGGTCCTCCTCCCTCCTACGAGAGTGTG
GTGAGCGCGGCGCCAGTGGCGGCGGCGCTGGGTTCTCCCTTCGATGCTCCCCTGGACCCGCCGTTTGTGCCTCCGCGGTA
CCTGCGGCCTACCGGGGGGAGAAACAGCATCCGTTACTCTGAGTTGGCACCCCTATTCGACACCACCCGTGTGTACCTGG
TGGACAACAAGTCAACGGATGTGGCATCCCTGAACTACCAGAACGACCACAGCAACTTTCTGACCACGGTCATTCAAAAC
AATGACTACAGCCCGGGGGAGGCAAGCACACAGACCATCAATCTTGACGACCGGTCGCACTGGGGCGGCGACCTGAAAA
CCATCCTGCATACCAACATGCCAAATGTGAACGAGTTCATGTTTACCAATAAGTTTAAGGCGCGGGTGATGGTGTCGCGCT
TGCCTACTAAGGACAATCAGGTGGAGCTGAAATACGAGTGGGTGGAGTTCACGCTGCCCGAGGGCAACTACTCCGAGAC
CATGACCATAGACCTTATGAACAACGCGATCGTGGAGCACTACTTGAAAGTGGGCAGACAGAACGGGGTTCTGGAAAGC
GACATCGGGGTAAAGTTTGACACCCGCAACTTCAGACTGGGGTTTGACCCCGTCACTGGTCTTGTCATGCCTGGGGTATAT
ACAAACGAAGCCTTCCATCCAGACATCATTTTGCTGCCAGGATGCGGGGTGGACTTCACCCACAGCCGCCTGAGCAACTT
GTTGGGCATCCGCAAGCGGCAACCCTTCCAGGAGGGCTTTAGGATCACCTACGATGATCTGGAGGGTGGTAACATTCCCG
CACTGTTGGATGTGGACGCCTACCAGGCGAGCTTGAAAGATGACACCGAACAGGGCGGGGGTGGCGCAGGCGGCAGCA
ACAGCAGTGGCAGCGGCGCGGAAGAGAACTCCAACGCGGCAGCCGCGGCAATGCAGCCGGTGGAGGACATGAACGATC
ATGCCATTCGCGGCGACACCTTTGCCACACGGGCTGAGGAGAAGCGCGCTGAGGCCGAAGCAGCGGCCGAAGCTGCCG
CCCCCGCTGCGCAACCCGAGGTCGAGAAGCCTCAGAAGAAACCGGTGATCAAACCCCTGACAGAGGACAGCAAGAAAC
GCAGTTACAACCTAATAAGCAATGACAGCACCTTCACCCAGTACCGCAGCTGGTACCTTGCATACAACTACGGCGACCCT
CAGACCGGAATCCGCTCATGGACCCTGCTTTGCACTCCTGACGTAACCTGCGGCTCGGAGCAGGTCTACTGGTCGTTGCC
AGACATGATGCAAGACCCCGTGACCTTCCGCTCCACGCGCCAGATCAGCAACTTTCCGGTGGTGGGCGCCGAGCTGTTGC
CCGTGCACTCCAAGAGCTTCTACAACGACCAGGCCGTCTACTCCCAACTCATCCGCCAGTTTACCTCTCTGACCCACGTGT
TCAATCGCTTTCCCGAGAACCAGATTTTGGCGCGCCCGCCAGCCCCCACCATCACCACCGTCAGTGAAAACGTTCCTGCTC
TCACAGATCACGGGACGCTACCGCTGCGCAACAGCATCGGAGGAGTCCAGCGAGTGACCATTACTGACGCCAGACGCCG
CACCTGCCCCTACGTTTACAAGGCCCTGGGCATAGTCTCGCCGCGCGTCCTATCGAGCCGCACTTTTTGAGCAAGCATGTC
CATCCTTATATCGCCCAGCAATAACACAGGCTGGGGCCTGCGCTTCCCAAGCAAGATGTTTGGCGGGGCCAAGAAGCGCT
CCGACCAACACCCAGTGCGCGTGCGCGGGCACTACCGCGCGCCCTGGGGCGCGCACAAACGCGGCCGCACTGGGCGCA
CCACCGTCGATGACGCCATCGACGCGGTGGTGGAGGAGGCGCGCAACTACACGCCCACGCCGCCACCAGTGTCCACAGT
GGACGCGGCCATTCAGACCGTGGTGCGCGGAGCCCGGCGCTATGCTAAAATGAAGAGACGGCGGAGGCGCGTAGCACG
TCGCCACCGCCGCCGACCCGGCACTGCCGCCCAACGCGCGGCGGCGGCCCTGCTTAACCGCGCACGTCGCACCGGCCG
ACGGGCGGCCATGCGGGCCGCTCGAAGGCTGGCCGCGGGTATTGTCACTGTGCCCCCCAGGTCCAGGCGACGAGCGGC
CGCCGCAGCAGCCGCGGCCATTAGTGCTATGACTCAGGGTCGCAGGGGCAACGTGTATTGGGTGCGCGACTCGGTTAGC
GGCCTGCGCGTGCCCGTGCGCACCCGCCCCCCGCGCAACTAGATTGCAAGAAAAAACTACTTAGACTCGTACTGTTGTAT
GTATCCAGCGGCGGCGGCGCGCAACGAAGCTATGTCCAAGCGCAAAATCAAAGAAGAGATGCTCCAGGTCATCGCGCCG
GAGATCTATGGCCCCCCGAAGAAGGAAGAGCAGGATTACAAGCCCCGAAAGCTAAAGCGGGTCAAAAAGAAAAAGAAA
GATGATGATGATGAACTTGACGACGAGGTGGAACTGCTGCACGCTACCGCGCCCAGGCGACGGGTACAGTGGAAAGGTC
GACGCGTAAAACGTGTTTTGCGACCCGGCACCACCGTAGTCTTTACGCCCGGTGAGCGCTCCACCCGCACCTACAAGCGC
GTGTATGATGAGGTGTACGGCGACGAGGACCTGCTTGAGCAGGCCAACGAGCGCCTCGGGGAGTTTGCCTACGGAAAGC
GGCATAAGGACATGCTGGCGTTGCCGCTGGACGAGGGCAACCCAACACCTAGCCTAAAGCCCGTAACACTGCAGCAGGT
GCTGCCCGCGCTTGCACCGTCCGAAGAAAAGCGCGGCCTAAAGCGCGAGTCTGGTGACTTGGCACCCACCGTGCAGCTG
ATGGTACCCAAGCGCCAGCGACTGGAAGATGTCTTGGAAAAAATGACCGTGGAACCTGGGCTGGAGCCCGAGGTCCGCG
TGCGGCCAATCAAGCAGGTGGCGCCGGGACTGGGCGTGCAGACCGTGGACGTTCAGATACCCACTACCAGTAGCACCAG
TATTGCCACCGCCACAGAGGGCATGGAGACACAAACGTCCCCGGTTGCCTCAGCGGTGGCGGATGCCGCGGTGCAGGCG
GTCGCTGCGGCCGCGTCCAAGACCTCTACGGAGGTGCAAACGGACCCGTGGATGTTTCGCGTTTCAGCCCCCCGGCGCCC
GCGCCGTTCGAGGAAGTACGGCGCCGCCAGCGCGCTACTGCCCGAATATGCCCTACATCCTTCCATTGCGCCTACCCCCG

GCTATCGTGGCTACACCTACCGCCCCAGAAGACGAGCAACTACCCGACGCCGAACCACCACTGGAACCCGCCGCCGCCG
TCGCCGTCGCCAGCCCGTGCTGGCCCCGATTTCCGTGCGCAGGGTGGCTCGCGAAGGAGGCAGGACCCTGGTGCTGCCA
ACAGCGCGCTACCACCCCAGCATCGTTTAAAAGCCGGTCTTTGTGGTTCTTGCAGATATGGCCCTCACCTGCCGCCTCCGT
TTCCCGGTGCCGGGATTCCGAGGAAGAATGCACCGTAGGAGGGGCATGGCCGGCCACGGCCTGACGGGCGGCATGCGT
CGTGCGCACCACCGGCGGCGGCGCGCGTCGCACCGTCGCATGCGCGGCGGTATCCTGCCCCTCCTTATTCCACTGATCGC
CGCGGCGATTGGCGCCGTGCCCGGAATTGCATCCGTGGCCTTGCAGGCGCAGAGACACTGATTAAAAACAAGTTGCATGT
GGAAAAATCAAAATAAAAAGTCTGGACTCTCACGCTCGCTTGGTCCTGTAACTATTTTGTAGAATGGAAGACATCAACTTT
GCGTCTCTGGCCCCGCGACACGGCTCGCGCCCGTTCATGGGAAACTGGCAAGATATCGGCACCAGCAATATGAGCGGTG
GCGCCTTCAGCTGGGGCTCGCTGTGGAGCGGCATTAAAAATTTCGGTTCCACCGTTAAGAACTATGGCAGCAAGGCCTGG
AACAGCAGCACAGGCCAGATGCTGAGGGATAAGTTGAAAGAGCAAAATTTCCAACAAAAGGTGGTAGATGGCCTGGCCT
CTGGCATTAGCGGGGTGGTGGACCTGGCCAACCAGGCAGTGCAAAATAAGATTAACAGTAAGCTTGATCCCCGCCCTCCC
GTAGAGGAGCCTCCACCGGCCGTGGAGACAGTGTCTCCAGAGGGGCGTGGCGAAAAGCGTCCGCGCCCCGACAGGGAA
GAAACTCTGGTGACGCAAATAGACGAGCCTCCCTCGTACGAGGAGGCACTAAAGCAAGGCCTGCCCACCACCCGTCCCA
TCGCGCCCATGGCTACCGGAGTGCTGGGCCAGCACACACCCGTAACGCTGGACCTGCCTCCCCCCGCCGACACCCAGCA
GAAACCTGTGCTGCCAGGCCCGACCGCCGTTGTTGTAACCCGTCCTAGCCGCGCGTCCCTGCGCCGCGCCGCCAGCGGTC
CGCGATCGTTGCGGCCCGTAGCCAGTGGCAACTGGCAAAGCACACTGAACAGCATCGTGGGTCTGGGGGTGCAATCCCT
GAAGCGCCGACGATGCTTCTGATAGCTAACGTGTCGTATGTGTGTCATGTATGCGTCCATGTCGCCGCCAGAGGAGCTGCT
GAGCCGCCGCGCGCCCGCTTTCCAAGATGGCTACCCCTTCGATGATGCCGCAGTGGTCTTACATGCACATCTCGGGCCAG
GACGCCTCGGAGTACCTGAGCCCCGGGCTGGTGCAGTTTGCCCGCGCCACCGAGACGTACTTCAGCCTGAATAACAAGTT
TAGAAACCCCACGGTGGCGCCTACGCACGACGTGACCACAGACCGGTCCCAGCGTTTGACGCTGCGGTTCATCCCTGTGG
ACCGTGAGGATACTGCGTACTCGTACAAGGCGCGGTTCACCCTAGCTGTGGGTGATAACCGTGTGCTGGACATGGCTTCC
ACGTACTTTGACATCCGCGGCGTGCTGGACAGGGGCCCTACTTTTAAGCCCTACTCTGGCACTGCCTACAACGCCCTGGCT
CCCAAGGGTGCCCCAAATCCTTGCGAATGGGATGAAGCTGCTACTGCTCTTGAAATAAACCTAGAAGAAGAGGACGATGA
CAACGAAGACGAAGTAGACGAGCAAGCTGAGCAGCAAAAAACTCACGTATTTGGGCAGGCGCCTTATTCTGGTATAAATA
TTACAAAGGAGGGTATTCAAATAGGTGTCGAAGGTCAAACACCTAAATATGCCGATAAAACATTTCAACCTGAACCTCAAA
TAGGAGAATCTCAGTGGTACGAAACAGAAATTAATCATGCAGCTGGGAGAGTCCTAAAAAAGACTACCCCAATGAAACCA
TGTTACGGTTCATATGCAAAACCCACAAATGAAAATGGAGGGCAAGGCATTCTTGTAAAGCAACAAAATGGAAAGCTAGA
AAGTCAAGTGGAAATGCAATTTTTCTCAACTACTGAGGCAGCCGCAGGCAATGGTGATAACTTGACTCCTAAAGTGGTATT
GTACAGTGAAGATGTAGATATAGAAACCCCAGACACTCATATTTCTTACATGCCCACTATTAAGGAAGGTAACTCACGAGA
ACTAATGGGCCAACAATCTATGCCCAACAGGCCTAATTACATTGCTTTTAGGGACAATTTTATTGGTCTAATGTATTACAAC
AGCACGGGTAATATGGGTGTTCTGGCGGGCCAAGCATCGCAGTTGAATGCTGTTGTAGATTTGCAAGACAGAAACACAGA
GCTTTCATACCAGCTTTTGCTTGATTCCATTGGTGATAGAACCAGGTACTTTTCTATGTGGAATCAGGCTGTTGACAGCTATG
ATCCAGATGTTAGAATTATTGAAAATCATGGAACTGAAGATGAACTTCCAAATTACTGCTTTCCACTGGGAGGTGTGATTAA
TACAGAGACTCTTACCAAGGTAAAACCTAAAACAGGTCAGGAAAATGGATGGGAAAAAGATGCTACAGAATTTTCAGATA
AAAATGAAATAAGAGTTGGAAATAATTTTGCCATGGAAATCAATCTAAATGCCAACCTGTGGAGAAATTTCCTGTACTCCA
ACATAGCGCTGTATTTGCCCGACAAGCTAAAGTACAGTCCTTCCAACGTAAAAATTTCTGATAACCCAAACACCTACGACT
ACATGAACAAGCGAGTGGTGGCTCCCGGGCTAGTGGACTGCTACATTAACCTTGGAGCACGCTGGTCCCTTGACTATATG
GACAACGTCAACCCATTTAACCACCACCGCAATGCTGGCCTGCGCTACCGCTCAATGTTGCTGGGCAATGGTCGCTATGTG
CCCTTCCACATCCAGGTGCCTCAGAAGTTCTTTGCCATTAAAAACCTCCTTCTCCTGCCGGGCTCATACACCTACGAGTGGA
ACTTCAGGAAGGATGTTAACATGGTTCTGCAGAGCTCCCTAGGAAATGACCTAAGGGTTGACGGAGCCAGCATTAAGTTT
GATAGCATTTGCCTTTACGCCACCTTCTTCCCCATGGCCCACAACACCGCCTCCACGCTTGAGGCCATGCTTAGAAACGAC
ACCAACGACCAGTCCTTTAACGACTATCTCTCCGCCGCCAACATGCTCTACCCTATACCCGCCAACGCTACCAACGTGCCC
ATATCCATCCCCTCCCGCAACTGGGCGGCTTTCCGCGGCTGGGCCTTCACGCGCCTTAAGACTAAGGAAACCCCATCACT
GGGCTCGGGCTACGACCCTTATTACACCTACTCTGGCTCTATACCCTACCTAGATGGAACCTTTTACCTCAACCACACCTTT
AAGAAGGTGGCCATTACCTTTGACTCTTCTGTCAGCTGGCCTGGCAATGACCGCCTGCTTACCCCCAACGAGTTTGAAATT
AAGCGCTCAGTTGACGGGGAGGGTTACAACGTTGCCCAGTGTAACATGACCAAAGACTGGTTCCTGGTACAAATGCTAGC
TAACTATAACATTGGCTACCAGGGCTTCTATATCCCAGAGAGCTACAAGGACCGCATGTACTCCTTCTTTAGAAACTTCCAG
CCCATGAGCCGTCAGGTGGTGGATGATACTAAATACAAGGACTACCAACAGGTGGGCATCCTACACCAACACAACAACTC
TGGATTTGTTGGCTACCTTGCCCCCACCATGCGCGAAGGACAGGCCTACCCTGCTAACTTCCCCTATCCGCTTATAGGCAA

GACCGCAGTTGACAGCATTACCCAGAAAAAGTTTCTTTGCGATCGCACCCTTTGGCGCATCCCATTCTCCAGTAACTTTATG
TCCATGGGCGCACTCACAGACCTGGGCCAAAACCTTCTCTACGCCAACTCCGCCCACGCGCTAGACATGACTTTTGAGGT
GGATCCCATGGACGAGCCCACCCTTCTTTATGTTTTGTTTGAAGTCTTTGACGTGGTCCGTGTGCACCAGCCGCACCGCGG
CGTCATCGAAACCGTGTACCTGCGCACGCCCTTCTCGGCCGGCAACGCCACAACATAAAGAAGCAAGCAACATCAACAA
CAGCTGCCGCCATGGGCTCCAGTGAGCAGGAACTGAAAGCCATTGTCAAAGATCTTGGTTGTGGGCCATATTTTTTGGGCA
CCTATGACAAGCGCTTTCCAGGCTTTGTTTCTCCACACAAGCTCGCCTGCGCCATAGTCAATACGGCCGGTCGCGAGACTG
GGGGCGTACACTGGATGGCCTTTGCCTGGAACCCGCACTCAAAAACATGCTACCTCTTTGAGCCCTTTGGCTTTTCTGACC
AGCGACTCAAGCAGGTTTACCAGTTTGAGTACGAGTCACTCCTGCGCCGTAGCGCCATTGCTTCTTCCCCCGACCGCTGTA
TAACGCTGGAAAAGTCCACCCAAAGCGTACAGGGGCCCAACTCGGCCGCCTGTGGACTATTCTGCTGCATGTTTCTCCAC
GCCTTTGCCAACTGGCCCCAAACTCCCATGGATCACAACCCCACCATGAACCTTATTACCGGGGTACCCAACTCCATGCTC
AACAGTCCCCAGGTACAGCCCACCCTGCGTCGCAACCAGGAACAGCTCTACAGCTTCCTGGAGCGCCACTCGCCCTACTT
CCGCAGCCACAGTGCGCAGATTAGGAGCGCCACTTCTTTTTGTCACTTGAAAAACATGTAAAAATAATGTACTAGAGACAC
TTTCAATAAAGGCAAATGCTTTTATTTGTACACTCTCGGGTGATTATTTACCCCCACCCTTGCCGTCTGCGCCGTTTAAAAAT
CAAAGGGGTTCTGCCGCGCATCGCTATGCGCCACTGGCAGGGACACGTTGCGATACTGGTGTTTAGTGCTCCACTTAAACT
CAGGCACAACCATCCGCGGCAGCTCGGTGAAGTTTTCACTCCACAGGCTGCGCACCATCACCAACGCGTTTAGCAGGTCG
GGCGCCGATATCTTGAAGTCGCAGTTGGGGCCTCCGCCCTGCGCGCGCGAGTTGCGATACACAGGGTTGCAGCACTGGA
ACACTATCAGCGCCGGGTGGTGCACGCTGGCCAGCACGCTCTTGTCGGAGATCAGATCCGCGTCCAGGTCCTCCGCGTTG
CTCAGGGCGAACGGAGTCAACTTTGGTAGCTGCCTTCCCAAAAAGGGCGCGTGCCCAGGCTTTGAGTTGCACTCGCACCG
TAGTGGCATCAAAAGGTGACCGTGCCCGGTCTGGGCGTTAGGATACAGCGCCTGCATAAAAGCCTTGATCTGCTTAAAAG
CCACCTGAGCCTTTGCGCCTTCAGAGAAGAACATGCCGCAAGACTTGCCGGAAAACTGATTGGCCGGACAGGCCGCGTC
GTGCACGCAGCACCTTGCGTCGGTGTTGGAGATCTGCACCACATTTCGGCCCCACCGGTTCTTCACGATCTTGGCCTTGCT
AGACTGCTCCTTCAGCGCGCGCTGCCCGTTTTCGCTCGTCACATCCATTTCAATCACGTGCTCCTTATTTATCATAATGCTTC
CGTGTAGACACTTAAGCTCGCCTTCGATCTCAGCGCAGCGGTGCAGCCACAACGCGCAGCCCGTGGGCTCGTGATGCTTG
TAGGTCACCTCTGCAAACGACTGCAGGTACGCCTGCAGGAATCGCCCCATCATCGTCACAAAGGTCTTGTTGCTGGTGAA
GGTCAGCTGCAACCCGCGGTGCTCCTCGTTCAGCCAGGTCTTGCATACGGCCGCCAGAGCTTCCACTTGGTCAGGCAGTA
GTTTGAAGTTCGCCTTTAGATCGTTATCCACGTGGTACTTGTCCATCAGCGCGCGCGCAGCCTCCATGCCCTTCTCCCACGC
AGACACGATCGGCACACTCAGCGGGTTCATCACCGTAATTTCACTTTCCGCTTCGCTGGGCTCTTCCTCTTCCTCTTGCGTC
CGCATACCACGCGCCACTGGGTCGTCTTCATTCAGCCGCCGCACTGTGCGCTTACCTCCTTTGCCATGCTTGATTAGCACC
GGTGGGTTGCTGAAACCCACCATTTGTAGCGCCACATCTTCTCTTTCTTCCTCGCTGTCCACGATTACCTCTGGTGATGGCG
GGCGCTCGGGCTTGGGAGAAGGGCGCTTCTTTTTCTTCTTGGGCGCAATGGCCAAATCCGCCGCCGAGGTCGATGGCCGC
GGGCTGGGTGTGCGCGGCACCAGCGCGTCTTGTGATGAGTCTTCCTCGTCCTCGGACTCGATACGCCGCCTCATCCGCTTT
TTTGGGGGCGCCCGGGGAGGCGGCGGCGACGGGGACGGGGACGACACGTCCTCCATGGTTGGGGGACGTCGCGCCGCA
CCGCGTCCGCGCTCGGGGGTGGTTTCGCGCTGCTCCTCTTCCCGACTGGCCATTTCCTTCTCCTATAGGCAGAAAAAGATC
ATGGAGTCAGTCGAGAAGAAGGACAGCCTAACCGCCCCCTCTGAGTTCGCCACCACCGCCTCCACCGATGCCGCCAACG
CGCCTACCACCTTCCCCGTCGAGGCACCCCCGCTTGAGGAGGAGGAAGTGATTATCGAGCAGGACCCAGGTTTTGTAAGC
GAAGACGACGAGGACCGCTCAGTACCAACAGAGGATAAAAAGCAAGACCAGGACAACGCAGAGGCAAACGAGGAACA
AGTCGGGCGGGGGGACGAAAGGCATGGCGACTACCTAGATGTGGGAGACGACGTGCTGTTGAAGCATCTGCAGCGCCA
GTGCGCCATTATCTGCGACGCGTTGCAAGAGCGCAGCGATGTGCCCCTCGCCATAGCGGATGTCAGCCTTGCCTACGAAC
GCCACCTATTCTCACCGCGCGTACCCCCCAAACGCCAAGAAAACGGCACATGCGAGCCCAACCCGCGCCTCAACTTCTAC
CCCGTATTTGCCGTGCCAGAGGTGCTTGCCACCTATCACATCTTTTTCCAAAACTGCAAGATACCCCTATCCTGCCGTGCCA
ACCGCAGCCGAGCGGACAAGCAGCTGGCCTTGCGGCAGGGCGCTGTCATACCTGATATCGCCTCGCTCAACGAAGTGCC
AAAAATCTTTGAGGGTCTTGGACGCGACGAGAAGCGCGCGGCAAACGCTCTGCAACAGGAAAACAGCGAAAATGAAAGT
CACTCTGGAGTGTTGGTGGAACTCGAGGGTGACAACGCGCGCCTAGCCGTACTAAAACGCAGCATCGAGGTCACCCACTT
TGCCTACCCGGCACTTAACCTACCCCCCAAGGTCATGAGCACAGTCATGAGTGAGCTGATCGTGCGCCGTGCGCAGCCCC
TGGAGAGGGATGCAAATTTGCAAGAACAAACAGAGGAGGGCCTACCCGCAGTTGGCGACGAGCAGCTAGCGCGCTGGCT
TCAAACGCGCGAGCCTGCCGACTTGGAGGAGCGACGCAAACTAATGATGGCCGCAGTGCTCGTTACCGTGGAGCTTGAGT
GCATGCAGCGGTTCTTTGCTGACCCGGAGATGCAGCGCAAGCTAGAGGAAACATTGCACTACACCTTTCGACAGGGCTAC
GTACGCCAGGCCTGCAAGATCTCCAACGTGGAGCTCTGCAACCTGGTCTCCTACCTTGGAATTTTGCACGAAAACCGCCTT
GGGCAAAACGTGCTTCATTCCACGCTCAAGGGCGAGGCGCGCCGCGACTACGTCCGCGACTGCGTTTACTTATTTCTATGC

TACACCTGGCAGACGGCCATGGGCGTTTGGCAGCAGTGCTTGGAGGAGTGCAACCTCAAGGAGCTGCAGAAACTGCTAA
AGCAAAACTTGAAGGACCTATGGACGGCCTTCAACGAGCGCTCCGTGGCCGCGCACCTGGCGGACATCATTTTCCCCGAA
CGCCTGCTTAAAACCCTGCAACAGGGTCTGCCAGACTTCACCAGTCAAAGCATGTTGCAGAACTTTAGGAACTTTATCCTA
GAGCGCTCAGGAATCTTGCCCGCCACCTGCTGTGCACTTCCTAGCGACTTTGTGCCCATTAAGTACCGCGAATGCCCTCCG
CCGCTTTGGGGCCACTGCTACCTTCTGCAGCTAGCCAACTACCTTGCCTACCACTCTGACATAATGGAAGACGTGAGCGGT
GACGGTCTACTGGAGTGTCACTGTCGCTGCAACCTATGCACCCCGCACCGCTCCCTGGTTTGCAATTCGCAGCTGCTTAAC
GAAAGTCAAATTATCGGTACCTTTGAGCTGCAGGGTCCCTCGCCTGACGAAAAGTCCGCGGCTCCGGGGTTGAAACTCAC
TCCGGGGCTGTGGACGTCGGCTTACCTTCGCAAATTTGTACCTGAGGACTACCACGCCCACGAGATTAGGTTCTACGAAGA
CCAATCCCGCCCGCCTAATGCGGAGCTTACCGCCTGCGTCATTACCCAGGGCCACATTCTTGGCCAATTGCAAGCCATCAA
CAAAGCCCGCCAAGAGTTTCTGCTACGAAAGGGACGGGGGGTTTACTTGGACCCCCAGTCCGGCGAGGAGCTCAACCCA
ATCCCCCCGCCGCCGCAGCCCTATCAGCAGCAGCCGCGGGCCCTTGCTTCCCAGGATGGCACCCAAAAAGAAGCTGCAG
CTGCCGCCGCCACCCACGGACGAGGAGGAATACTGGGACAGTCAGGCAGAGGAGGTTTTGGACGAGGAGGAGGAGGAC
ATGATGGAAGACTGGGAGAGCCTAGACGAGGAAGCTTCCGAGGTCGAAGAGGTGTCAGACGAAACACCGTCACCCTCGG
TCGCATTCCCCTCGCCGGCGCCCCAGAAATCGGCAACCGGTTCCAGCATGGCTACAACCTCCGCTCCTCAGGCGCCGCCG
GCACTGCCCGTTCGCCGACCCAACCGTAGATGGGACACCACTGGAACCAGGGCCGGTAAGTCCAAGCAGCCGCCGCCGT
TAGCCCAAGAGCAACAACAGCGCCAAGGCTACCGCTCATGGCGCGGGCACAAGAACGCCATAGTTGCTTGCTTGCAAGA
CTGTGGGGGCAACATCTCCTTCGCCCGCCGCTTTCTTCTCTACCATCACGGCGTGGCCTTCCCCCGTAACATCCTGCATTAC
TACCGTCATCTCTACAGCCCATACTGCACCGGCGGCAGCGGCAGCAACAGCAGCGGCCACACAGAAGCAAAGGCGACCG
GATAGCAAGACTCTGACAAAGCCCAAGAAATCCACAGCGGCGGCAGCAGCAGGAGGAGGAGCGCTGCGTCTGGCGCCC
AACGAACCCGTATCGACCCGCGAGCTTAGAAACAGGATTTTTCCCACTCTGTATGCTATATTTCAACAGAGCAGGGGCCAA
GAACAAGAGCTGAAAATAAAAAACAGGTCTCTGCGATCCCTCACCCGCAGCTGCCTGTATCACAAAAGCGAAGATCAGCT
TCGGCGCACGCTGGAAGACGCGGAGGCTCTCTTCAGTAAATACTGCGCGCTGACTCTTAAGGACTAGTTTCGCGCCCTTTC
TCAAATTTAAGCGCGAAAACTACGTCATCTCCAGCGGCCACACCCGGCGCCAGCACCTGTTGTCAGCGCCATTATGAGCA
AGGAAATTCCCACGCCCTACATGTGGAGTTACCAGCCACAAATGGGACTTGCGGCTGGAGCTGCCCAAGACTACTCAACC
CGAATAAACTACATGAGCGCGGGACCCCACATGATATCCCGGGTCAACGGAATACGCGCCCACCGAAACCGAATTCTCCT
GGAACAGGCGGCTATTACCACCACACCTCGTAATAACCTTAATCCCCGTAGTTGGCCCGCTGCCCTGGTGTACCAGGAAA
GTCCCGCTCCCACCACTGTGGTACTTCCCAGAGACGCCCAGGCCGAAGTTCAGATGACTAACTCAGGGGCGCAGCTTGCG
GGCGGCTTTCGTCACAGGGTGCGGTCGCCCGGGCAGGGTATAACTCACCTGACAATCAGAGGGCGAGGTATTCAGCTCA
ACGACGAGTCGGTGAGCTCCTCGCTTGGTCTCCGTCCGGACGGGACATTTCAGATCGGCGGCGCCGGCCGCTCTTCATTC
ACGCCTCGTCAGGCAATCCTAACTCTGCAGACCTCGTCCTCTGAGCCGCGCTCTGGAGGCATTGGAACTCTGCAATTTATT
GAGGAGTTTGTGCCATCGGTCTACTTTAACCCCTTCTCGGGACCTCCCGGCCACTATCCGGATCAATTTATTCCTAACTTTG
ACGCGGTAAAGGACTCGGCGGACGGCTACGACTGAATGTTAAGTGGAGAGGCAGAGCAACTGCGCCTGAAACACCTGGT
CCACTGTCGCCGCCACAAGTGCTTTGCCCGCGACTCCGGTGAGTTTTGCTACTTTGAATTGCCCGAGGATCATATCGAGGG
CCCGGCGCACGGCGTCCGGCTTACCGCCCAGGGAGAGCTTGCCCGTAGCCTGATTCGGGAGTTTACCCAGCGCCCCCTG
CTAGTTGAGCGGGACAGGGGACCCTGTGTTCTCACTGTGATTTGCAACTGTCCTAACCCTGGATTACATCAAGATCTTTGTT
GCCATCTCTGTGCTGAGTATAATAAATACAGAAATTAAAATATACTGGGGCTCCTATCGCCATCCTGTAAACGCCACCGTCT
TCACCCGCCCAAGCAAACCAAGGCGAACCTTACCTGGTACTTTTAACATCTCTCCCTCTGTGATTTACAACAGTTTCAACCC
AGACGGAGTGAGTCTACGAGAGAACCTCTCCGAGCTCAGCTACTCCATCAGAAAAAACACCACCCTCCTTACCTGCCGGG
AACGTACGAGTGCGTCACCGGCCGCTGCACCACACCTACCGCCTGACCGTAAACCAGACTTTTTCCGGACAGACCTCAAT
AACTCTGTTTACCAGAACAGGAGGTGAGCTTAGAAAACCCTTAGGGTATTAGGCCAAAGGCGCAGCTACTGTGGGGTTTA
TGAACAATTCAAGCAACTCTACGGGCTATTCTAATTCAGGTTTCTCTAGAAATGGACGGAATTATTACAGAGCAGCGCCTG
CTAGAAAGACGCAGGGCAGCGGCCGAGCAACAGCGCATGAATCAAGAGCTCCAAGACATGGTTAACTTGCACCAGTGCA
AAAGGGGTATCTTTTGTCTGGTAAAGCAGGCCAAAGTCACCTACGACAGTAATACCACCGGACACCGCCTTAGCTACAAG
TTGCCAACCAAGCGTCAGAAATTGGTGGTCATGGTGGGAGAAAAGCCCATTACCATAACTCAGCACTCGGTAGAAACCGA
AGGCTGCATTCACTCACCTTGTCAAGGACCTGAGGATCTCTGCACCCTTATTAAGACCCTGTGCGGTCTCAAAGATCTTATT
CCCTTTAACTAATAAAAAAAAATAATAAAGCATCACTTACTTAAAATCAGTTAGCAAATTTCTGTCCAGTTTATTCAGCAGC
ACCTCCTTGCCCTCCTCCCAGCTCTGGTATTGCAGCTTCCTCCTGGCTGCAAACTTTCTCCACAATCTAAATGGAATGTCAG
TTTCCTCCTGTTCCTGTCCATCCGCACCCACTATCTTCATGTTGTTGCAGATGAAGCGCGCAAGACCGTCTGAAGATACCTT
CAACCCCGTGTATCCATATGACACGGAAACCGGTCCTCCAACTGTGCCTTTTCTTACTCCTCCCTTTGTATCCCCCAATGGG

TTTCAAGAGAGTCCCCCTGGGGTACTCTCTTTGCGCCTATCCGAACCTCTAGTTACCTCCAATGGCATGCTTGCGCTCAAAA
TGGGCAACGGCCTCTCTCTGGACGAGGCCGGCAACCTTACCTCCCAAAATGTAACCACTGTGAGCCCACCTCTCAAAAAA
ACCAAGTCAAACATAAACCTGGAAATATCTGCACCCCTCACAGTTACCTCAGAAGCCCTAACTGTGGCTGCCGCCGCACCT
CTAATGGTCGCGGGCAACACACTCACCATGCAATCACAGGCCCCGCTAACCGTGCACGACTCCAAACTTAGCATTGCCAC
CCAAGGACCCCTCACAGTGTCAGAAGGAAAGCTAGCCCTGCAAACATCAGGCCCCCTCACCACCACCGATAGCAGTACC
CTTACTATCACTGCCTCACCCCCTCTAACTACTGCCACTGGTAGCTTGGGCATTGACTTGAAAGAGCCCATTTATACACAAA
ATGGAAAACTAGGACTAAAGTACGGGGCTCCTTTGCATGTAACAGACGACCTAAACACTTTGACCGTAGCAACTGGTCCA
GGTGTGACTATTAATAATACTTCCTTGCAAACTAAAGTTACTGGAGCCTTGGGTTTTGATTCACAAGGCAATATGCAACTTA
ATGTAGCAGGAGGACTAAGGATTGATTCTCAAAACAGACGCCTTATACTTGATGTTAGTTATCCGTTTGATGCTCAAAACCA
ACTAAATCTAAGACTAGGACAGGGCCCTCTTTTTATAAACTCAGCCCACAACTTGGATATTAACTACAACAAAGGCCTTTAC
TTGTTTACAGCTTCAAACAATTCCAAAAAGCTTGAGGTTAACCTAAGCACTGCCAAGGGGTTGATGTTTGACGCTACAGCC
ATAGCCATTAATGCAGGAGATGGGCTTGAATTTGGTTCACCTAATGCACCAAACACAAATCCCCTCAAAACAAAAATTGGC
CATGGCCTAGAATTTGATTCAAACAAGGCTATGGTTCCTAAACTAGGAACTGGCCTTAGTTTTGACAGCACAGGTGCCATT
ACAGTAGGAAACAAAAATAATGATAAGCTAACTTTGTGGACCACACCAGCTCCATCTCCTAACTGTAGACTAAATGCAGAG
AAAGATGCTAAACTCACTTTGGTCTTAACAAAATGTGGCAGTCAAATACTTGCTACAGTTTCAGTTTTGGCTGTTAAAGGCA
GTTTGGCTCCAATATCTGGAACAGTTCAAAGTGCTCATCTTATTATAAGATTTGACGAAAATGGAGTGCTACTAAACAATTC
CTTCCTGGACCCAGAATATTGGAACTTTAGAAATGGAGATCTTACTGAAGGCACAGCCTATACAAACGCTGTTGGATTTAT
GCCTAACCTATCAGCTTATCCAAAATCTCACGGTAAAACTGCCAAAAGTAACATTGTCAGTCAAGTTTACTTAAACGGAGA
CAAAACTAAACCTGTAACACTAACCATTACACTAAACGGTACACAGGAAACAGGAGACACAACTCCAAGTGCATACTCTA
TGTCATTTTCATGGGACTGGTCTGGCCACAACTACATTAATGAAATATTTGCCACATCCTCTTACACTTTTTCATACATTGCC
CAAGAATAAAGAATCGTTTGTGTTATGTTTCAACGTGTTTATTTTTCAATTGCAGAAAATTTCGAATCATTTTTCATTCAGTAG
TATAGCCCCACCACCACATAGCTTATACAGATCACCGTACCTTAATCAAACTCACAGAACCCTAGTATTCAACCTGCCACCT
CCCTCCCAACACACAGAGTACACAGTCCTTTCTCCCCGGCTGGCCTTAAAAAGCATCATATCATGGGTAACAGACATATTC
TTAGGTGTTATATTCCACACGGTTTCCTGTCGAGCCAAACGCTCATCAGTGATATTAATAAACTCCCCGGGCAGCTCACTTA
AGTTCATGTCGCTGTCCAGCTGCTGAGCCACAGGCTGCTGTCCAACTTGCGGTTGCTTAACGGGCGGCGAAGGAGAAGTC
CACGCCTACATGGGGGTAGAGTCATAATCGTGCATCAGGATAGGGCGGTGGTGCTGCAGCAGCGCGCGAATAAACTGCT
GCCGCCGCCGCTCCGTCCTGCAGGAATACAACATGGCAGTGGTCTCCTCAGCGATGATTCGCACCGCCCGCAGCATAAGG
CGCCTTGTCCTCCGGGCACAGCAGCGCACCCTGATCTCACTTAAATCAGCACAGTAACTGCAGCACAGCACCACAATATT
GTTCAAAATCCCACAGTGCAAGGCGCTGTATCCAAAGCTCATGGCGGGGACCACAGAACCCACGTGGCCATCATACCACA
AGCGCAGGTAGATTAAGTGGCGACCCCTCATAAACACGCTGGACATAAACATTACCTCTTTTGGCATGTTGTAATTCACCA
CCTCCCGGTACCATATAAACCTCTGATTAAACATGGCGCCATCCACCACCATCCTAAACCAGCTGGCCAAAACCTGCCCGC
CGGCTATACACTGCAGGGAACCGGGACTGGAACAATGACAGTGGAGAGCCCAGGACTCGTAACCATGGATCATCATGCT
CGTCATGATATCAATGTTGGCACAACACAGGCACACGTGCATACACTTCCTCAGGATTACAAGCTCCTCCCGCGTTAGAAC
CATATCCCAGGGAACAACCCATTCCTGAATCAGCGTAAATCCCACACTGCAGGGAAGACCTCGCACGTAACTCACGTTGT
GCATTGTCAAAGTGTTACATTCGGGCAGCAGCGGATGATCCTCCAGTATGGTAGCGCGGGTTTCTGTCTCAAAAGGAGGTA
GACGATCCCTACTGTACGGAGTGCGCCGAGACAACCGAGATCGTGTTGGTCGTAGTGTCATGCCAAATGGAACGCCGGAC
GTAGTCATATTTCCTGAAGCAAAACCAGGTGCGGGCGTGACAAACAGATCTGCGTCTCCGGTCTCGCCGCTTAGATCGCTC
TGTGTAGTAGTTGTAGTATATCCACTCTCTCAAAGCATCCAGGCGCCCCCTGGCTTCGGGTTCTATGTAAACTCCTTCATGC
GCCGCTGCCCTGATAACATCCACCACCGCAGAATAAGCCACACCCAGCCAACCTACACATTCGTTCTGCGAGTCACACAC
GGGAGGAGCGGGAAGAGCTGGAAGAACCATGTTTTTTTTTTTATTCCAAAAGATTATCCAAAACCTCAAAATGAAGATCTA
TTAAGTGAACGCGCTCCCCTCCGGTGGCGTGGTCAAACTCTACAGCCAAAGAACAGATAATGGCATTTGTAAGATGTTGCA
CAATGGCTTCCAAAAGGCAAACGGCCCTCACGTCCAAGTGGACGTAAAGGCTAAACCCTTCAGGGTGAATCTCCTCTATA
AACATTCCAGCACCTTCAACCATGCCCAAATAATTCTCATCTCGCCACCTTCTCAATATATCTCTAAGCAAATCCCGAATATT
AAGTCCGGCCATTGTAAAAATCTGCTCCAGAGCGCCCTCCACCTTCAGCCTCAAGCAGCGAATCATGATTGCAAAAATTCA
GGTTCCTCACAGACCTGTATAAGATTCAAAAGCGGAACATTAACAAAAATACCGCGATCCCGTAGGTCCCTTCGCAGGGC
CAGCTGAACATAATCGTGCAGGTCTGCACGGACCAGCGCGGCCACTTCCCCGCCAGGAACCATGACAAAAGAACCCACA
CTGATTATGACACGCATACTCGGAGCTATGCTAACCAGCGTAGCCCCGATGTAAGCTTGTTGCATGGGCGGCGATATAAAA
TGCAAGGTGCTGCTCAAAAAATCAGGCAAAGCCTCGCGCAAAAAAGAAAGCACATCGTAGTCATGCTCATGCAGATAAAG
GCAGGTAAGCTCCGGAACCACCACAGAAAAAGACACCATTTTTCTCTCAAACATGTCTGCGGGTTTCTGCATAAACACAAA

ATAAAATAACAAAAAAACATTTAAACATTAGAAGCCTGTCTTACAACAGGAAAAACAACCCTTATAAGCATAAGACGGACT
ACGGCCATGCCGGCGTGACCGTAAAAAAACTGGTCACCGTGATTAAAAAGCACCACCGACAGCTCCTCGGTCATGTCCGG
AGTCATAATGTAAGACTCGGTAAACACATCAGGTTGATTCACATCGGTCAGTGCTAAAAAGCGACCGAAATAGCCCGGGG
GAATACATACCCGCAGGCGTAGAGACAACATTACAGCCCCCATAGGAGGTATAACAAAATTAATAGGAGAGAAAAACAC
ATAAACACCTGAAAAACCCTCCTGCCTAGGCAAAATAGCACCCTCCCGCTCCAGAACAACATACAGCGCTTCCACAGCGG
CAGCCATAACAGTCAGCCTTACCAGTAAAAAAGAAAACCTATTAAAAAAACACCACTCGACACGGCACCAGCTCAATCAG
TCACAGTGTAAAAAAGGGCCAAGTGCAGAGCGAGTATATATAGGACTAAAAAATGACGTAACGGTTAAAGTCCACAAAAA
ACACCCAGAAAACCGCACGCGAACCTACGCCCAGAAACGAAAGCCAAAAAACCCACAACTTCCTCAAATCGTCACTTCC
GTTTTCCCACGTTACGTCACTTCCCATTTTAAGAAAACTACAATTCCCAACACATACAAGTTACTCCGCCCTAAAACCTACG
TCACCCGCCCCGTTCCCACGCCCCGCGCCACGTCACAAACTCCACCCCCTCATTATCATATTGGCTTCAATCCAAAATAAG
GTATATTATTGATGATGTTAATTAATTTAAATCCGCATGCGATATCGAGCTCTCCCGGGAATTCGGATCTGCGACGCGAGGC
TGGATGGCCTTCCCCATTATGATTCTTCTCGCTTCCGGCGGCATCGGGATGCCCGCGTTGCAGGCCATGCTGTCCAGGCAG
GTAGATGACGACCATCAGGGACAGCTTCAAGGCCAGCAAAAGGCCAGGAACCGTAAAAAGGCCGCGTTGCTGGCGTTTT
TCCATAGGCTCCGCCCCCCTGACGAGCATCACAAAAATCGACGCTCAAGTCAGAGGTGGCGAAACCCGACAGGACTATA
AAGATACCAGGCGTTTCCCCCTGGAAGCTCCCTCGTGCGCTCTCCTGTTCCGACCCTGCCGCTTACCGGATACCTGTCCGC
CTTTCTCCCTTCGGGAAGCGTGGCGCTTTCTCATAGCTCACGCTGTAGGTATCTCAGTTCGGTGTAGGTCGTTCGCTCCAAG
CTGGGCTGTGTGCACGAACCCCCCGTTCAGCCCGACCGCTGCGCCTTATCCGGTAACTATCGTCTTGAGTCCAACCCGGTA
AGACACGACTTATCGCCACTGGCAGCAGCCACTGGTAACAGGATTAGCAGAGCGAGGTATGTAGGCGGTGCTACAGAGT
TCTTGAAGTGGTGGCCTAACTACGGCTACACTAGAAGGACAGTATTTGGTATCTGCGCTCTGCTGAAGCCAGTTACCTTCG
GAAAAAGAGTTGGTAGCTCTTGATCCGGCAAACAAACCACCGCTGGTAGCGGTGGTTTTTTTGTTTGCAAGCAGCAGATTA
CGCGCAGAAAAAAAGGATCTCAAGAAGATCCTTTGATCTTTTCTACGGGGTCTGACGCTCAGTGGAACGAAAACTCACGTT
AAGGGATTTTGGTCATGAGATTATCAAAAAGGATCTTCACCTAGATCCTTTTAAATCAATCTAAAGTATATATGAGTAAACTT
GGTCTGACAGTTACCAATGCTTAATCAGTGAGGCACCTATCTCAGCGATCTGTCTATTTCGTTCATCCATAGTTGCCTGACT
CCCCGTCGTGTAGATAACTACGATACGGGAGGGCTTACCATCTGGCCCCAGTGCTGCAATGATACCGCGAGACCCACGCT
CACCGGCTCCAGATTTATCAGCAATAAACCAGCCAGCCGGAAGGGCCGAGCGCAGAAGTGGTCCTGCAACTTTATCCGCC
TCCATCCAGTCTATTAATTGTTGCCGGGAAGCTAGAGTAAGTAGTTCGCCAGTTAATAGTTTGCGCAACGTTGTTGCCATTG
CTACAGGCATCGTGGTGTCACGCTCGTCGTTTGGTATGGCTTCATTCAGCTCCGGTTCCCAACGATCAAGGCGAGTTACAT
GATCCCCCATGTTGTGCAAAAAAGCGGTTAGCTCCTTCGGTCCTCCGATCGTTGTCAGAAGTAAGTTGGCCGCAGTGTTAT
CACTCATGGTTATGGCAGCACTGCATAATTCTCTTACTGTCATGCCATCCGTAAGATGCTTTTCTGTGACTGGTGAGTACTC
AACCAAGTCATTCTGAGAATAGTGTATGCGGCGACCGAGTTGCTCTTGCCCGGCGTCAACACGGGATAATACCGCGCCAC
ATAGCAGAACTTTAAAAGTGCTCATCATTGGAAAACGTTCTTCGGGGCGAAAACTCTCAAGGATCTTACCGCTGTTGAGAT
CCAGTTCGATGTAACCCACTCGTGCACCCAACTGATCTTCAGCATCTTTTACTTTCACCAGCGTTTCTGGGTGAGCAAAAAC
AGGAAGGCAAAATGCCGCAAAAAAGGGAATAAGGGCGACACGGAAATGTTGAATACTCATACTCTTCCTTTTTCAATATTA
TTGAAGCATTTATCAGGGTTATTGTCTCATGAGCGGATACATATTTGAATGTATTTAGAAAAATAAACAAATAGGGGTTCCG
CGCACATTTCCCCGAAAAGTGCCACCTGACGTCTAAGAAACCATTATTATCATGACATTAACCTATAAAAATAGGCGTATC
ACGAGGCCCTTTCGTCTTCAAGAATTGGATCCGAATTCCCGGGAGAGCTCGATATCGCATGCGGATTTAAATTAATTAA

pAV-UL25-3×GS-EGFP (ADV-UL25)

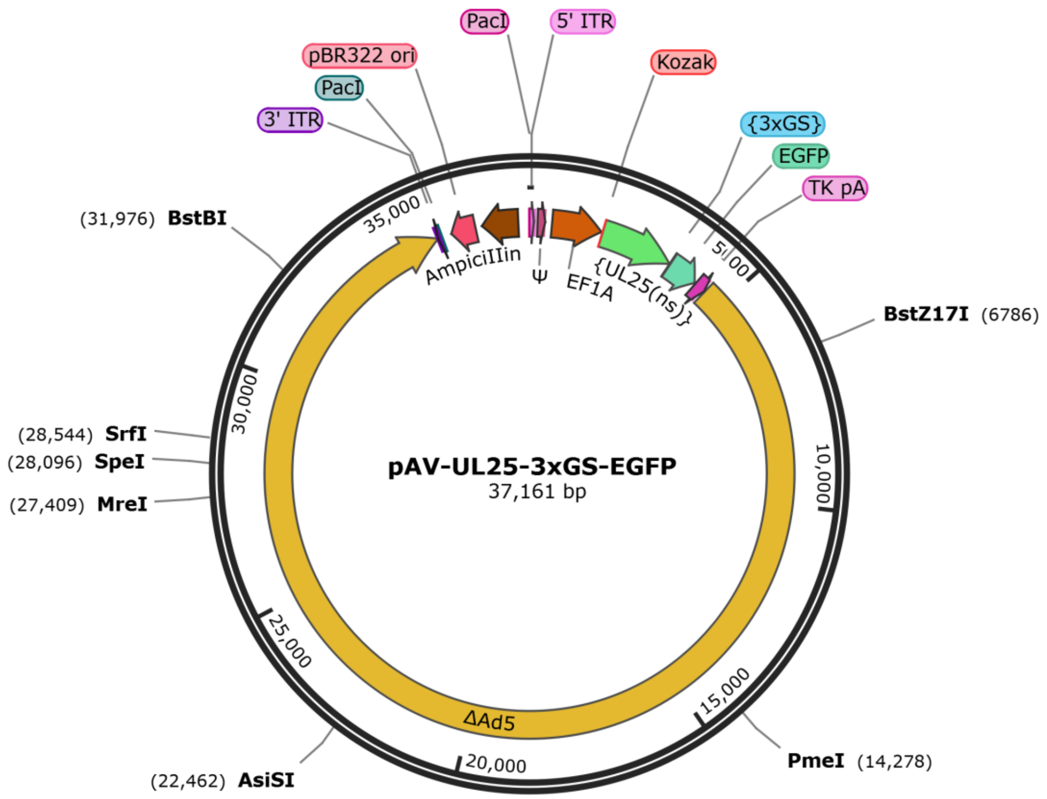

CATCATCAATAATATACCTTATTTTGGATTGAAGCCAATATGATAATGAGGGGGTGGAGTTTGTGACGTGGCGCGGGGCGT
GGGAACGGGGCGGGTGACGTAGTAGTGTGGCGGAAGTGTGATGTTGCAAGTGTGGCGGAACACATGTAAGCGACGGATG
TGGCAAAAGTGACGTTTTTGGTGTGCGCCGGTGTACACAGGAAGTGACAATTTTCGCGCGGTTTTAGGCGGATGTTGTAGT
AAATTTGGGCGTAACCGAGTAAGATTTGGCCATTTTCGCGGGAAAACTGAATAAGAGGAAGTGAAATCTGAATAATTTTGT
GTTACTCATAGCGCGTAATATTTGTCTAGGGCCGCGGGGACTTTGACCGTTTACGTGGAGACTCGCCCAGGTGTTTTTCTCA
GGTGTTTTCCGCGTTCCGGGTCAAAGTTGGCGTTTTATTATTATAGTCAGTCGAAGCTTGGATCCGGTACCTCTAGAATTCTC
GAGCGGCCGCTAGCGACATCGATCAACTTTGTATAGAAAAGTTGGGCTCCGGTGCCCGTCAGTGGGCAGAGCGCACATCG
CCCACAGTCCCCGAGAAGTTGGGGGGAGGGGTCGGCAATTGAACCGGTGCCTAGAGAAGGTGGCGCGGGGTAAACTGG
GAAAGTGATGTCGTGTACTGGCTCCGCCTTTTTCCCGAGGGTGGGGGAGAACCGTATATAAGTGCAGTAGTCGCCGTGAA
CGTTCTTTTTCGCAACGGGTTTGCCGCCAGAACACAGGTAAGTGCCGTGTGTGGTTCCCGCGGGCCTGGCCTCTTTACGGG
TTATGGCCCTTGCGTGCCTTGAATTACTTCCACCTGGCTGCAGTACGTGATTCTTGATCCCGAGCTTCGGGTTGGAAGTGGG
TGGGAGAGTTCGAGGCCTTGCGCTTAAGGAGCCCCTTCGCCTCGTGCTTGAGTTGAGGCCTGGCCTGGGCGCTGGGGCCG
CCGCGTGCGAATCTGGTGGCACCTTCGCGCCTGTCTCGCTGCTTTCGATAAGTCTCTAGCCATTTAAAATTTTTGATGACCT
GCTGCGACGCTTTTTTTCTGGCAAGATAGTCTTGTAAATGCGGGCCAAGATCTGCACACTGGTATTTCGGTTTTTGGGGCCG
CGGGCGGCGACGGGGCCCGTGCGTCCCAGCGCACATGTTCGGCGAGGCGGGGCCTGCGAGCGCGGCCACCGAGAATCG
GACGGGGGTAGTCTCAAGCTGGCCGGCCTGCTCTGGTGCCTGGTCTCGCGCCGCCGTGTATCGCCCCGCCCTGGGCGGC
AAGGCTGGCCCGGTCGGCACCAGTTGCGTGAGCGGAAAGATGGCCGCTTCCCGGCCCTGCTGCAGGGAGCTCAAAATGG
AGGACGCGGCGCTCGGGAGAGCGGGCGGGTGAGTCACCCACACAAAGGAAAAGGGCCTTTCCGTCCTCAGCCGTCGCTT
CATGTGACTCCACGGAGTACCGGGCGCCGTCCAGGCACCTCGATTAGTTCTCGAGCTTTTGGAGTACGTCGTCTTTAGGTT
GGGGGGAGGGGTTTTATGCGATGGAGTTTCCCCACACTGAGTGGGTGGAGACTGAAGTTAGGCCAGCTTGGCACTTGATG
TAATTCTCCTTGGAATTTGCCCTTTTTGAGTTTGGATCTTGGTTCATTCTCAAGCCTCAGACAGTGGTTCAAAGTTTTTTTCTT
CCATTTCAGGTGTCGTGACAAGTTTGTACAAAAAAGCAGGCTGCCACCATGGACCCGTACTGCCCATTTGACGCTCTGGAC
GTCTGGGAACACAGGCGCTTCATAGTCGCCGATTCCCGAAACTTCATCACCCCCGAGTTCCCCCGGGACTTTTGGATGTCG
CCCGTCTTTAACCTCCCCCGGGAGACGGCGGCGGAGCAGGTGGTCGTCCTACAGGCCCAGCGCACAGCGGCTGCCGCTG
CCCTGGAGAACGCCGCCATGCAGGCGGCCGAGCTCCCCGTCGATATCGAGCGCCGGTTACGCCCGATCGAACGGAACGT

GCACGAGATCGCAGGCGCCCTGGAGGCGCTGGAGACGGCGGCGGCCGCCGCCGAAGAGGCGGATGCCGCGCGCGGGG
ATGAGCCGGCGGGTGGGGGCGACGGGGGGGCGCCCCCGGGTCTGGCCGTCGCGGAGATGGAGGTCCAGATCGTGCGCA
ACGACCCGCCGCTACGATACGACACCAACCTCCCCGTGGATCTGCTACACATGGTGTACGCGGGCCGCGGGGCGACCGG
CTCGTCGGGGGTGGTGTTCGGGACCTGGTACCGCACTATCCAGGACCGCACCATCACGGACTTTCCCCTGACCACCCGCA
GTGCCGACTTTCGGGACGGCCGTATGTCCAAGACCTTCATGACGGCGCTGGTACTGTCCCTGCAGGCGTGCGGCCGGCTG
TATGTGGGCCAGCGCCACTATTCCGCCTTCGAGTGCGCCGTGTTGTGTCTCTACCTGCTGTACCGAAACACGCACGGGGCC
GCCGACGATAGCGACCGCGCTCCGGTCACGTTCGGGGATCTGCTGGGCCGGCTGCCCCGCTACCTGGCGTGCCTGGCCG
CGGTGATCGGGACCGAGGGCGGCCGGCCACAGTACCGCTACCGCGACGACAAGCTCCCCAAGACGCAGTTCGCGGCCG
GCGGGGGCCGCTACGAACACGGAGCGCTGGCGTCGCACATCGTGATCGCCACGCTGATGCACCACGGGGTGCTCCCGGC
GGCCCCGGGGGACGTCCCCCGGGACGCGAGTACCCACGTTAACCCCGACGGCGTGGCGCACCACGACGACATAAACCG
CGCCGCCGCCGCGTTCCTCAGCCGGGGCCACAACCTATTCCTGTGGGAGGACCAGACTCTGCTGCGGGCAACCGCGAAC
ACCATAACGGCCCTGGGCGTTATCCAGCGGCTCCTCGCGAACGGCAACGTGTACGCGGACCGCCTCAACAACCGCCTGC
AGCTGGGCATGCTGATCCCCGGAGCCGTCCCTTCGGAGGCCATCGCCCGTGGGGCCTCCGGGTCCGACTCGGGGGCCAT
CAAGAGCGGAGACAACAATCTGGAGGCGCTATGTGCCAATTACGTGCTTCCGCTGTACCGGGCCGACCCGGCGGTCGAG
CTGACCCAGCTGTTTCCCGGCCTGGCCGCCCTGTGTCTTGACGCCCAGGCGGGGCGGCCGGTCGGGTCGACGCGGCGGG
TGGTGGATATGTCATCGGGGGCCCGCCAGGCGGCGCTGGTGCGCCTCACCGCCCTGGAACTCATCAACCGCACCCGCAC
AAACCCCACCCCTGTGGGGGAGGTTATCCACGCCCACGACGCCCTGGCGATCCAATACGAACAGGGGCTTGGCCTGCTG
GCGCAGCAGGCACGCATTGGCTTGGGCTCCAACACCAAGCGTTTCTCCGCGTTCAACGTTAGCAGCGACTACGACATGTT
GTACTTTTTATGTCTGGGGTTCATTCCACAGTACCTGTCGGCGGTTGGCAGCGGCAGCGGCAGCATGGTGAGCAAGGGCG
AGGAGCTGTTCACCGGGGTGGTGCCCATCCTGGTCGAGCTGGACGGCGACGTAAACGGCCACAAGTTCAGCGTGTCCGG
CGAGGGCGAGGGCGATGCCACCTACGGCAAGCTGACCCTGAAGTTCATCTGCACCACCGGCAAGCTGCCCGTGCCCTGG
CCCACCCTCGTGACCACCCTGACCTACGGCGTGCAGTGCTTCAGCCGCTACCCCGACCACATGAAGCAGCACGACTTCTT
CAAGTCCGCCATGCCCGAAGGCTACGTCCAGGAGCGCACCATCTTCTTCAAGGACGACGGCAACTACAAGACCCGCGCC
GAGGTGAAGTTCGAGGGCGACACCCTGGTGAACCGCATCGAGCTGAAGGGCATCGACTTCAAGGAGGACGGCAACATCC
TGGGGCACAAGCTGGAGTACAACTACAACAGCCACAACGTCTATATCATGGCCGACAAGCAGAAGAACGGCATCAAGGT
GAACTTCAAGATCCGCCACAACATCGAGGACGGCAGCGTGCAGCTCGCCGACCACTACCAGCAGAACACCCCCATCGGC
GACGGCCCCGTGCTGCTGCCCGACAACCACTACCTGAGCACCCAGTCCGCCCTGAGCAAAGACCCCAACGAGAAGCGCG
ATCACATGGTCCTGCTGGAGTTCGTGACCGCCGCCGGGATCACTCTCGGCATGGACGAGCTGTACAAGTAAACCCAGCTT
TCTTGTACAAAGTGGTGGGGGAGGCTAACTGAAACACGGAAGGAGACAATACCGGAAGGAACCCGCGCTATGACGGCAA
TAAAAAGACAGAATAAAACGCACGGGTGTTGGGTCGTTTGTTCATAAACGCGGGGTTCGGTCCCAGGGCTGGCACTCTGT
CGATACCCCACCGAGACCCCATTGGGGCCAATACGCCCGCGTTTCTTCCTTTTCCCCACCCCACCCCCCAAGTTCGGGTGA
AGGCCCAGGGCTCGCAGCCAACGTCGGGGCGGCAGGCCCTGCCATAGCGATCGATTCGACAGATCACTGAAATGTGTGG
GCGTGGCTTAAGGGTGGGAAAGAATATATAAGGTGGGGGTCTTATGTAGTTTTGTATCTGTTTTGCAGCAGCCGCCGCCGC
CATGAGCACCAACTCGTTTGATGGAAGCATTGTGAGCTCATATTTGACAACGCGCATGCCCCCATGGGCCGGGGTGCGTC
AGAATGTGATGGGCTCCAGCATTGATGGTCGCCCCGTCCTGCCCGCAAACTCTACTACCTTGACCTACGAGACCGTGTCTG
GAACGCCGTTGGAGACTGCAGCCTCCGCCGCCGCTTCAGCCGCTGCAGCCACCGCCCGCGGGATTGTGACTGACTTTGCT
TTCCTGAGCCCGCTTGCAAGCAGTGCAGCTTCCCGTTCATCCGCCCGCGATGACAAGTTGACGGCTCTTTTGGCACAATTG
GATTCTTTGACCCGGGAACTTAATGTCGTTTCTCAGCAGCTGTTGGATCTGCGCCAGCAGGTTTCTGCCCTGAAGGCTTCCT
CCCCTCCCAATGCGGTTTAAAACATAAATAAAAAACCAGACTCTGTTTGGATTTGGATCAAGCAAGTGTCTTGCTGTCTTTA
TTTAGGGGTTTTGCGCGCGCGGTAGGCCCGGGACCAGCGGTCTCGGTCGTTGAGGGTCCTGTGTATTTTTTCCAGGACGTG
GTAAAGGTGACTCTGGATGTTCAGATACATGGGCATAAGCCCGTCTCTGGGGTGGAGGTAGCACCACTGCAGAGCTTCAT
GCTGCGGGGTGGTGTTGTAGATGATCCAGTCGTAGCAGGAGCGCTGGGCGTGGTGCCTAAAAATGTCTTTCAGTAGCAAG
CTGATTGCCAGGGGCAGGCCCTTGGTGTAAGTGTTTACAAAGCGGTTAAGCTGGGATGGGTGCATACGTGGGGATATGAG
ATGCATCTTGGACTGTATTTTTAGGTTGGCTATGTTCCCAGCCATATCCCTCCGGGGATTCATGTTGTGCAGAACCACCAGC
ACAGTGTATCCGGTGCACTTGGGAAATTTGTCATGTAGCTTAGAAGGAAATGCGTGGAAGAACTTGGAGACGCCCTTGTG
ACCTCCAAGATTTTCCATGCATTCGTCCATAATGATGGCAATGGGCCCACGGGCGGCGGCCTGGGCGAAGATATTTCTGG
GATCACTAACGTCATAGTTGTGTTCCAGGATGAGATCGTCATAGGCCATTTTTACAAAGCGCGGGCGGAGGGTGCCAGAC
TGCGGTATAATGGTTCCATCCGGCCCAGGGGCGTAGTTACCCTCACAGATTTGCATTTCCCACGCTTTGAGTTCAGATGGG
GGGATCATGTCTACCTGCGGGGCGATGAAGAAAACGGTTTCCGGGGTAGGGGAGATCAGCTGGGAAGAAAGCAGGTTCC

TGAGCAGCTGCGACTTACCGCAGCCGGTGGGCCCGTAAATCACACCTATTACCGGCTGCAACTGGTAGTTAAGAGAGCTG
CAGCTGCCGTCATCCCTGAGCAGGGGGGCCACTTCGTTAAGCATGTCCCTGACTCGCATGTTTTCCCTGACCAAATCCGCC
AGAAGGCGCTCGCCGCCCAGCGATAGCAGTTCTTGCAAGGAAGCAAAGTTTTTCAACGGTTTGAGACCGTCCGCCGTAGG
CATGCTTTTGAGCGTTTGACCAAGCAGTTCCAGGCGGTCCCACAGCTCGGTCACCTGCTCTACGGCATCTCGATCCAGCAT
ATCTCCTCGTTTCGCGGGTTGGGGCGGCTTTCGCTGTACGGCAGTAGTCGGTGCTCGTCCAGACGGGCCAGGGTCATGTCT
TTCCACGGGCGCAGGGTCCTCGTCAGCGTAGTCTGGGTCACGGTGAAGGGGTGCGCTCCGGGCTGCGCGCTGGCCAGGG
TGCGCTTGAGGCTGGTCCTGCTGGTGCTGAAGCGCTGCCGGTCTTCGCCCTGCGCGTCGGCCAGGTAGCATTTGACCATG
GTGTCATAGTCCAGCCCCTCCGCGGCGTGGCCCTTGGCGCGCAGCTTGCCCTTGGAGGAGGCGCCGCACGAGGGGCAGT
GCAGACTTTTGAGGGCGTAGAGCTTGGGCGCGAGAAATACCGATTCCGGGGAGTAGGCATCCGCGCCGCAGGCCCCGCA
GACGGTCTCGCATTCCACGAGCCAGGTGAGCTCTGGCCGTTCGGGGTCAAAAACCAGGTTTCCCCCATGCTTTTTGATGCG
TTTCTTACCTCTGGTTTCCATGAGCCGGTGTCCACGCTCGGTGACGAAAAGGCTGTCCGTGTCCCCGTATACAGACTTGAG
AGGCCTGTCCTCGAGCGGTGTTCCGCGGTCCTCCTCGTATAGAAACTCGGACCACTCTGAGACAAAGGCTCGCGTCCAGG
CCAGCACGAAGGAGGCTAAGTGGGAGGGGTAGCGGTCGTTGTCCACTAGGGGGTCCACTCGCTCCAGGGTGTGAAGACA
CATGTCGCCCTCTTCGGCATCAAGGAAGGTGATTGGTTTGTAGGTGTAGGCCACGTGACCGGGTGTTCCTGAAGGGGGGC
TATAAAAGGGGGTGGGGGCGCGTTCGTCCTCACTCTCTTCCGCATCGCTGTCTGCGAGGGCCAGCTGTTGGGGTGAGTAC
TCCCTCTGAAAAGCGGGCATGACTTCTGCGCTAAGATTGTCAGTTTCCAAAAACGAGGAGGATTTGATATTCACCTGGCCC
GCGGTGATGCCTTTGAGGGTGGCCGCATCCATCTGGTCAGAAAAGACAATCTTTTTGTTGTCAAGCTTGGTGGCAAACGAC
CCGTAGAGGGCGTTGGACAGCAACTTGGCGATGGAGCGCAGGGTTTGGTTTTTGTCGCGATCGGCGCGCTCCTTGGCCGC
GATGTTTAGCTGCACGTATTCGCGCGCAACGCACCGCCATTCGGGAAAGACGGTGGTGCGCTCGTCGGGCACCAGGTGC
ACGCGCCAACCGCGGTTGTGCAGGGTGACAAGGTCAACGCTGGTGGCTACCTCTCCGCGTAGGCGCTCGTTGGTCCAGCA
GAGGCGGCCGCCCTTGCGCGAGCAGAATGGCGGTAGGGGGTCTAGCTGCGTCTCGTCCGGGGGGTCTGCGTCCACGGTA
AAGACCCCGGGCAGCAGGCGCGCGTCGAAGTAGTCTATCTTGCATCCTTGCAAGTCTAGCGCCTGCTGCCATGCGCGGGC
GGCAAGCGCGCGCTCGTATGGGTTGAGTGGGGGACCCCATGGCATGGGGTGGGTGAGCGCGGAGGCGTACATGCCGCA
AATGTCGTAAACGTAGAGGGGCTCTCTGAGTATTCCAAGATATGTAGGGTAGCATCTTCCACCGCGGATGCTGGCGCGCA
CGTAATCGTATAGTTCGTGCGAGGGAGCGAGGAGGTCGGGACCGAGGTTGCTACGGGCGGGCTGCTCTGCTCGGAAGAC
TATCTGCCTGAAGATGGCATGTGAGTTGGATGATATGGTTGGACGCTGGAAGACGTTGAAGCTGGCGTCTGTGAGACCTA
CCGCGTCACGCACGAAGGAGGCGTAGGAGTCGCGCAGCTTGTTGACCAGCTCGGCGGTGACCTGCACGTCTAGGGCGCA
GTAGTCCAGGGTTTCCTTGATGATGTCATACTTATCCTGTCCCTTTTTTTTCCACAGCTCGCGGTTGAGGACAAACTCTTCGC
GGTCTTTCCAGTACTCTTGGATCGGAAACCCGTCGGCCTCCGAACGGTAAGAGCCTAGCATGTAGAACTGGTTGACGGCC
TGGTAGGCGCAGCATCCCTTTTCTACGGGTAGCGCGTATGCCTGCGCGGCCTTCCGGAGCGAGGTGTGGGTGAGCGCAAA
GGTGTCCCTGACCATGACTTTGAGGTACTGGTATTTGAAGTCAGTGTCGTCGCATCCGCCCTGCTCCCAGAGCAAAAAGTC
CGTGCGCTTTTTGGAACGCGGATTTGGCAGGGCGAAGGTGACATCGTTGAAGAGTATCTTTCCCGCGCGAGGCATAAAGT
TGCGTGTGATGCGGAAGGGTCCCGGCACCTCGGAACGGTTGTTAATTACCTGGGCGGCGAGCACGATCTCGTCAAAGCCG
TTGATGTTGTGGCCCACAATGTAAAGTTCCAAGAAGCGCGGGATGCCCTTGATGGAAGGCAATTTTTTAAGTTCCTCGTAG
GTGAGCTCTTCAGGGGAGCTGAGCCCGTGCTCTGAAAGGGCCCAGTCTGCAAGATGAGGGTTGGAAGCGACGAATGAGC
TCCACAGGTCACGGGCCATTAGCATTTGCAGGTGGTCGCGAAAGGTCCTAAACTGGCGACCTATGGCCATTTTTTCTGGGG
TGATGCAGTAGAAGGTAAGCGGGTCTTGTTCCCAGCGGTCCCATCCAAGGTTCGCGGCTAGGTCTCGCGCGGCAGTCACT
AGAGGCTCATCTCCGCCGAACTTCATGACCAGCATGAAGGGCACGAGCTGCTTCCCAAAGGCCCCCATCCAAGTATAGGT
CTCTACATCGTAGGTGACAAAGAGACGCTCGGTGCGAGGATGCGAGCCGATCGGGAAGAACTGGATCTCCCGCCACCAA
TTGGAGGAGTGGCTATTGATGTGGTGAAAGTAGAAGTCCCTGCGACGGGCCGAACACTCGTGCTGGCTTTTGTAAAAACG
TGCGCAGTACTGGCAGCGGTGCACGGGCTGTACATCCTGCACGAGGTTGACCTGACGACCGCGCACAAGGAAGCAGAGT
GGGAATTTGAGCCCCTCGCCTGGCGGGTTTGGCTGGTGGTCTTCTACTTCGGCTGCTTGTCCTTGACCGTCTGGCTGCTCGA
GGGGAGTTACGGTGGATCGGACCACCACGCCGCGCGAGCCCAAAGTCCAGATGTCCGCGCGCGGCGGTCGGAGCTTGAT
GACAACATCGCGCAGATGGGAGCTGTCCATGGTCTGGAGCTCCCGCGGCGTCAGGTCAGGCGGGAGCTCCTGCAGGTTT
ACCTCGCATAGACGGGTCAGGGCGCGGGCTAGATCCAGGTGATACCTAATTTCCAGGGGCTGGTTGGTGGCGGCGTCGAT
GGCTTGCAAGAGGCCGCATCCCCGCGGCGCGACTACGGTACCGCGCGGCGGGCGGTGGGCCGCGGGGGTGTCCTTGGA
TGATGCATCTAAAAGCGGTGACGCGGGCGAGCCCCCGGAGGTAGGGGGGGCTCCGGACCCGCCGGGAGAGGGGGCAG
GGGCACGTCGGCGCCGCGCGCGGGCAGGAGCTGGTGCTGCGCGCGTAGGTTGCTGGCGAACGCGACGACGCGGCGGTT
GATCTCCTGAATCTGGCGCCTCTGCGTGAAGACGACGGGCCCGGTGAGCTTGAACCTGAAAGAGAGTTCGACAGAATCAA

TTTCGGTGTCGTTGACGGCGGCCTGGCGCAAAATCTCCTGCACGTCTCCTGAGTTGTCTTGATAGGCGATCTCGGCCATGA
ACTGCTCGATCTCTTCCTCCTGGAGATCTCCGCGTCCGGCTCGCTCCACGGTGGCGGCGAGGTCGTTGGAAATGCGGGCC
ATGAGCTGCGAGAAGGCGTTGAGGCCTCCCTCGTTCCAGACGCGGCTGTAGACCACGCCCCCTTCGGCATCGCGGGCGC
GCATGACCACCTGCGCGAGATTGAGCTCCACGTGCCGGGCGAAGACGGCGTAGTTTCGCAGGCGCTGAAAGAGGTAGTT
GAGGGTGGTGGCGGTGTGTTCTGCCACGAAGAAGTACATAACCCAGCGTCGCAACGTGGATTCGTTGATATCCCCCAAGG
CCTCAAGGCGCTCCATGGCCTCGTAGAAGTCCACGGCGAAGTTGAAAAACTGGGAGTTGCGCGCCGACACGGTTAACTCC
TCCTCCAGAAGACGGATGAGCTCGGCGACAGTGTCGCGCACCTCGCGCTCAAAGGCTACAGGGGCCTCTTCTTCTTCTTCA
ATCTCCTCTTCCATAAGGGCCTCCCCTTCTTCTTCTTCTGGCGGCGGTGGGGGAGGGGGGACACGGCGGCGACGACGGCG
CACCGGGAGGCGGTCGACAAAGCGCTCGATCATCTCCCCGCGGCGACGGCGCATGGTCTCGGTGACGGCGCGGCCGTTC
TCGCGGGGGCGCAGTTGGAAGACGCCGCCCGTCATGTCCCGGTTATGGGTTGGCGGGGGGCTGCCATGCGGCAGGGATA
CGGCGCTAACGATGCATCTCAACAATTGTTGTGTAGGTACTCCGCCGCCGAGGGACCTGAGCGAGTCCGCATCGACCGGA
TCGGAAAACCTCTCGAGAAAGGCGTCTAACCAGTCACAGTCGCAAGGTAGGCTGAGCACCGTGGCGGGCGGCAGCGGG
CGGCGGTCGGGGTTGTTTCTGGCGGAGGTGCTGCTGATGATGTAATTAAAGTAGGCGGTCTTGAGACGGCGGATGGTCGA
CAGAAGCACCATGTCCTTGGGTCCGGCCTGCTGAATGCGCAGGCGGTCGGCCATGCCCCAGGCTTCGTTTTGACATCGGC
GCAGGTCTTTGTAGTAGTCTTGCATGAGCCTTTCTACCGGCACTTCTTCTTCTCCTTCCTCTTGTCCTGCATCTCTTGCATCTA
TCGCTGCGGCGGCGGCGGAGTTTGGCCGTAGGTGGCGCCCTCTTCCTCCCATGCGTGTGACCCCGAAGCCCCTCATCGGC
TGAAGCAGGGCTAGGTCGGCGACAACGCGCTCGGCTAATATGGCCTGCTGCACCTGCGTGAGGGTAGACTGGAAGTCAT
CCATGTCCACAAAGCGGTGGTATGCGCCCGTGTTGATGGTGTAAGTGCAGTTGGCCATAACGGACCAGTTAACGGTCTGG
TGACCCGGCTGCGAGAGCTCGGTGTACCTGAGACGCGAGTAAGCCCTCGAGTCAAATACGTAGTCGTTGCAAGTCCGCAC
CAGGTACTGGTATCCCACCAAAAAGTGCGGCGGCGGCTGGCGGTAGAGGGGCCAGCGTAGGGTGGCCGGGGCTCCGGG
GGCGAGATCTTCCAACATAAGGCGATGATATCCGTAGATGTACCTGGACATCCAGGTGATGCCGGCGGCGGTGGTGGAG
GCGCGCGGAAAGTCGCGGACGCGGTTCCAGATGTTGCGCAGCGGCAAAAAGTGCTCCATGGTCGGGACGCTCTGGCCGG
TCAGGCGCGCGCAATCGTTGACGCTCTAGACCGTGCAAAAGGAGAGCCTGTAAGCGGGCACTCTTCCGTGGTCTGGTGGA
TAAATTCGCAAGGGTATCATGGCGGACGACCGGGGTTCGAGCCCCGTATCCGGCCGTCCGCCGTGATCCATGCGGTTACC
GCCCGCGTGTCGAACCCAGGTGTGCGACGTCAGACAACGGGGGAGTGCTCCTTTTGGCTTCCTTCCAGGCGCGGCGGCTG
CTGCGCTAGCTTTTTTGGCCACTGGCCGCGCGCAGCGTAAGCGGTTAGGCTGGAAAGCGAAAGCATTAAGTGGCTCGCTC
CCTGTAGCCGGAGGGTTATTTTCCAAGGGTTGAGTCGCGGGACCCCCGGTTCGAGTCTCGGACCGGCCGGACTGCGGCG
AACGGGGGTTTGCCTCCCCGTCATGCAAGACCCCGCTTGCAAATTCCTCCGGAAACAGGGACGAGCCCCTTTTTTGCTTTT
CCCAGATGCATCCGGTGCTGCGGCAGATGCGCCCCCCTCCTCAGCAGCGGCAAGAGCAAGAGCAGCGGCAGACATGCA
GGGCACCCTCCCCTCCTCCTACCGCGTCAGGAGGGGCGACATCCGCGGTTGACGCGGCAGCAGATGGTGATTACGAACC
CCCGCGGCGCCGGGCCCGGCACTACCTGGACTTGGAGGAGGGCGAGGGCCTGGCGCGGCTAGGAGCGCCCTCTCCTGA
GCGGCACCCAAGGGTGCAGCTGAAGCGTGATACGCGTGAGGCGTACGTGCCGCGGCAGAACCTGTTTCGCGACCGCGAG
GGAGAGGAGCCCGAGGAGATGCGGGATCGAAAGTTCCACGCAGGGCGCGAGCTGCGGCATGGCCTGAATCGCGAGCGG
TTGCTGCGCGAGGAGGACTTTGAGCCCGACGCGCGAACCGGGATTAGTCCCGCGCGCGCACACGTGGCGGCCGCCGACC
TGGTAACCGCATACGAGCAGACGGTGAACCAGGAGATTAACTTTCAAAAAAGCTTTAACAACCACGTGCGTACGCTTGTG
GCGCGCGAGGAGGTGGCTATAGGACTGATGCATCTGTGGGACTTTGTAAGCGCGCTGGAGCAAAACCCAAATAGCAAGC
CGCTCATGGCGCAGCTGTTCCTTATAGTGCAGCACAGCAGGGACAACGAGGCATTCAGGGATGCGCTGCTAAACATAGTA
GAGCCCGAGGGCCGCTGGCTGCTCGATTTGATAAACATCCTGCAGAGCATAGTGGTGCAGGAGCGCAGCTTGAGCCTGG
CTGACAAGGTGGCCGCCATCAACTATTCCATGCTTAGCCTGGGCAAGTTTTACGCCCGCAAGATATACCATACCCCTTACG
TTCCCATAGACAAGGAGGTAAAGATCGAGGGGTTCTACATGCGCATGGCGCTGAAGGTGCTTACCTTGAGCGACGACCTG
GGCGTTTATCGCAACGAGCGCATCCACAAGGCCGTGAGCGTGAGCCGGCGGCGCGAGCTCAGCGACCGCGAGCTGATG
CACAGCCTGCAAAGGGCCCTGGCTGGCACGGGCAGCGGCGATAGAGAGGCCGAGTCCTACTTTGACGCGGGCGCTGAC
CTGCGCTGGGCCCCAAGCCGACGCGCCCTGGAGGCAGCTGGGGCCGGACCTGGGCTGGCGGTGGCACCCGCGCGCGCT
GGCAACGTCGGCGGCGTGGAGGAATATGACGAGGACGATGAGTACGAGCCAGAGGACGGCGAGTACTAAGCGGTGATG
TTTCTGATCAGATGATGCAAGACGCAACGGACCCGGCGGTGCGGGCGGCGCTGCAGAGCCAGCCGTCCGGCCTTAACTC
CACGGACGACTGGCGCCAGGTCATGGACCGCATCATGTCGCTGACTGCGCGCAATCCTGACGCGTTCCGGCAGCAGCCG
CAGGCCAACCGGCTCTCCGCAATTCTGGAAGCGGTGGTCCCGGCGCGCGCAAACCCCACGCACGAGAAGGTGCTGGCGA
TCGTAAACGCGCTGGCCGAAAACAGGGCCATCCGGCCCGACGAGGCCGGCCTGGTCTACGACGCGCTGCTTCAGCGCGT
GGCTCGTTACAACAGCGGCAACGTGCAGACCAACCTGGACCGGCTGGTGGGGGATGTGCGCGAGGCCGTGGCGCAGCG

TGAGCGCGCGCAGCAGCAGGGCAACCTGGGCTCCATGGTTGCACTAAACGCCTTCCTGAGTACACAGCCCGCCAACGTG
CCGCGGGGACAGGAGGACTACACCAACTTTGTGAGCGCACTGCGGCTAATGGTGACTGAGACACCGCAAAGTGAGGTGT
ACCAGTCTGGGCCAGACTATTTTTTCCAGACCAGTAGACAAGGCCTGCAGACCGTAAACCTGAGCCAGGCTTTCAAAAACT
TGCAGGGGCTGTGGGGGGTGCGGGCTCCCACAGGCGACCGCGCGACCGTGTCTAGCTTGCTGACGCCCAACTCGCGCCT
GTTGCTGCTGCTAATAGCGCCCTTCACGGACAGTGGCAGCGTGTCCCGGGACACATACCTAGGTCACTTGCTGACACTGTA
CCGCGAGGCCATAGGTCAGGCGCATGTGGACGAGCATACTTTCCAGGAGATTACAAGTGTCAGCCGCGCGCTGGGGCAG
GAGGACACGGGCAGCCTGGAGGCAACCCTAAACTACCTGCTGACCAACCGGCGGCAGAAGATCCCCTCGTTGCACAGTT
TAAACAGCGAGGAGGAGCGCATTTTGCGCTACGTGCAGCAGAGCGTGAGCCTTAACCTGATGCGCGACGGGGTAACGCC
CAGCGTGGCGCTGGACATGACCGCGCGCAACATGGAACCGGGCATGTATGCCTCAAACCGGCCGTTTATCAACCGCCTAA
TGGACTACTTGCATCGCGCGGCCGCCGTGAACCCCGAGTATTTCACCAATGCCATCTTGAACCCGCACTGGCTACCGCCC
CCTGGTTTCTACACCGGGGGATTCGAGGTGCCCGAGGGTAACGATGGATTCCTCTGGGACGACATAGACGACAGCGTGTT
TTCCCCGCAACCGCAGACCCTGCTAGAGTTGCAACAGCGCGAGCAGGCAGAGGCGGCGCTGCGAAAGGAAAGCTTCCGC
AGGCCAAGCAGCTTGTCCGATCTAGGCGCTGCGGCCCCGCGGTCAGATGCTAGTAGCCCATTTCCAAGCTTGATAGGGTC
TCTTACCAGCACTCGCACCACCCGCCCGCGCCTGCTGGGCGAGGAGGAGTACCTAAACAACTCGCTGCTGCAGCCGCAG
CGCGAAAAAAACCTGCCTCCGGCATTTCCCAACAACGGGATAGAGAGCCTAGTGGACAAGATGAGTAGATGGAAGACGT
ACGCGCAGGAGCACAGGGACGTGCCAGGCCCGCGCCCGCCCACCCGTCGTCAAAGGCACGACCGTCAGCGGGGTCTGG
TGTGGGAGGACGATGACTCGGCAGACGACAGCAGCGTCCTGGATTTGGGAGGGAGTGGCAACCCGTTTGCGCACCTTCG
CCCCAGGCTGGGGAGAATGTTTTAAAAAAAAAAAAAAGCATGATGCAAAATAAAAAACTCACCAAGGCCATGGCACCGAG
CGTTGGTTTTCTTGTATTCCCCTTAGTATGCGGCGCGCGGCGATGTATGAGGAAGGTCCTCCTCCCTCCTACGAGAGTGTG
GTGAGCGCGGCGCCAGTGGCGGCGGCGCTGGGTTCTCCCTTCGATGCTCCCCTGGACCCGCCGTTTGTGCCTCCGCGGTA
CCTGCGGCCTACCGGGGGGAGAAACAGCATCCGTTACTCTGAGTTGGCACCCCTATTCGACACCACCCGTGTGTACCTGG
TGGACAACAAGTCAACGGATGTGGCATCCCTGAACTACCAGAACGACCACAGCAACTTTCTGACCACGGTCATTCAAAAC
AATGACTACAGCCCGGGGGAGGCAAGCACACAGACCATCAATCTTGACGACCGGTCGCACTGGGGCGGCGACCTGAAAA
CCATCCTGCATACCAACATGCCAAATGTGAACGAGTTCATGTTTACCAATAAGTTTAAGGCGCGGGTGATGGTGTCGCGCT
TGCCTACTAAGGACAATCAGGTGGAGCTGAAATACGAGTGGGTGGAGTTCACGCTGCCCGAGGGCAACTACTCCGAGAC
CATGACCATAGACCTTATGAACAACGCGATCGTGGAGCACTACTTGAAAGTGGGCAGACAGAACGGGGTTCTGGAAAGC
GACATCGGGGTAAAGTTTGACACCCGCAACTTCAGACTGGGGTTTGACCCCGTCACTGGTCTTGTCATGCCTGGGGTATAT
ACAAACGAAGCCTTCCATCCAGACATCATTTTGCTGCCAGGATGCGGGGTGGACTTCACCCACAGCCGCCTGAGCAACTT
GTTGGGCATCCGCAAGCGGCAACCCTTCCAGGAGGGCTTTAGGATCACCTACGATGATCTGGAGGGTGGTAACATTCCCG
CACTGTTGGATGTGGACGCCTACCAGGCGAGCTTGAAAGATGACACCGAACAGGGCGGGGGTGGCGCAGGCGGCAGCA
ACAGCAGTGGCAGCGGCGCGGAAGAGAACTCCAACGCGGCAGCCGCGGCAATGCAGCCGGTGGAGGACATGAACGATC
ATGCCATTCGCGGCGACACCTTTGCCACACGGGCTGAGGAGAAGCGCGCTGAGGCCGAAGCAGCGGCCGAAGCTGCCG
CCCCCGCTGCGCAACCCGAGGTCGAGAAGCCTCAGAAGAAACCGGTGATCAAACCCCTGACAGAGGACAGCAAGAAAC
GCAGTTACAACCTAATAAGCAATGACAGCACCTTCACCCAGTACCGCAGCTGGTACCTTGCATACAACTACGGCGACCCT
CAGACCGGAATCCGCTCATGGACCCTGCTTTGCACTCCTGACGTAACCTGCGGCTCGGAGCAGGTCTACTGGTCGTTGCC
AGACATGATGCAAGACCCCGTGACCTTCCGCTCCACGCGCCAGATCAGCAACTTTCCGGTGGTGGGCGCCGAGCTGTTGC
CCGTGCACTCCAAGAGCTTCTACAACGACCAGGCCGTCTACTCCCAACTCATCCGCCAGTTTACCTCTCTGACCCACGTGT
TCAATCGCTTTCCCGAGAACCAGATTTTGGCGCGCCCGCCAGCCCCCACCATCACCACCGTCAGTGAAAACGTTCCTGCTC
TCACAGATCACGGGACGCTACCGCTGCGCAACAGCATCGGAGGAGTCCAGCGAGTGACCATTACTGACGCCAGACGCCG
CACCTGCCCCTACGTTTACAAGGCCCTGGGCATAGTCTCGCCGCGCGTCCTATCGAGCCGCACTTTTTGAGCAAGCATGTC
CATCCTTATATCGCCCAGCAATAACACAGGCTGGGGCCTGCGCTTCCCAAGCAAGATGTTTGGCGGGGCCAAGAAGCGCT
CCGACCAACACCCAGTGCGCGTGCGCGGGCACTACCGCGCGCCCTGGGGCGCGCACAAACGCGGCCGCACTGGGCGCA
CCACCGTCGATGACGCCATCGACGCGGTGGTGGAGGAGGCGCGCAACTACACGCCCACGCCGCCACCAGTGTCCACAGT
GGACGCGGCCATTCAGACCGTGGTGCGCGGAGCCCGGCGCTATGCTAAAATGAAGAGACGGCGGAGGCGCGTAGCACG
TCGCCACCGCCGCCGACCCGGCACTGCCGCCCAACGCGCGGCGGCGGCCCTGCTTAACCGCGCACGTCGCACCGGCCG
ACGGGCGGCCATGCGGGCCGCTCGAAGGCTGGCCGCGGGTATTGTCACTGTGCCCCCCAGGTCCAGGCGACGAGCGGC
CGCCGCAGCAGCCGCGGCCATTAGTGCTATGACTCAGGGTCGCAGGGGCAACGTGTATTGGGTGCGCGACTCGGTTAGC
GGCCTGCGCGTGCCCGTGCGCACCCGCCCCCCGCGCAACTAGATTGCAAGAAAAAACTACTTAGACTCGTACTGTTGTAT
GTATCCAGCGGCGGCGGCGCGCAACGAAGCTATGTCCAAGCGCAAAATCAAAGAAGAGATGCTCCAGGTCATCGCGCCG

GAGATCTATGGCCCCCGAAGAAGGAAGAGCAGGATTACAAGCCCCGAAAGCTAAAGCGGGTCAAAAAGAAAAAGAAA
GATGATGATGATGAACTTGACGACGAGGTGGAACTGCTGCACGCTACCGCGCCCAGGCGACGGGTACAGTGGAAAGGTC
GACGCGTAAAACGTGTTTTGCGACCCGGCACCACCGTAGTCTTTACGCCCGGTGAGCGCTCCACCCGCACCTACAAGCGC
GTGTATGATGAGGTGTACGGCGACGAGGACCTGCTTGAGCAGGCCAACGAGCGCCTCGGGGAGTTTGCCTACGGAAAGC
GGCATAAGGACATGCTGGCGTTGCCGCTGGACGAGGGCAACCCAACACCTAGCCTAAAGCCCGTAACACTGCAGCAGGT
GCTGCCCGCGCTTGCACCGTCCGAAGAAAAGCGCGGCCTAAAGCGCGAGTCTGGTGACTTGGCACCCACCGTGCAGCTG
ATGGTACCCAAGCGCCAGCGACTGGAAGATGTCTTGGAAAAAATGACCGTGGAACCTGGGCTGGAGCCCGAGGTCCGCG
TGCGGCCAATCAAGCAGGTGGCGCCGGGACTGGGCGTGCAGACCGTGGACGTTCAGATACCCACTACCAGTAGCACCAG
TATTGCCACCGCCACAGAGGGCATGGAGACACAAACGTCCCCGGTTGCCTCAGCGGTGGCGGATGCCGCGGTGCAGGCG
GTCGCTGCGGCCGCGTCCAAGACCTCTACGGAGGTGCAAACGGACCCGTGGATGTTTCGCGTTTCAGCCCCCCGGCGCCC
GCGCCGTTCGAGGAAGTACGGCGCCGCCAGCGCGCTACTGCCCGAATATGCCCTACATCCTTCCATTGCGCCTACCCCCG
GCTATCGTGGCTACACCTACCGCCCCAGAAGACGAGCAACTACCCGACGCCGAACCACCACTGGAACCCGCCGCCGCCG
TCGCCGTCGCCAGCCCGTGCTGGCCCCGATTTCCGTGCGCAGGGTGGCTCGCGAAGGAGGCAGGACCCTGGTGCTGCCA
ACAGCGCGCTACCACCCCAGCATCGTTTAAAAGCCGGTCTTTGTGGTTCTTGCAGATATGGCCCTCACCTGCCGCCTCCGT
TTCCCGGTGCCGGGATTCCGAGGAAGAATGCACCGTAGGAGGGGCATGGCCGGCCACGGCCTGACGGGCGGCATGCGT
CGTGCGCACCACCGGCGGCGGCGCGCGTCGCACCGTCGCATGCGCGGCGGTATCCTGCCCCTCCTTATTCCACTGATCGC
CGCGGCGATTGGCGCCGTGCCCGGAATTGCATCCGTGGCCTTGCAGGCGCAGAGACACTGATTAAAAACAAGTTGCATGT
GGAAAAATCAAAATAAAAAGTCTGGACTCTCACGCTCGCTTGGTCCTGTAACTATTTTGTAGAATGGAAGACATCAACTTT
GCGTCTCTGGCCCCGCGACACGGCTCGCGCCCGTTCATGGGAAACTGGCAAGATATCGGCACCAGCAATATGAGCGGTG
GCGCCTTCAGCTGGGGCTCGCTGTGGAGCGGCATTAAAAATTTCGGTTCCACCGTTAAGAACTATGGCAGCAAGGCCTGG
AACAGCAGCACAGGCCAGATGCTGAGGGATAAGTTGAAAGAGCAAAATTTCCAACAAAAGGTGGTAGATGGCCTGGCCT
CTGGCATTAGCGGGGTGGTGGACCTGGCCAACCAGGCAGTGCAAAATAAGATTAACAGTAAGCTTGATCCCCGCCCTCCC
GTAGAGGAGCCTCCACCGGCCGTGGAGACAGTGTCTCCAGAGGGGCGTGGCGAAAAGCGTCCGCGCCCCGACAGGGAA
GAAACTCTGGTGACGCAAATAGACGAGCCTCCCTCGTACGAGGAGGCACTAAAGCAAGGCCTGCCCACCACCCGTCCCA
TCGCGCCCATGGCTACCGGAGTGCTGGGCCAGCACACACCCGTAACGCTGGACCTGCCTCCCCCCGCCGACACCCAGCA
GAAACCTGTGCTGCCAGGCCCGACCGCCGTTGTTGTAACCCGTCCTAGCCGCGCGTCCCTGCGCCGCGCCGCCAGCGGTC
CGCGATCGTTGCGGCCCGTAGCCAGTGGCAACTGGCAAAGCACACTGAACAGCATCGTGGGTCTGGGGGTGCAATCCCT
GAAGCGCCGACGATGCTTCTGATAGCTAACGTGTCGTATGTGTGTCATGTATGCGTCCATGTCGCCGCCAGAGGAGCTGCT
GAGCCGCCGCGCGCCCGCTTTCCAAGATGGCTACCCCTTCGATGATGCCGCAGTGGTCTTACATGCACATCTCGGGCCAG
GACGCCTCGGAGTACCTGAGCCCCGGGCTGGTGCAGTTTGCCCGCGCCACCGAGACGTACTTCAGCCTGAATAACAAGTT
TAGAAACCCCACGGTGGCGCCTACGCACGACGTGACCACAGACCGGTCCCAGCGTTTGACGCTGCGGTTCATCCCTGTGG
ACCGTGAGGATACTGCGTACTCGTACAAGGCGCGGTTCACCCTAGCTGTGGGTGATAACCGTGTGCTGGACATGGCTTCC
ACGTACTTTGACATCCGCGGCGTGCTGGACAGGGGCCCTACTTTTAAGCCCTACTCTGGCACTGCCTACAACGCCCTGGCT
CCCAAGGGTGCCCCAAATCCTTGCGAATGGGATGAAGCTGCTACTGCTCTTGAAATAAACCTAGAAGAAGAGGACGATGA
CAACGAAGACGAAGTAGACGAGCAAGCTGAGCAGCAAAAAACTCACGTATTTGGGCAGGCGCCTTATTCTGGTATAAATA
TTACAAAGGAGGGTATTCAAATAGGTGTCGAAGGTCAAACACCTAAATATGCCGATAAAACATTTCAACCTGAACCTCAAA
TAGGAGAATCTCAGTGGTACGAAACAGAAATTAATCATGCAGCTGGGAGAGTCCTAAAAAAGACTACCCCAATGAAACCA
TGTTACGGTTCATATGCAAAACCCACAAATGAAAATGGAGGGCAAGGCATTCTTGTAAAGCAACAAAATGGAAAGCTAGA
AAGTCAAGTGGAAATGCAATTTTTCTCAACTACTGAGGCAGCCGCAGGCAATGGTGATAACTTGACTCCTAAAGTGGTATT
GTACAGTGAAGATGTAGATATAGAAACCCCAGACACTCATATTTCTTACATGCCCACTATTAAGGAAGGTAACTCACGAGA
ACTAATGGGCCAACAATCTATGCCCAACAGGCCTAATTACATTGCTTTTAGGGACAATTTTATTGGTCTAATGTATTACAAC
AGCACGGGTAATATGGGTGTTCTGGCGGGCCAAGCATCGCAGTTGAATGCTGTTGTAGATTTGCAAGACAGAAACACAGA
GCTTTCATACCAGCTTTTGCTTGATTCCATTGGTGATAGAACCAGGTACTTTTCTATGTGGAATCAGGCTGTTGACAGCTATG
ATCCAGATGTTAGAATTATTGAAAATCATGGAACTGAAGATGAACTTCCAAATTACTGCTTTCCACTGGGAGGTGTGATTAA
TACAGAGACTCTTACCAAGGTAAAACCTAAAACAGGTCAGGAAAATGGATGGGAAAAAGATGCTACAGAATTTTCAGATA
AAAATGAAATAAGAGTTGGAAATAATTTTGCCATGGAAATCAATCTAAATGCCAACCTGTGGAGAAATTTCCTGTACTCCA
ACATAGCGCTGTATTTGCCCGACAAGCTAAAGTACAGTCCTTCCAACGTAAAAATTTCTGATAACCCAAACACCTACGACT
ACATGAACAAGCGAGTGGTGGCTCCCGGGCTAGTGGACTGCTACATTAACCTTGGAGCACGCTGGTCCCTTGACTATATG
GACAACGTCAACCCATTTAACCACCACCGCAATGCTGGCCTGCGCTACCGCTCAATGTTGCTGGGCAATGGTCGCTATGTG

CCCTTCCACATCCAGGTGCCTCAGAAGTTCTTTGCCATTAAAAACCTCCTTCTCCTGCCGGGCTCATACACCTACGAGTGGA
ACTTCAGGAAGGATGTTAACATGGTTCTGCAGAGCTCCCTAGGAAATGACCTAAGGGTTGACGGAGCCAGCATTAAGTTT
GATAGCATTTGCCTTTACGCCACCTTCTTCCCCATGGCCCACAACACCGCCTCCACGCTTGAGGCCATGCTTAGAAACGAC
ACCAACGACCAGTCCTTTAACGACTATCTCTCCGCCGCCAACATGCTCTACCCTATACCCGCCAACGCTACCAACGTGCCC
ATATCCATCCCCTCCCGCAACTGGGCGGCTTTCCGCGGCTGGGCCTTCACGCGCCTTAAGACTAAGGAAACCCCATCACT
GGGCTCGGGCTACGACCCTTATTACACCTACTCTGGCTCTATACCCTACCTAGATGGAACCTTTTACCTCAACCACACCTTT
AAGAAGGTGGCCATTACCTTTGACTCTTCTGTCAGCTGGCCTGGCAATGACCGCCTGCTTACCCCCAACGAGTTTGAAATT
AAGCGCTCAGTTGACGGGGAGGGTTACAACGTTGCCCAGTGTAACATGACCAAAGACTGGTTCCTGGTACAAATGCTAGC
TAACTATAACATTGGCTACCAGGGCTTCTATATCCCAGAGAGCTACAAGGACCGCATGTACTCCTTCTTTAGAAACTTCCAG
CCCATGAGCCGTCAGGTGGTGGATGATACTAAATACAAGGACTACCAACAGGTGGGCATCCTACACCAACACAACAACTC
TGGATTTGTTGGCTACCTTGCCCCCACCATGCGCGAAGGACAGGCCTACCCTGCTAACTTCCCCTATCCGCTTATAGGCAA
GACCGCAGTTGACAGCATTACCCAGAAAAAGTTTCTTTGCGATCGCACCCTTTGGCGCATCCCATTCTCCAGTAACTTTATG
TCCATGGGCGCACTCACAGACCTGGGCCAAAACCTTCTCTACGCCAACTCCGCCCACGCGCTAGACATGACTTTTGAGGT
GGATCCCATGGACGAGCCCACCCTTCTTTATGTTTTGTTTGAAGTCTTTGACGTGGTCCGTGTGCACCAGCCGCACCGCGG
CGTCATCGAAACCGTGTACCTGCGCACGCCCTTCTCGGCCGGCAACGCCACAACATAAAGAAGCAAGCAACATCAACAA
CAGCTGCCGCCATGGGCTCCAGTGAGCAGGAACTGAAAGCCATTGTCAAAGATCTTGGTTGTGGGCCATATTTTTTGGGCA
CCTATGACAAGCGCTTTCCAGGCTTTGTTTCTCCACACAAGCTCGCCTGCGCCATAGTCAATACGGCCGGTCGCGAGACTG
GGGGCGTACACTGGATGGCCTTTGCCTGGAACCCGCACTCAAAAACATGCTACCTCTTTGAGCCCTTTGGCTTTTCTGACC
AGCGACTCAAGCAGGTTTACCAGTTTGAGTACGAGTCACTCCTGCGCCGTAGCGCCATTGCTTCTTCCCCCGACCGCTGTA
TAACGCTGGAAAAGTCCACCCAAAGCGTACAGGGGCCCAACTCGGCCGCCTGTGGACTATTCTGCTGCATGTTTCTCCAC
GCCTTTGCCAACTGGCCCCAAACTCCCATGGATCACAACCCCACCATGAACCTTATTACCGGGGTACCCAACTCCATGCTC
AACAGTCCCCAGGTACAGCCCACCCTGCGTCGCAACCAGGAACAGCTCTACAGCTTCCTGGAGCGCCACTCGCCCTACTT
CCGCAGCCACAGTGCGCAGATTAGGAGCGCCACTTCTTTTTGTCACTTGAAAAACATGTAAAAATAATGTACTAGAGACAC
TTTCAATAAAGGCAAATGCTTTTATTTGTACACTCTCGGGTGATTATTTACCCCCACCCTTGCCGTCTGCGCCGTTTAAAAAT
CAAAGGGGTTCTGCCGCGCATCGCTATGCGCCACTGGCAGGGACACGTTGCGATACTGGTGTTTAGTGCTCCACTTAAACT
CAGGCACAACCATCCGCGGCAGCTCGGTGAAGTTTTCACTCCACAGGCTGCGCACCATCACCAACGCGTTTAGCAGGTCG
GGCGCCGATATCTTGAAGTCGCAGTTGGGGCCTCCGCCCTGCGCGCGCGAGTTGCGATACACAGGGTTGCAGCACTGGA
ACACTATCAGCGCCGGGTGGTGCACGCTGGCCAGCACGCTCTTGTCGGAGATCAGATCCGCGTCCAGGTCCTCCGCGTTG
CTCAGGGCGAACGGAGTCAACTTTGGTAGCTGCCTTCCCAAAAAGGGCGCGTGCCCAGGCTTTGAGTTGCACTCGCACCG
TAGTGGCATCAAAAGGTGACCGTGCCCGGTCTGGGCGTTAGGATACAGCGCCTGCATAAAAGCCTTGATCTGCTTAAAAG
CCACCTGAGCCTTTGCGCCTTCAGAGAAGAACATGCCGCAAGACTTGCCGGAAAACTGATTGGCCGGACAGGCCGCGTC
GTGCACGCAGCACCTTGCGTCGGTGTTGGAGATCTGCACCACATTTCGGCCCCACCGGTTCTTCACGATCTTGGCCTTGCT
AGACTGCTCCTTCAGCGCGCGCTGCCCGTTTTCGCTCGTCACATCCATTTCAATCACGTGCTCCTTATTTATCATAATGCTTC
CGTGTAGACACTTAAGCTCGCCTTCGATCTCAGCGCAGCGGTGCAGCCACAACGCGCAGCCCGTGGGCTCGTGATGCTTG
TAGGTCACCTCTGCAAACGACTGCAGGTACGCCTGCAGGAATCGCCCCATCATCGTCACAAAGGTCTTGTTGCTGGTGAA
GGTCAGCTGCAACCCGCGGTGCTCCTCGTTCAGCCAGGTCTTGCATACGGCCGCCAGAGCTTCCACTTGGTCAGGCAGTA
GTTTGAAGTTCGCCTTTAGATCGTTATCCACGTGGTACTTGTCCATCAGCGCGCGCGCAGCCTCCATGCCCTTCTCCCACGC
AGACACGATCGGCACACTCAGCGGGTTCATCACCGTAATTTCACTTTCCGCTTCGCTGGGCTCTTCCTCTTCCTCTTGCGTC
CGCATACCACGCGCCACTGGGTCGTCTTCATTCAGCCGCCGCACTGTGCGCTTACCTCCTTTGCCATGCTTGATTAGCACC
GGTGGGTTGCTGAAACCCACCATTTGTAGCGCCACATCTTCTCTTTCTTCCTCGCTGTCCACGATTACCTCTGGTGATGGCG
GGCGCTCGGGCTTGGGAGAAGGGCGCTTCTTTTTCTTCTTGGGCGCAATGGCCAAATCCGCCGCCGAGGTCGATGGCCGC
GGGCTGGGTGTGCGCGGCACCAGCGCGTCTTGTGATGAGTCTTCCTCGTCCTCGGACTCGATACGCCGCCTCATCCGCTTT
TTTGGGGGCGCCCGGGGAGGCGGCGGCGACGGGGACGGGGACGACACGTCCTCCATGGTTGGGGGACGTCGCGCCGCA
CCGCGTCCGCGCTCGGGGGTGGTTTCGCGCTGCTCCTCTTCCCGACTGGCCATTTCCTTCTCCTATAGGCAGAAAAAGATC
ATGGAGTCAGTCGAGAAGAAGGACAGCCTAACCGCCCCCTCTGAGTTCGCCACCACCGCCTCCACCGATGCCGCCAACG
CGCCTACCACCTTCCCCGTCGAGGCACCCCCGCTTGAGGAGGAGGAAGTGATTATCGAGCAGGACCCAGGTTTTGTAAGC
GAAGACGACGAGGACCGCTCAGTACCAACAGAGGATAAAAAGCAAGACCAGGACAACGCAGAGGCAAACGAGGAACA
AGTCGGGCGGGGGGACGAAAGGCATGGCGACTACCTAGATGTGGGAGACGACGTGCTGTTGAAGCATCTGCAGCGCCA
GTGCGCCATTATCTGCGACGCGTTGCAAGAGCGCAGCGATGTGCCCCTCGCCATAGCGGATGTCAGCCTTGCCTACGAAC

GCCACCTATTCTCACCGCGCGTACCCCCCAAACGCCAAGAAAACGGCACATGCGAGCCCAACCCGCGCCTCAACTTCTAC
CCCGTATTTGCCGTGCCAGAGGTGCTTGCCACCTATCACATCTTTTTCCAAAACTGCAAGATACCCCTATCCTGCCGTGCCA
ACCGCAGCCGAGCGGACAAGCAGCTGGCCTTGCGGCAGGGCGCTGTCATACCTGATATCGCCTCGCTCAACGAAGTGCC
AAAAATCTTTGAGGGTCTTGGACGCGACGAGAAGCGCGCGGCAAACGCTCTGCAACAGGAAAACAGCGAAAATGAAAGT
CACTCTGGAGTGTTGGTGGAACTCGAGGGTGACAACGCGCGCCTAGCCGTACTAAAACGCAGCATCGAGGTCACCCACTT
TGCCTACCCGGCACTTAACCTACCCCCCAAGGTCATGAGCACAGTCATGAGTGAGCTGATCGTGCGCCGTGCGCAGCCCC
TGGAGAGGGATGCAAATTTGCAAGAACAAACAGAGGAGGGCCTACCCGCAGTTGGCGACGAGCAGCTAGCGCGCTGGCT
TCAAACGCGCGAGCCTGCCGACTTGGAGGAGCGACGCAAACTAATGATGGCCGCAGTGCTCGTTACCGTGGAGCTTGAGT
GCATGCAGCGGTTCTTTGCTGACCCGGAGATGCAGCGCAAGCTAGAGGAAACATTGCACTACACCTTTCGACAGGGCTAC
GTACGCCAGGCCTGCAAGATCTCCAACGTGGAGCTCTGCAACCTGGTCTCCTACCTTGGAATTTTGCACGAAAACCGCCTT
GGGCAAAACGTGCTTCATTCCACGCTCAAGGGCGAGGCGCGCCGCGACTACGTCCGCGACTGCGTTTACTTATTTCTATGC
TACACCTGGCAGACGGCCATGGGCGTTTGGCAGCAGTGCTTGGAGGAGTGCAACCTCAAGGAGCTGCAGAAACTGCTAA
AGCAAAACTTGAAGGACCTATGGACGGCCTTCAACGAGCGCTCCGTGGCCGCGCACCTGGCGGACATCATTTTCCCCGAA
CGCCTGCTTAAAACCCTGCAACAGGGTCTGCCAGACTTCACCAGTCAAAGCATGTTGCAGAACTTTAGGAACTTTATCCTA
GAGCGCTCAGGAATCTTGCCCGCCACCTGCTGTGCACTTCCTAGCGACTTTGTGCCCATTAAGTACCGCGAATGCCCTCCG
CCGCTTTGGGGCCACTGCTACCTTCTGCAGCTAGCCAACTACCTTGCCTACCACTCTGACATAATGGAAGACGTGAGCGGT
GACGGTCTACTGGAGTGTCACTGTCGCTGCAACCTATGCACCCCGCACCGCTCCCTGGTTTGCAATTCGCAGCTGCTTAAC
GAAAGTCAAATTATCGGTACCTTTGAGCTGCAGGGTCCCTCGCCTGACGAAAAGTCCGCGGCTCCGGGGTTGAAACTCAC
TCCGGGGCTGTGGACGTCGGCTTACCTTCGCAAATTTGTACCTGAGGACTACCACGCCCACGAGATTAGGTTCTACGAAGA
CCAATCCCGCCCGCCTAATGCGGAGCTTACCGCCTGCGTCATTACCCAGGGCCACATTCTTGGCCAATTGCAAGCCATCAA
CAAAGCCCGCCAAGAGTTTCTGCTACGAAAGGGACGGGGGGTTTACTTGGACCCCCAGTCCGGCGAGGAGCTCAACCCA
ATCCCCCCGCCGCCGCAGCCCTATCAGCAGCAGCCGCGGGCCCTTGCTTCCCAGGATGGCACCCAAAAAGAAGCTGCAG
CTGCCGCCGCCACCCACGGACGAGGAGGAATACTGGGACAGTCAGGCAGAGGAGGTTTTGGACGAGGAGGAGGAGGAC
ATGATGGAAGACTGGGAGAGCCTAGACGAGGAAGCTTCCGAGGTCGAAGAGGTGTCAGACGAAACACCGTCACCCTCGG
TCGCATTCCCCTCGCCGGCGCCCCAGAAATCGGCAACCGGTTCCAGCATGGCTACAACCTCCGCTCCTCAGGCGCCGCCG
GCACTGCCCGTTCGCCGACCCAACCGTAGATGGGACACCACTGGAACCAGGGCCGGTAAGTCCAAGCAGCCGCCGCCGT
TAGCCCAAGAGCAACAACAGCGCCAAGGCTACCGCTCATGGCGCGGGCACAAGAACGCCATAGTTGCTTGCTTGCAAGA
CTGTGGGGGCAACATCTCCTTCGCCCGCCGCTTTCTTCTCTACCATCACGGCGTGGCCTTCCCCCGTAACATCCTGCATTAC
TACCGTCATCTCTACAGCCCATACTGCACCGGCGGCAGCGGCAGCAACAGCAGCGGCCACACAGAAGCAAAGGCGACCG
GATAGCAAGACTCTGACAAAGCCCAAGAAATCCACAGCGGCGGCAGCAGCAGGAGGAGGAGCGCTGCGTCTGGCGCCC
AACGAACCCGTATCGACCCGCGAGCTTAGAAACAGGATTTTTCCCACTCTGTATGCTATATTTCAACAGAGCAGGGGCCAA
GAACAAGAGCTGAAAATAAAAAACAGGTCTCTGCGATCCCTCACCCGCAGCTGCCTGTATCACAAAAGCGAAGATCAGCT
TCGGCGCACGCTGGAAGACGCGGAGGCTCTCTTCAGTAAATACTGCGCGCTGACTCTTAAGGACTAGTTTCGCGCCCTTTC
TCAAATTTAAGCGCGAAAACTACGTCATCTCCAGCGGCCACACCCGGCGCCAGCACCTGTTGTCAGCGCCATTATGAGCA
AGGAAATTCCCACGCCCTACATGTGGAGTTACCAGCCACAAATGGGACTTGCGGCTGGAGCTGCCCAAGACTACTCAACC
CGAATAAACTACATGAGCGCGGGACCCCACATGATATCCCGGGTCAACGGAATACGCGCCCACCGAAACCGAATTCTCCT
GGAACAGGCGGCTATTACCACCACACCTCGTAATAACCTTAATCCCCGTAGTTGGCCCGCTGCCCTGGTGTACCAGGAAA
GTCCCGCTCCCACCACTGTGGTACTTCCCAGAGACGCCCAGGCCGAAGTTCAGATGACTAACTCAGGGGCGCAGCTTGCG
GGCGGCTTTCGTCACAGGGTGCGGTCGCCCGGGCAGGGTATAACTCACCTGACAATCAGAGGGCGAGGTATTCAGCTCA
ACGACGAGTCGGTGAGCTCCTCGCTTGGTCTCCGTCCGGACGGGACATTTCAGATCGGCGGCGCCGGCCGCTCTTCATTC
ACGCCTCGTCAGGCAATCCTAACTCTGCAGACCTCGTCCTCTGAGCCGCGCTCTGGAGGCATTGGAACTCTGCAATTTATT
GAGGAGTTTGTGCCATCGGTCTACTTTAACCCCTTCTCGGGACCTCCCGGCCACTATCCGGATCAATTTATTCCTAACTTTG
ACGCGGTAAAGGACTCGGCGGACGGCTACGACTGAATGTTAAGTGGAGAGGCAGAGCAACTGCGCCTGAAACACCTGGT
CCACTGTCGCCGCCACAAGTGCTTTGCCCGCGACTCCGGTGAGTTTTGCTACTTTGAATTGCCCGAGGATCATATCGAGGG
CCCGGCGCACGGCGTCCGGCTTACCGCCCAGGGAGAGCTTGCCCGTAGCCTGATTCGGGAGTTTACCCAGCGCCCCCTG
CTAGTTGAGCGGGACAGGGGACCCTGTGTTCTCACTGTGATTTGCAACTGTCCTAACCCTGGATTACATCAAGATCTTTGTT
GCCATCTCTGTGCTGAGTATAATAAATACAGAAATTAAAATATACTGGGGCTCCTATCGCCATCCTGTAAACGCCACCGTCT
TCACCCGCCCAAGCAAACCAAGGCGAACCTTACCTGGTACTTTTAACATCTCTCCCTCTGTGATTTACAACAGTTTCAACCC
AGACGGAGTGAGTCTACGAGAGAACCTCTCCGAGCTCAGCTACTCCATCAGAAAAAACACCACCCTCCTTACCTGCCGGG

AACGTACGAGTGCGTCACCGGCCGCTGCACCACACCTACCGCCTGACCGTAAACCAGACTTTTTCCGGACAGACCTCAAT
AACTCTGTTTACCAGAACAGGAGGTGAGCTTAGAAAACCCTTAGGGTATTAGGCCAAAGGCGCAGCTACTGTGGGGTTTA
TGAACAATTCAAGCAACTCTACGGGCTATTCTAATTCAGGTTTCTCTAGAAATGGACGGAATTATTACAGAGCAGCGCCTG
CTAGAAAGACGCAGGGCAGCGGCCGAGCAACAGCGCATGAATCAAGAGCTCCAAGACATGGTTAACTTGCACCAGTGCA
AAAGGGGTATCTTTTGTCTGGTAAAGCAGGCCAAAGTCACCTACGACAGTAATACCACCGGACACCGCCTTAGCTACAAG
TTGCCAACCAAGCGTCAGAAATTGGTGGTCATGGTGGGAGAAAAGCCCATTACCATAACTCAGCACTCGGTAGAAACCGA
AGGCTGCATTCACTCACCTTGTCAAGGACCTGAGGATCTCTGCACCCTTATTAAGACCCTGTGCGGTCTCAAAGATCTTATT
CCCTTTAACTAATAAAAAAAAATAATAAAGCATCACTTACTTAAAATCAGTTAGCAAATTTCTGTCCAGTTTATTCAGCAGC
ACCTCCTTGCCCTCCTCCCAGCTCTGGTATTGCAGCTTCCTCCTGGCTGCAAACTTTCTCCACAATCTAAATGGAATGTCAG
TTTCCTCCTGTTCCTGTCCATCCGCACCCACTATCTTCATGTTGTTGCAGATGAAGCGCGCAAGACCGTCTGAAGATACCTT
CAACCCCGTGTATCCATATGACACGGAAACCGGTCCTCCAACTGTGCCTTTTCTTACTCCTCCCTTTGTATCCCCCAATGGG
TTTCAAGAGAGTCCCCCTGGGGTACTCTCTTTGCGCCTATCCGAACCTCTAGTTACCTCCAATGGCATGCTTGCGCTCAAAA
TGGGCAACGGCCTCTCTCTGGACGAGGCCGGCAACCTTACCTCCCAAAATGTAACCACTGTGAGCCCACCTCTCAAAAAA
ACCAAGTCAAACATAAACCTGGAAATATCTGCACCCCTCACAGTTACCTCAGAAGCCCTAACTGTGGCTGCCGCCGCACCT
CTAATGGTCGCGGGCAACACACTCACCATGCAATCACAGGCCCCGCTAACCGTGCACGACTCCAAACTTAGCATTGCCAC
CCAAGGACCCCTCACAGTGTCAGAAGGAAAGCTAGCCCTGCAAACATCAGGCCCCCTCACCACCACCGATAGCAGTACC
CTTACTATCACTGCCTCACCCCCTCTAACTACTGCCACTGGTAGCTTGGGCATTGACTTGAAAGAGCCCATTTATACACAAA
ATGGAAAACTAGGACTAAAGTACGGGGCTCCTTTGCATGTAACAGACGACCTAAACACTTTGACCGTAGCAACTGGTCCA
GGTGTGACTATTAATAATACTTCCTTGCAAACTAAAGTTACTGGAGCCTTGGGTTTTGATTCACAAGGCAATATGCAACTTA
ATGTAGCAGGAGGACTAAGGATTGATTCTCAAAACAGACGCCTTATACTTGATGTTAGTTATCCGTTTGATGCTCAAAACCA
ACTAAATCTAAGACTAGGACAGGGCCCTCTTTTTATAAACTCAGCCCACAACTTGGATATTAACTACAACAAAGGCCTTTAC
TTGTTTACAGCTTCAAACAATTCCAAAAAGCTTGAGGTTAACCTAAGCACTGCCAAGGGGTTGATGTTTGACGCTACAGCC
ATAGCCATTAATGCAGGAGATGGGCTTGAATTTGGTTCACCTAATGCACCAAACACAAATCCCCTCAAAACAAAAATTGGC
CATGGCCTAGAATTTGATTCAAACAAGGCTATGGTTCCTAAACTAGGAACTGGCCTTAGTTTTGACAGCACAGGTGCCATT
ACAGTAGGAAACAAAAATAATGATAAGCTAACTTTGTGGACCACACCAGCTCCATCTCCTAACTGTAGACTAAATGCAGAG
AAAGATGCTAAACTCACTTTGGTCTTAACAAAATGTGGCAGTCAAATACTTGCTACAGTTTCAGTTTTGGCTGTTAAAGGCA
GTTTGGCTCCAATATCTGGAACAGTTCAAAGTGCTCATCTTATTATAAGATTTGACGAAAATGGAGTGCTACTAAACAATTC
CTTCCTGGACCCAGAATATTGGAACTTTAGAAATGGAGATCTTACTGAAGGCACAGCCTATACAAACGCTGTTGGATTTAT
GCCTAACCTATCAGCTTATCCAAAATCTCACGGTAAAACTGCCAAAAGTAACATTGTCAGTCAAGTTTACTTAAACGGAGA
CAAAACTAAACCTGTAACACTAACCATTACACTAAACGGTACACAGGAAACAGGAGACACAACTCCAAGTGCATACTCTA
TGTCATTTTCATGGGACTGGTCTGGCCACAACTACATTAATGAAATATTTGCCACATCCTCTTACACTTTTTCATACATTGCC
CAAGAATAAAGAATCGTTTGTGTTATGTTTCAACGTGTTTATTTTTCAATTGCAGAAAATTTCGAATCATTTTTCATTCAGTAG
TATAGCCCCACCACCACATAGCTTATACAGATCACCGTACCTTAATCAAACTCACAGAACCCTAGTATTCAACCTGCCACCT
CCCTCCCAACACACAGAGTACACAGTCCTTTCTCCCCGGCTGGCCTTAAAAAGCATCATATCATGGGTAACAGACATATTC
TTAGGTGTTATATTCCACACGGTTTCCTGTCGAGCCAAACGCTCATCAGTGATATTAATAAACTCCCCGGGCAGCTCACTTA
AGTTCATGTCGCTGTCCAGCTGCTGAGCCACAGGCTGCTGTCCAACTTGCGGTTGCTTAACGGGCGGCGAAGGAGAAGTC
CACGCCTACATGGGGGTAGAGTCATAATCGTGCATCAGGATAGGGCGGTGGTGCTGCAGCAGCGCGCGAATAAACTGCT
GCCGCCGCCGCTCCGTCCTGCAGGAATACAACATGGCAGTGGTCTCCTCAGCGATGATTCGCACCGCCCGCAGCATAAGG
CGCCTTGTCCTCCGGGCACAGCAGCGCACCCTGATCTCACTTAAATCAGCACAGTAACTGCAGCACAGCACCACAATATT
GTTCAAAATCCCACAGTGCAAGGCGCTGTATCCAAAGCTCATGGCGGGGACCACAGAACCCACGTGGCCATCATACCACA
AGCGCAGGTAGATTAAGTGGCGACCCCTCATAAACACGCTGGACATAAACATTACCTCTTTTGGCATGTTGTAATTCACCA
CCTCCCGGTACCATATAAACCTCTGATTAAACATGGCGCCATCCACCACCATCCTAAACCAGCTGGCCAAAACCTGCCCGC
CGGCTATACACTGCAGGGAACCGGGACTGGAACAATGACAGTGGAGAGCCCAGGACTCGTAACCATGGATCATCATGCT
CGTCATGATATCAATGTTGGCACAACACAGGCACACGTGCATACACTTCCTCAGGATTACAAGCTCCTCCCGCGTTAGAAC
CATATCCCAGGGAACAACCCATTCCTGAATCAGCGTAAATCCCACACTGCAGGGAAGACCTCGCACGTAACTCACGTTGT
GCATTGTCAAAGTGTTACATTCGGGCAGCAGCGGATGATCCTCCAGTATGGTAGCGCGGGTTTCTGTCTCAAAAGGAGGTA
GACGATCCCTACTGTACGGAGTGCGCCGAGACAACCGAGATCGTGTTGGTCGTAGTGTCATGCCAAATGGAACGCCGGAC
GTAGTCATATTTCCTGAAGCAAAACCAGGTGCGGGCGTGACAAACAGATCTGCGTCTCCGGTCTCGCCGCTTAGATCGCTC
TGTGTAGTAGTTGTAGTATATCCACTCTCTCAAAGCATCCAGGCGCCCCCTGGCTTCGGGTTCTATGTAAACTCCTTCATGC

GCCGCTGCCCTGATAACATCCACCACCGCAGAATAAGCCACACCCAGCCAACCTACACATTCGTTCTGCGAGTCACACAC
GGGAGGAGCGGGAAGAGCTGGAAGAACCATGTTTTTTTTTTTATTCCAAAAGATTATCCAAAACCTCAAAATGAAGATCTA
TTAAGTGAACGCGCTCCCCTCCGGTGGCGTGGTCAAACTCTACAGCCAAAGAACAGATAATGGCATTTGTAAGATGTTGCA
CAATGGCTTCCAAAAGGCAAACGGCCCTCACGTCCAAGTGGACGTAAAGGCTAAACCCTTCAGGGTGAATCTCCTCTATA
AACATTCCAGCACCTTCAACCATGCCCAAATAATTCTCATCTCGCCACCTTCTCAATATATCTCTAAGCAAATCCCGAATATT
AAGTCCGGCCATTGTAAAAATCTGCTCCAGAGCGCCCTCCACCTTCAGCCTCAAGCAGCGAATCATGATTGCAAAAATTCA
GGTTCCTCACAGACCTGTATAAGATTCAAAAGCGGAACATTAACAAAAATACCGCGATCCCGTAGGTCCCTTCGCAGGGC
CAGCTGAACATAATCGTGCAGGTCTGCACGGACCAGCGCGGCCACTTCCCCGCCAGGAACCATGACAAAAGAACCCACA
CTGATTATGACACGCATACTCGGAGCTATGCTAACCAGCGTAGCCCCGATGTAAGCTTGTTGCATGGGCGGCGATATAAAA
TGCAAGGTGCTGCTCAAAAAATCAGGCAAAGCCTCGCGCAAAAAAGAAAGCACATCGTAGTCATGCTCATGCAGATAAAG
GCAGGTAAGCTCCGGAACCACCACAGAAAAAGACACCATTTTTCTCTCAAACATGTCTGCGGGTTTCTGCATAAACACAAA
ATAAAATAACAAAAAAACATTTAAACATTAGAAGCCTGTCTTACAACAGGAAAAACAACCCTTATAAGCATAAGACGGACT
ACGGCCATGCCGGCGTGACCGTAAAAAAACTGGTCACCGTGATTAAAAAGCACCACCGACAGCTCCTCGGTCATGTCCGG
AGTCATAATGTAAGACTCGGTAAACACATCAGGTTGATTCACATCGGTCAGTGCTAAAAAGCGACCGAAATAGCCCGGGG
GAATACATACCCGCAGGCGTAGAGACAACATTACAGCCCCCATAGGAGGTATAACAAAATTAATAGGAGAGAAAAACAC
ATAAACACCTGAAAAACCCTCCTGCCTAGGCAAAATAGCACCCTCCCGCTCCAGAACAACATACAGCGCTTCCACAGCGG
CAGCCATAACAGTCAGCCTTACCAGTAAAAAAGAAAACCTATTAAAAAAACACCACTCGACACGGCACCAGCTCAATCAG
TCACAGTGTAAAAAAGGGCCAAGTGCAGAGCGAGTATATATAGGACTAAAAAATGACGTAACGGTTAAAGTCCACAAAAA
ACACCCAGAAAACCGCACGCGAACCTACGCCCAGAAACGAAAGCCAAAAAACCCACAACTTCCTCAAATCGTCACTTCC
GTTTTCCCACGTTACGTCACTTCCCATTTTAAGAAAACTACAATTCCCAACACATACAAGTTACTCCGCCCTAAAACCTACG
TCACCCGCCCCGTTCCCACGCCCCGCGCCACGTCACAAACTCCACCCCCTCATTATCATATTGGCTTCAATCCAAAATAAG
GTATATTATTGATGATGTTAATTAATTTAAATCCGCATGCGATATCGAGCTCTCCCGGGAATTCGGATCTGCGACGCGAGGC
TGGATGGCCTTCCCCATTATGATTCTTCTCGCTTCCGGCGGCATCGGGATGCCCGCGTTGCAGGCCATGCTGTCCAGGCAG
GTAGATGACGACCATCAGGGACAGCTTCAAGGCCAGCAAAAGGCCAGGAACCGTAAAAAGGCCGCGTTGCTGGCGTTTT
TCCATAGGCTCCGCCCCCCTGACGAGCATCACAAAAATCGACGCTCAAGTCAGAGGTGGCGAAACCCGACAGGACTATA
AAGATACCAGGCGTTTCCCCCTGGAAGCTCCCTCGTGCGCTCTCCTGTTCCGACCCTGCCGCTTACCGGATACCTGTCCGC
CTTTCTCCCTTCGGGAAGCGTGGCGCTTTCTCATAGCTCACGCTGTAGGTATCTCAGTTCGGTGTAGGTCGTTCGCTCCAAG
CTGGGCTGTGTGCACGAACCCCCCGTTCAGCCCGACCGCTGCGCCTTATCCGGTAACTATCGTCTTGAGTCCAACCCGGTA
AGACACGACTTATCGCCACTGGCAGCAGCCACTGGTAACAGGATTAGCAGAGCGAGGTATGTAGGCGGTGCTACAGAGT
TCTTGAAGTGGTGGCCTAACTACGGCTACACTAGAAGGACAGTATTTGGTATCTGCGCTCTGCTGAAGCCAGTTACCTTCG
GAAAAAGAGTTGGTAGCTCTTGATCCGGCAAACAAACCACCGCTGGTAGCGGTGGTTTTTTTGTTTGCAAGCAGCAGATTA
CGCGCAGAAAAAAAGGATCTCAAGAAGATCCTTTGATCTTTTCTACGGGGTCTGACGCTCAGTGGAACGAAAACTCACGTT
AAGGGATTTTGGTCATGAGATTATCAAAAAGGATCTTCACCTAGATCCTTTTAAATCAATCTAAAGTATATATGAGTAAACTT
GGTCTGACAGTTACCAATGCTTAATCAGTGAGGCACCTATCTCAGCGATCTGTCTATTTCGTTCATCCATAGTTGCCTGACT
CCCCGTCGTGTAGATAACTACGATACGGGAGGGCTTACCATCTGGCCCCAGTGCTGCAATGATACCGCGAGACCCACGCT
CACCGGCTCCAGATTTATCAGCAATAAACCAGCCAGCCGGAAGGGCCGAGCGCAGAAGTGGTCCTGCAACTTTATCCGCC
TCCATCCAGTCTATTAATTGTTGCCGGGAAGCTAGAGTAAGTAGTTCGCCAGTTAATAGTTTGCGCAACGTTGTTGCCATTG
CTACAGGCATCGTGGTGTCACGCTCGTCGTTTGGTATGGCTTCATTCAGCTCCGGTTCCCAACGATCAAGGCGAGTTACAT
GATCCCCCATGTTGTGCAAAAAAGCGGTTAGCTCCTTCGGTCCTCCGATCGTTGTCAGAAGTAAGTTGGCCGCAGTGTTAT
CACTCATGGTTATGGCAGCACTGCATAATTCTCTTACTGTCATGCCATCCGTAAGATGCTTTTCTGTGACTGGTGAGTACTC
AACCAAGTCATTCTGAGAATAGTGTATGCGGCGACCGAGTTGCTCTTGCCCGGCGTCAACACGGGATAATACCGCGCCAC
ATAGCAGAACTTTAAAAGTGCTCATCATTGGAAAACGTTCTTCGGGGCGAAAACTCTCAAGGATCTTACCGCTGTTGAGAT
CCAGTTCGATGTAACCCACTCGTGCACCCAACTGATCTTCAGCATCTTTTACTTTCACCAGCGTTTCTGGGTGAGCAAAAAC
AGGAAGGCAAAATGCCGCAAAAAAGGGAATAAGGGCGACACGGAAATGTTGAATACTCATACTCTTCCTTTTTCAATATTA
TTGAAGCATTTATCAGGGTTATTGTCTCATGAGCGGATACATATTTGAATGTATTTAGAAAAATAAACAAATAGGGGTTCCG
CGCACATTTCCCCGAAAAGTGCCACCTGACGTCTAAGAAACCATTATTATCATGACATTAACCTATAAAAATAGGCGTATC
ACGAGGCCCTTTCGTCTTCAAGAATTGGATCCGAATTCCCGGGAGAGCTCGATATCGCATGCGGATTTAAATTAATTAA

pAV-gB-3×GS-EGFP (ADV-gB)

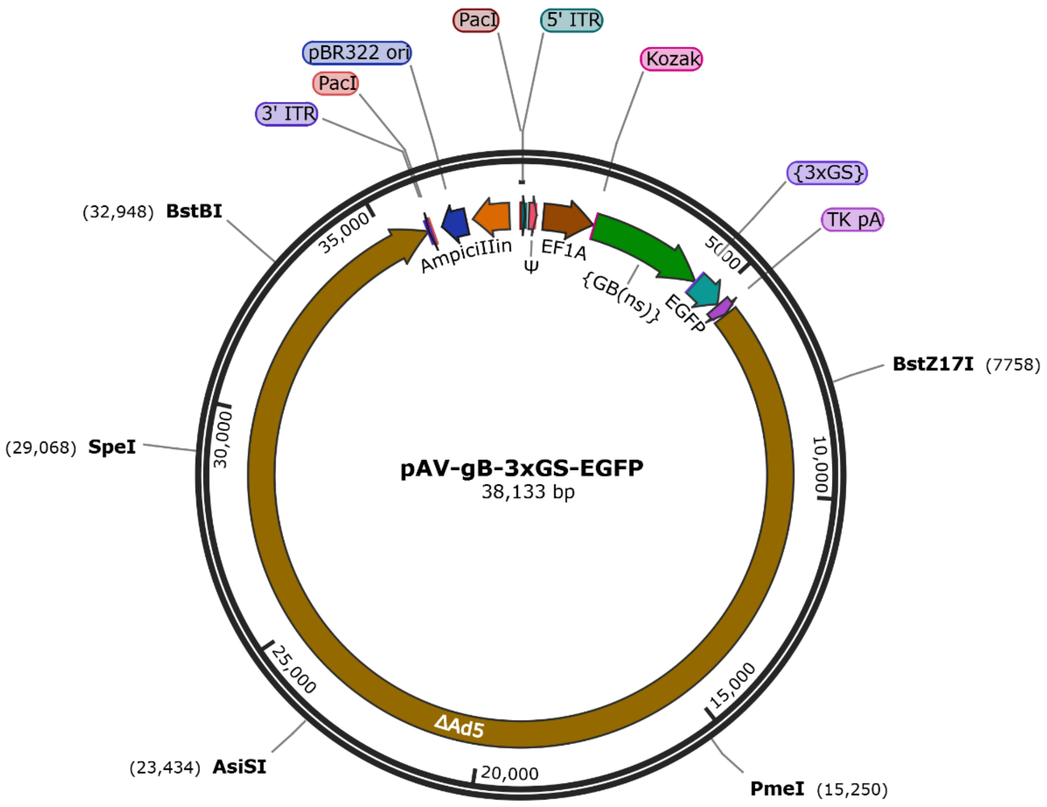

CATCATCAATAATATACCTTATTTTGGATTGAAGCCAATATGATAATGAGGGGGTGGAGTTTGTGACGTGGCGCGGGGCGT
GGGAACGGGGCGGGTGACGTAGTAGTGTGGCGGAAGTGTGATGTTGCAAGTGTGGCGGAACACATGTAAGCGACGGATG
TGGCAAAAGTGACGTTTTTGGTGTGCGCCGGTGTACACAGGAAGTGACAATTTTCGCGCGGTTTTAGGCGGATGTTGTAGT
AAATTTGGGCGTAACCGAGTAAGATTTGGCCATTTTCGCGGGAAAACTGAATAAGAGGAAGTGAAATCTGAATAATTTTGT
GTTACTCATAGCGCGTAATATTTGTCTAGGGCCGCGGGGACTTTGACCGTTTACGTGGAGACTCGCCCAGGTGTTTTTCTCA
GGTGTTTTCCGCGTTCCGGGTCAAAGTTGGCGTTTTATTATTATAGTCAGTCGAAGCTTGGATCCGGTACCTCTAGAATTCTC
GAGCGGCCGCTAGCGACATCGATCAACTTTGTATAGAAAAGTTGGGCTCCGGTGCCCGTCAGTGGGCAGAGCGCACATCG
CCCACAGTCCCCGAGAAGTTGGGGGGAGGGGTCGGCAATTGAACCGGTGCCTAGAGAAGGTGGCGCGGGGTAAACTGG
GAAAGTGATGTCGTGTACTGGCTCCGCCTTTTTCCCGAGGGTGGGGGAGAACCGTATATAAGTGCAGTAGTCGCCGTGAA
CGTTCTTTTTCGCAACGGGTTTGCCGCCAGAACACAGGTAAGTGCCGTGTGTGGTTCCCGCGGGCCTGGCCTCTTTACGGG
TTATGGCCCTTGCGTGCCTTGAATTACTTCCACCTGGCTGCAGTACGTGATTCTTGATCCCGAGCTTCGGGTTGGAAGTGGG
TGGGAGAGTTCGAGGCCTTGCGCTTAAGGAGCCCCTTCGCCTCGTGCTTGAGTTGAGGCCTGGCCTGGGCGCTGGGGCCG
CCGCGTGCGAATCTGGTGGCACCTTCGCGCCTGTCTCGCTGCTTTCGATAAGTCTCTAGCCATTTAAAATTTTTGATGACCT
GCTGCGACGCTTTTTTTCTGGCAAGATAGTCTTGTAAATGCGGGCCAAGATCTGCACACTGGTATTTCGGTTTTTGGGGCCG
CGGGCGGCGACGGGGCCCGTGCGTCCCAGCGCACATGTTCGGCGAGGCGGGGCCTGCGAGCGCGGCCACCGAGAATCG
GACGGGGGTAGTCTCAAGCTGGCCGGCCTGCTCTGGTGCCTGGTCTCGCGCCGCCGTGTATCGCCCCGCCCTGGGCGGC
AAGGCTGGCCCGGTCGGCACCAGTTGCGTGAGCGGAAAGATGGCCGCTTCCCGGCCCTGCTGCAGGGAGCTCAAAATGG
AGGACGCGGCGCTCGGGAGAGCGGGCGGGTGAGTCACCCACACAAAGGAAAAGGGCCTTTCCGTCCTCAGCCGTCGCTT
CATGTGACTCCACGGAGTACCGGGCGCCGTCCAGGCACCTCGATTAGTTCTCGAGCTTTTGGAGTACGTCGTCTTTAGGTT
GGGGGGAGGGGTTTTATGCGATGGAGTTTCCCCACACTGAGTGGGTGGAGACTGAAGTTAGGCCAGCTTGGCACTTGATG
TAATTCTCCTTGGAATTTGCCCTTTTTGAGTTTGGATCTTGGTTCATTCTCAAGCCTCAGACAGTGGTTCAAAGTTTTTTTCTT
CCATTTCAGGTGTCGTGACAAGTTTGTACAAAAAAGCAGGCTGCCACCATGCGCCAGGGCGCCCCCGCGCGGGGGCGCC
GGTGGTTCGTCGTATGGGCGCTCTTGGGGTTGACGCTGGGGGTCCTGGTGGCGTCGGCGGCTCCGAGTTCCCCCGGCACG
CCTGGGGTCGCGGCCGCGACCCAGGCGGCGAACGGGGGCCCTGCCACTCCGGCGCCGCCCGCCCCTGGCGCCCCCCCA
ACGGGGGACCCGAAACCGAAGAAGAACAAAAAACCGAAACCCCCAAAGCCGCCGCGCCCCGCCGGCGACAACGCGAC

CGTCGCCGCGGGCCACGCCACCCTGCGCGAGCACCTGCGGGACATCAAGGCGGAGAACACCGATGCAAACTTTTACGTG
TGCCCACCCCCCACGGGCGCCACGGTGGTGCAGTTCGAGCAGCCGCGCCGCTGCCCGACCCGGCCCGAGGGTCAGAACT
ACACGGAGGGCATCGCGGTGGTCTTCAAGGAGAACATCGCCCCGTACAAGTTCAAGGCCACCATGTACTACAAAGACGTC
ACCGTTTCGCAGGTGTGGTTCGGCCACCGCTACTCCCAGTTTATGGGGATCTTTGAGGACCGCGCCCCCGTCCCCTTCGAG
GAGGTGATCGACAAGATCAACGCCAAGGGGGTCTGTCGGTCCACGGCCAAGTACGTGCGCAACAACCTGGAGACCACCG
CGTTTCACCGGGACGACCACGAGACCGACATGGAGCTGAAACCGGCCAACGCCGCGACCCGCACGAGCCGGGGCTGGC
ACACCACCGACCTCAAGTACAACCCCTCGCGGGTGGAGGCGTTCCACCGGTACGGGACGACGGTAAACTGCATCGTCGA
GGAGGTGGACGCGCGCTCGGTGTACCCGTACGACGAGTTTGTGTTGGCGACTGGCGACTTTGTGTACATGTCCCCGTTTTA
CGGCTACCGGGAGGGGTCGCACACCGAACACACCAGCTACGCCGCCGACCGCTTCAAGCAGGTCGACGGCTTCTACGCG
CGCGACCTCACCACCAAGGCCCGGGCCACGGCGCCGACCACCCGGAACCTGCTCACGACCCCCAAGTTCACCGTGGCCT
GGGACTGGGTGCCAAAGCGCCCGTCGGTCTGCACCATGACCAAGTGGCAGGAGGTGGACGAGATGCTGCGCTCCGAGTA
CGGCGGCTCCTTCCGATTCTCTTCCGACGCCATATCCACCACCTTCACCACCAACCTGACCGAGTACCCGCTCTCGCGCGT
GGACCTGGGGGACTGCATCGGCAAGGACGCCCGCGACGCCATGGACCGCATCTTCGCCCGCAGGTACAACGCGACGCA
CATCAAGGTGGGCCAGCCGCAGTACTACCTGGCCAATGGGGGCTTTCTGATCGCGTACCAGCCCCTTCTCAGCAACACGC
TCGCGGAGCTGTACGTGCGGGAACACCTCCGCGAGCAGAGCCGCAAGCCCCCAAACCCCACGCCCCCGCCGCCCGGGG
CCAGCGCCAACGCGTCCGTGGAGCGCATCAAGACCACCTCCTCCATCGAGTTCGCCAGGCTGCAGTTTACGTACAACCAC
ATACAGCGCCATGTCAACGATATGTTGGGCCGCGTTGCCATCGCGTGGTGCGAGCTGCAGAATCACGAGCTGACCCTGTG
GAACGAGGCCCGCAAGCTGAACCCCAACGCCATCGCCTCGGCCACCGTGGGCCGGCGGGTGAGCGCGCGGATGCTCGG
CGACGTGATGGCCGTCTCCACGTGCGTGCCGGTCGCCGCGGACAACGTGATCGTCCAAAACTCGATGCGCATCAGCTCGC
GGCCCGGGGCCTGCTACAGCCGCCCCCTGGTCAGCTTTCGGTACGAAGACCAGGGCCCGTTGGTCGAGGGGCAGCTGGG
GGAGAACAACGAGCTGCGGCTGACGCGCGATGCGATCGAGCCGTGCACCGTGGGACACCGGCGCTACTTCACCTTCGGT
GGGGGCTACGTGTACTTCGAGGAGTACGCGTACTCCCACCAGCTGAGCCGCGCCGACATCACCACCGTCAGCACCTTCAT
CGACCTCAACATCACCATGCTGGAGGATCACGAGTTTGTCCCCCTGGAGGTGTACACCCGCCACGAGATCAAGGACAGCG
GCCTGCTGGACTACACGGAGGTCCAGCGCCGCAACCAGCTGCACGACCTGCGCTTCGCCGACATCGACACGGTCATCCA
CGCCGACGCCAACGCCGCCATGTTTGCGGGCCTGGGCGCGTTCTTCGAGGGGATGGGCGACCTGGGGCGCGCGGTCGGC
AAGGTGGTGATGGGCATCGTGGGCGGCGTGGTATCGGCCGTGTCGGGCGTGTCCTCCTTCATGTCCAACCCCTTTGGGGC
GCTGGCCGTGGGTCTGTTGGTCCTGGCCGGCCTGGCGGCGGCCTTCTTCGCCTTTCGCTACGTCATGCGGCTGCAGAGCA
ACCCCATGAAGGCCCTGTACCCGCTAACCACCAAGGAGCTCAAGAACCCCACCAACCCGGACGCGTCCGGGGAGGGCG
AGGAGGGCGGCGACTTTGACGAGGCCAAGCTAGCCGAGGCCCGGGAGATGATACGGTACATGGCCCTGGTGTCTGCCAT
GGAGCGCACGGAACACAAGGCCAAGAAGAAGGGCACGAGCGCGCTGCTCAGCGCCAAGGTCACCGACATGGTCATGCG
CAAGCGCCGCAACACCAACTACACCCAAGTTCCCAACAAAGACGGTGACGCCGACGAGGACGACCTGGGCAGCGGCAG
CGGCAGCATGGTGAGCAAGGGCGAGGAGCTGTTCACCGGGGTGGTGCCCATCCTGGTCGAGCTGGACGGCGACGTAAAC
GGCCACAAGTTCAGCGTGTCCGGCGAGGGCGAGGGCGATGCCACCTACGGCAAGCTGACCCTGAAGTTCATCTGCACCA
CCGGCAAGCTGCCCGTGCCCTGGCCCACCCTCGTGACCACCCTGACCTACGGCGTGCAGTGCTTCAGCCGCTACCCCGAC
CACATGAAGCAGCACGACTTCTTCAAGTCCGCCATGCCCGAAGGCTACGTCCAGGAGCGCACCATCTTCTTCAAGGACGA
CGGCAACTACAAGACCCGCGCCGAGGTGAAGTTCGAGGGCGACACCCTGGTGAACCGCATCGAGCTGAAGGGCATCGA
CTTCAAGGAGGACGGCAACATCCTGGGGCACAAGCTGGAGTACAACTACAACAGCCACAACGTCTATATCATGGCCGAC
AAGCAGAAGAACGGCATCAAGGTGAACTTCAAGATCCGCCACAACATCGAGGACGGCAGCGTGCAGCTCGCCGACCACT
ACCAGCAGAACACCCCCATCGGCGACGGCCCCGTGCTGCTGCCCGACAACCACTACCTGAGCACCCAGTCCGCCCTGAG
CAAAGACCCCAACGAGAAGCGCGATCACATGGTCCTGCTGGAGTTCGTGACCGCCGCCGGGATCACTCTCGGCATGGAC
GAGCTGTACAAGTAAACCCAGCTTTCTTGTACAAAGTGGTGGGGGAGGCTAACTGAAACACGGAAGGAGACAATACCGG
AAGGAACCCGCGCTATGACGGCAATAAAAAGACAGAATAAAACGCACGGGTGTTGGGTCGTTTGTTCATAAACGCGGGG
TTCGGTCCCAGGGCTGGCACTCTGTCGATACCCCACCGAGACCCCATTGGGGCCAATACGCCCGCGTTTCTTCCTTTTCCC
CACCCCACCCCCCAAGTTCGGGTGAAGGCCCAGGGCTCGCAGCCAACGTCGGGGCGGCAGGCCCTGCCATAGCGATCGA
TTCGACAGATCACTGAAATGTGTGGGCGTGGCTTAAGGGTGGGAAAGAATATATAAGGTGGGGGTCTTATGTAGTTTTGTA
TCTGTTTTGCAGCAGCCGCCGCCGCCATGAGCACCAACTCGTTTGATGGAAGCATTGTGAGCTCATATTTGACAACGCGCA
TGCCCCCATGGGCCGGGGTGCGTCAGAATGTGATGGGCTCCAGCATTGATGGTCGCCCCGTCCTGCCCGCAAACTCTACT
ACCTTGACCTACGAGACCGTGTCTGGAACGCCGTTGGAGACTGCAGCCTCCGCCGCCGCTTCAGCCGCTGCAGCCACCGC
CCGCGGGATTGTGACTGACTTTGCTTTCCTGAGCCCGCTTGCAAGCAGTGCAGCTTCCCGTTCATCCGCCCGCGATGACAA

GTTGACGGCTCTTTTGGCACAATTGGATTCTTTGACCCGGGAACTTAATGTCGTTTCTCAGCAGCTGTTGGATCTGCGCCAG
CAGGTTTCTGCCCTGAAGGCTTCCTCCCCTCCCAATGCGGTTTAAAACATAAATAAAAAACCAGACTCTGTTTGGATTTGGA
TCAAGCAAGTGTCTTGCTGTCTTTATTTAGGGGTTTTGCGCGCGCGGTAGGCCCGGGACCAGCGGTCTCGGTCGTTGAGGG
TCCTGTGTATTTTTTCCAGGACGTGGTAAAGGTGACTCTGGATGTTCAGATACATGGGCATAAGCCCGTCTCTGGGGTGGA
GGTAGCACCACTGCAGAGCTTCATGCTGCGGGGTGGTGTTGTAGATGATCCAGTCGTAGCAGGAGCGCTGGGCGTGGTGC
CTAAAAATGTCTTTCAGTAGCAAGCTGATTGCCAGGGGCAGGCCCTTGGTGTAAGTGTTTACAAAGCGGTTAAGCTGGGAT
GGGTGCATACGTGGGGATATGAGATGCATCTTGGACTGTATTTTTAGGTTGGCTATGTTCCCAGCCATATCCCTCCGGGGA
TTCATGTTGTGCAGAACCACCAGCACAGTGTATCCGGTGCACTTGGGAAATTTGTCATGTAGCTTAGAAGGAAATGCGTGG
AAGAACTTGGAGACGCCCTTGTGACCTCCAAGATTTTCCATGCATTCGTCCATAATGATGGCAATGGGCCCACGGGCGGC
GGCCTGGGCGAAGATATTTCTGGGATCACTAACGTCATAGTTGTGTTCCAGGATGAGATCGTCATAGGCCATTTTTACAAA
GCGCGGGCGGAGGGTGCCAGACTGCGGTATAATGGTTCCATCCGGCCCAGGGGCGTAGTTACCCTCACAGATTTGCATTT
CCCACGCTTTGAGTTCAGATGGGGGGATCATGTCTACCTGCGGGGCGATGAAGAAAACGGTTTCCGGGGTAGGGGAGAT
CAGCTGGGAAGAAAGCAGGTTCCTGAGCAGCTGCGACTTACCGCAGCCGGTGGGCCCGTAAATCACACCTATTACCGGCT
GCAACTGGTAGTTAAGAGAGCTGCAGCTGCCGTCATCCCTGAGCAGGGGGGCCACTTCGTTAAGCATGTCCCTGACTCGC
ATGTTTTCCCTGACCAAATCCGCCAGAAGGCGCTCGCCGCCCAGCGATAGCAGTTCTTGCAAGGAAGCAAAGTTTTTCAAC
GGTTTGAGACCGTCCGCCGTAGGCATGCTTTTGAGCGTTTGACCAAGCAGTTCCAGGCGGTCCCACAGCTCGGTCACCTG
CTCTACGGCATCTCGATCCAGCATATCTCCTCGTTTCGCGGGTTGGGGCGGCTTTCGCTGTACGGCAGTAGTCGGTGCTCG
TCCAGACGGGCCAGGGTCATGTCTTTCCACGGGCGCAGGGTCCTCGTCAGCGTAGTCTGGGTCACGGTGAAGGGGTGCG
CTCCGGGCTGCGCGCTGGCCAGGGTGCGCTTGAGGCTGGTCCTGCTGGTGCTGAAGCGCTGCCGGTCTTCGCCCTGCGCG
TCGGCCAGGTAGCATTTGACCATGGTGTCATAGTCCAGCCCCTCCGCGGCGTGGCCCTTGGCGCGCAGCTTGCCCTTGGA
GGAGGCGCCGCACGAGGGGCAGTGCAGACTTTTGAGGGCGTAGAGCTTGGGCGCGAGAAATACCGATTCCGGGGAGTA
GGCATCCGCGCCGCAGGCCCCGCAGACGGTCTCGCATTCCACGAGCCAGGTGAGCTCTGGCCGTTCGGGGTCAAAAACC
AGGTTTCCCCCATGCTTTTTGATGCGTTTCTTACCTCTGGTTTCCATGAGCCGGTGTCCACGCTCGGTGACGAAAAGGCTGT
CCGTGTCCCCGTATACAGACTTGAGAGGCCTGTCCTCGAGCGGTGTTCCGCGGTCCTCCTCGTATAGAAACTCGGACCACT
CTGAGACAAAGGCTCGCGTCCAGGCCAGCACGAAGGAGGCTAAGTGGGAGGGGTAGCGGTCGTTGTCCACTAGGGGGT
CCACTCGCTCCAGGGTGTGAAGACACATGTCGCCCTCTTCGGCATCAAGGAAGGTGATTGGTTTGTAGGTGTAGGCCACG
TGACCGGGTGTTCCTGAAGGGGGGCTATAAAAGGGGGTGGGGGCGCGTTCGTCCTCACTCTCTTCCGCATCGCTGTCTGC
GAGGGCCAGCTGTTGGGGTGAGTACTCCCTCTGAAAAGCGGGCATGACTTCTGCGCTAAGATTGTCAGTTTCCAAAAACG
AGGAGGATTTGATATTCACCTGGCCCGCGGTGATGCCTTTGAGGGTGGCCGCATCCATCTGGTCAGAAAAGACAATCTTTT
TGTTGTCAAGCTTGGTGGCAAACGACCCGTAGAGGGCGTTGGACAGCAACTTGGCGATGGAGCGCAGGGTTTGGTTTTTG
TCGCGATCGGCGCGCTCCTTGGCCGCGATGTTTAGCTGCACGTATTCGCGCGCAACGCACCGCCATTCGGGAAAGACGGT
GGTGCGCTCGTCGGGCACCAGGTGCACGCGCCAACCGCGGTTGTGCAGGGTGACAAGGTCAACGCTGGTGGCTACCTCT
CCGCGTAGGCGCTCGTTGGTCCAGCAGAGGCGGCCGCCCTTGCGCGAGCAGAATGGCGGTAGGGGGTCTAGCTGCGTCT
CGTCCGGGGGGTCTGCGTCCACGGTAAAGACCCCGGGCAGCAGGCGCGCGTCGAAGTAGTCTATCTTGCATCCTTGCAAG
TCTAGCGCCTGCTGCCATGCGCGGGCGGCAAGCGCGCGCTCGTATGGGTTGAGTGGGGGACCCCATGGCATGGGGTGGG
TGAGCGCGGAGGCGTACATGCCGCAAATGTCGTAAACGTAGAGGGGCTCTCTGAGTATTCCAAGATATGTAGGGTAGCAT
CTTCCACCGCGGATGCTGGCGCGCACGTAATCGTATAGTTCGTGCGAGGGAGCGAGGAGGTCGGGACCGAGGTTGCTAC
GGGCGGGCTGCTCTGCTCGGAAGACTATCTGCCTGAAGATGGCATGTGAGTTGGATGATATGGTTGGACGCTGGAAGACG
TTGAAGCTGGCGTCTGTGAGACCTACCGCGTCACGCACGAAGGAGGCGTAGGAGTCGCGCAGCTTGTTGACCAGCTCGG
CGGTGACCTGCACGTCTAGGGCGCAGTAGTCCAGGGTTTCCTTGATGATGTCATACTTATCCTGTCCCTTTTTTTTCCACAG
CTCGCGGTTGAGGACAAACTCTTCGCGGTCTTTCCAGTACTCTTGGATCGGAAACCCGTCGGCCTCCGAACGGTAAGAGC
CTAGCATGTAGAACTGGTTGACGGCCTGGTAGGCGCAGCATCCCTTTTCTACGGGTAGCGCGTATGCCTGCGCGGCCTTCC
GGAGCGAGGTGTGGGTGAGCGCAAAGGTGTCCCTGACCATGACTTTGAGGTACTGGTATTTGAAGTCAGTGTCGTCGCAT
CCGCCCTGCTCCCAGAGCAAAAAGTCCGTGCGCTTTTTGGAACGCGGATTTGGCAGGGCGAAGGTGACATCGTTGAAGAG
TATCTTTCCCGCGCGAGGCATAAAGTTGCGTGTGATGCGGAAGGGTCCCGGCACCTCGGAACGGTTGTTAATTACCTGGG
CGGCGAGCACGATCTCGTCAAAGCCGTTGATGTTGTGGCCCACAATGTAAAGTTCCAAGAAGCGCGGGATGCCCTTGATG
GAAGGCAATTTTTTAAGTTCCTCGTAGGTGAGCTCTTCAGGGGAGCTGAGCCCGTGCTCTGAAAGGGCCCAGTCTGCAAG
ATGAGGGTTGGAAGCGACGAATGAGCTCCACAGGTCACGGGCCATTAGCATTTGCAGGTGGTCGCGAAAGGTCCTAAACT
GGCGACCTATGGCCATTTTTTCTGGGGTGATGCAGTAGAAGGTAAGCGGGTCTTGTTCCCAGCGGTCCCATCCAAGGTTCG

CGGCTAGGTCTCGCGCGGCAGTCACTAGAGGCTCATCTCCGCCGAACTTCATGACCAGCATGAAGGGCACGAGCTGCTTC
CCAAAGGCCCCCATCCAAGTATAGGTCTCTACATCGTAGGTGACAAAGAGACGCTCGGTGCGAGGATGCGAGCCGATCG
GGAAGAACTGGATCTCCCGCCACCAATTGGAGGAGTGGCTATTGATGTGGTGAAAGTAGAAGTCCCTGCGACGGGCCGA
ACACTCGTGCTGGCTTTTGTAAAAACGTGCGCAGTACTGGCAGCGGTGCACGGGCTGTACATCCTGCACGAGGTTGACCT
GACGACCGCGCACAAGGAAGCAGAGTGGGAATTTGAGCCCCTCGCCTGGCGGGTTTGGCTGGTGGTCTTCTACTTCGGCT
GCTTGTCCTTGACCGTCTGGCTGCTCGAGGGGAGTTACGGTGGATCGGACCACCACGCCGCGCGAGCCCAAAGTCCAGAT
GTCCGCGCGCGGCGGTCGGAGCTTGATGACAACATCGCGCAGATGGGAGCTGTCCATGGTCTGGAGCTCCCGCGGCGTC
AGGTCAGGCGGGAGCTCCTGCAGGTTTACCTCGCATAGACGGGTCAGGGCGCGGGCTAGATCCAGGTGATACCTAATTTC
CAGGGGCTGGTTGGTGGCGGCGTCGATGGCTTGCAAGAGGCCGCATCCCCGCGGCGCGACTACGGTACCGCGCGGCGG
GCGGTGGGCCGCGGGGGTGTCCTTGGATGATGCATCTAAAAGCGGTGACGCGGGCGAGCCCCCGGAGGTAGGGGGGC
TCCGGACCCGCCGGGAGAGGGGGCAGGGGCACGTCGGCGCCGCGCGCGGGCAGGAGCTGGTGCTGCGCGCGTAGGTT
GCTGGCGAACGCGACGACGCGGCGGTTGATCTCCTGAATCTGGCGCCTCTGCGTGAAGACGACGGGCCCGGTGAGCTTG
AACCTGAAAGAGAGTTCGACAGAATCAATTTCGGTGTCGTTGACGGCGGCCTGGCGCAAAATCTCCTGCACGTCTCCTGA
GTTGTCTTGATAGGCGATCTCGGCCATGAACTGCTCGATCTCTTCCTCCTGGAGATCTCCGCGTCCGGCTCGCTCCACGGT
GGCGGCGAGGTCGTTGGAAATGCGGGCCATGAGCTGCGAGAAGGCGTTGAGGCCTCCCTCGTTCCAGACGCGGCTGTAG
ACCACGCCCCCTTCGGCATCGCGGGCGCGCATGACCACCTGCGCGAGATTGAGCTCCACGTGCCGGGCGAAGACGGCGT
AGTTTCGCAGGCGCTGAAAGAGGTAGTTGAGGGTGGTGGCGGTGTGTTCTGCCACGAAGAAGTACATAACCCAGCGTCGC
AACGTGGATTCGTTGATATCCCCCAAGGCCTCAAGGCGCTCCATGGCCTCGTAGAAGTCCACGGCGAAGTTGAAAAACTG
GGAGTTGCGCGCCGACACGGTTAACTCCTCCTCCAGAAGACGGATGAGCTCGGCGACAGTGTCGCGCACCTCGCGCTCA
AAGGCTACAGGGGCCTCTTCTTCTTCTTCAATCTCCTCTTCCATAAGGGCCTCCCCTTCTTCTTCTTCTGGCGGCGGTGGGG
GAGGGGGGACACGGCGGCGACGACGGCGCACCGGGAGGCGGTCGACAAAGCGCTCGATCATCTCCCCGCGGCGACGG
CGCATGGTCTCGGTGACGGCGCGGCCGTTCTCGCGGGGGCGCAGTTGGAAGACGCCGCCCGTCATGTCCCGGTTATGGG
TTGGCGGGGGGCTGCCATGCGGCAGGGATACGGCGCTAACGATGCATCTCAACAATTGTTGTGTAGGTACTCCGCCGCCG
AGGGACCTGAGCGAGTCCGCATCGACCGGATCGGAAAACCTCTCGAGAAAGGCGTCTAACCAGTCACAGTCGCAAGGTA
GGCTGAGCACCGTGGCGGGCGGCAGCGGGCGGCGGTCGGGGTTGTTTCTGGCGGAGGTGCTGCTGATGATGTAATTAAA
GTAGGCGGTCTTGAGACGGCGGATGGTCGACAGAAGCACCATGTCCTTGGGTCCGGCCTGCTGAATGCGCAGGCGGTCG
GCCATGCCCCAGGCTTCGTTTTGACATCGGCGCAGGTCTTTGTAGTAGTCTTGCATGAGCCTTTCTACCGGCACTTCTTCTT
CTCCTTCCTCTTGTCCTGCATCTCTTGCATCTATCGCTGCGGCGGCGGCGGAGTTTGGCCGTAGGTGGCGCCCTCTTCCTCC
CATGCGTGTGACCCCGAAGCCCCTCATCGGCTGAAGCAGGGCTAGGTCGGCGACAACGCGCTCGGCTAATATGGCCTGCT
GCACCTGCGTGAGGGTAGACTGGAAGTCATCCATGTCCACAAAGCGGTGGTATGCGCCCGTGTTGATGGTGTAAGTGCAG
TTGGCCATAACGGACCAGTTAACGGTCTGGTGACCCGGCTGCGAGAGCTCGGTGTACCTGAGACGCGAGTAAGCCCTCGA
GTCAAATACGTAGTCGTTGCAAGTCCGCACCAGGTACTGGTATCCCACCAAAAAGTGCGGCGGCGGCTGGCGGTAGAGG
GGCCAGCGTAGGGTGGCCGGGGCTCCGGGGGCGAGATCTTCCAACATAAGGCGATGATATCCGTAGATGTACCTGGACA
TCCAGGTGATGCCGGCGGCGGTGGTGGAGGCGCGCGGAAAGTCGCGGACGCGGTTCCAGATGTTGCGCAGCGGCAAAA
AGTGCTCCATGGTCGGGACGCTCTGGCCGGTCAGGCGCGCGCAATCGTTGACGCTCTAGACCGTGCAAAAGGAGAGCCT
GTAAGCGGGCACTCTTCCGTGGTCTGGTGGATAAATTCGCAAGGGTATCATGGCGGACGACCGGGGTTCGAGCCCCGTAT
CCGGCCGTCCGCCGTGATCCATGCGGTTACCGCCCGCGTGTCGAACCCAGGTGTGCGACGTCAGACAACGGGGGAGTGC
TCCTTTTGGCTTCCTTCCAGGCGCGGCGGCTGCTGCGCTAGCTTTTTTGGCCACTGGCCGCGCGCAGCGTAAGCGGTTAGG
CTGGAAAGCGAAAGCATTAAGTGGCTCGCTCCCTGTAGCCGGAGGGTTATTTTCCAAGGGTTGAGTCGCGGGACCCCCGG
TTCGAGTCTCGGACCGGCCGGACTGCGGCGAACGGGGGTTTGCCTCCCCGTCATGCAAGACCCCGCTTGCAAATTCCTCC
GGAAACAGGGACGAGCCCCTTTTTTGCTTTTCCCAGATGCATCCGGTGCTGCGGCAGATGCGCCCCCCTCCTCAGCAGCG
GCAAGAGCAAGAGCAGCGGCAGACATGCAGGGCACCCTCCCCTCCTCCTACCGCGTCAGGAGGGGCGACATCCGCGGTT
GACGCGGCAGCAGATGGTGATTACGAACCCCCGCGGCGCCGGGCCCGGCACTACCTGGACTTGGAGGAGGGCGAGGGC
CTGGCGCGGCTAGGAGCGCCCTCTCCTGAGCGGCACCCAAGGGTGCAGCTGAAGCGTGATACGCGTGAGGCGTACGTGC
CGCGGCAGAACCTGTTTCGCGACCGCGAGGGAGAGGAGCCCGAGGAGATGCGGGATCGAAAGTTCCACGCAGGGCGCG
AGCTGCGGCATGGCCTGAATCGCGAGCGGTTGCTGCGCGAGGAGGACTTTGAGCCCGACGCGCGAACCGGGATTAGTCC
CGCGCGCGCACACGTGGCGGCCGCCGACCTGGTAACCGCATACGAGCAGACGGTGAACCAGGAGATTAACTTTCAAAAA
AGCTTTAACAACCACGTGCGTACGCTTGTGGCGCGCGAGGAGGTGGCTATAGGACTGATGCATCTGTGGGACTTTGTAAG
CGCGCTGGAGCAAAACCCAAATAGCAAGCCGCTCATGGCGCAGCTGTTCCTTATAGTGCAGCACAGCAGGGACAACGAG

GCATTCAGGGATGCGCTGCTAAACATAGTAGAGCCCGAGGGCCGCTGGCTGCTCGATTTGATAAACATCCTGCAGAGCAT
AGTGGTGCAGGAGCGCAGCTTGAGCCTGGCTGACAAGGTGGCCGCCATCAACTATTCCATGCTTAGCCTGGGCAAGTTTT
ACGCCCGCAAGATATACCATACCCCTTACGTTCCCATAGACAAGGAGGTAAAGATCGAGGGGTTCTACATGCGCATGGCG
CTGAAGGTGCTTACCTTGAGCGACGACCTGGGCGTTTATCGCAACGAGCGCATCCACAAGGCCGTGAGCGTGAGCCGGC
GGCGCGAGCTCAGCGACCGCGAGCTGATGCACAGCCTGCAAAGGGCCCTGGCTGGCACGGGCAGCGGCGATAGAGAGG
CCGAGTCCTACTTTGACGCGGGCGCTGACCTGCGCTGGGCCCCAAGCCGACGCGCCCTGGAGGCAGCTGGGGCCGGACC
TGGGCTGGCGGTGGCACCCGCGCGCGCTGGCAACGTCGGCGGCGTGGAGGAATATGACGAGGACGATGAGTACGAGCC
AGAGGACGGCGAGTACTAAGCGGTGATGTTTCTGATCAGATGATGCAAGACGCAACGGACCCGGCGGTGCGGGCGGCGC
TGCAGAGCCAGCCGTCCGGCCTTAACTCCACGGACGACTGGCGCCAGGTCATGGACCGCATCATGTCGCTGACTGCGCG
CAATCCTGACGCGTTCCGGCAGCAGCCGCAGGCCAACCGGCTCTCCGCAATTCTGGAAGCGGTGGTCCCGGCGCGCGCA
AACCCCACGCACGAGAAGGTGCTGGCGATCGTAAACGCGCTGGCCGAAAACAGGGCCATCCGGCCCGACGAGGCCGGC
CTGGTCTACGACGCGCTGCTTCAGCGCGTGGCTCGTTACAACAGCGGCAACGTGCAGACCAACCTGGACCGGCTGGTGG
GGGATGTGCGCGAGGCCGTGGCGCAGCGTGAGCGCGCGCAGCAGCAGGGCAACCTGGGCTCCATGGTTGCACTAAACG
CCTTCCTGAGTACACAGCCCGCCAACGTGCCGCGGGGACAGGAGGACTACACCAACTTTGTGAGCGCACTGCGGCTAAT
GGTGACTGAGACACCGCAAAGTGAGGTGTACCAGTCTGGGCCAGACTATTTTTTCCAGACCAGTAGACAAGGCCTGCAGA
CCGTAAACCTGAGCCAGGCTTTCAAAAACTTGCAGGGGCTGTGGGGGGTGCGGGCTCCCACAGGCGACCGCGCGACCGT
GTCTAGCTTGCTGACGCCCAACTCGCGCCTGTTGCTGCTGCTAATAGCGCCCTTCACGGACAGTGGCAGCGTGTCCCGGG
ACACATACCTAGGTCACTTGCTGACACTGTACCGCGAGGCCATAGGTCAGGCGCATGTGGACGAGCATACTTTCCAGGAG
ATTACAAGTGTCAGCCGCGCGCTGGGGCAGGAGGACACGGGCAGCCTGGAGGCAACCCTAAACTACCTGCTGACCAACC
GGCGGCAGAAGATCCCCTCGTTGCACAGTTTAAACAGCGAGGAGGAGCGCATTTTGCGCTACGTGCAGCAGAGCGTGAG
CCTTAACCTGATGCGCGACGGGGTAACGCCCAGCGTGGCGCTGGACATGACCGCGCGCAACATGGAACCGGGCATGTAT
GCCTCAAACCGGCCGTTTATCAACCGCCTAATGGACTACTTGCATCGCGCGGCCGCCGTGAACCCCGAGTATTTCACCAAT
GCCATCTTGAACCCGCACTGGCTACCGCCCCCTGGTTTCTACACCGGGGGATTCGAGGTGCCCGAGGGTAACGATGGATT
CCTCTGGGACGACATAGACGACAGCGTGTTTTCCCCGCAACCGCAGACCCTGCTAGAGTTGCAACAGCGCGAGCAGGCA
GAGGCGGCGCTGCGAAAGGAAAGCTTCCGCAGGCCAAGCAGCTTGTCCGATCTAGGCGCTGCGGCCCCGCGGTCAGATG
CTAGTAGCCCATTTCCAAGCTTGATAGGGTCTCTTACCAGCACTCGCACCACCCGCCCGCGCCTGCTGGGCGAGGAGGAG
TACCTAAACAACTCGCTGCTGCAGCCGCAGCGCGAAAAAAACCTGCCTCCGGCATTTCCCAACAACGGGATAGAGAGCCT
AGTGGACAAGATGAGTAGATGGAAGACGTACGCGCAGGAGCACAGGGACGTGCCAGGCCCGCGCCCGCCCACCCGTCG
TCAAAGGCACGACCGTCAGCGGGGTCTGGTGTGGGAGGACGATGACTCGGCAGACGACAGCAGCGTCCTGGATTTGGGA
GGGAGTGGCAACCCGTTTGCGCACCTTCGCCCCAGGCTGGGGAGAATGTTTTAAAAAAAAAAAAAGCATGATGCAAAATA
AAAAACTCACCAAGGCCATGGCACCGAGCGTTGGTTTTCTTGTATTCCCCTTAGTATGCGGCGCGCGGCGATGTATGAGGA
AGGTCCTCCTCCCTCCTACGAGAGTGTGGTGAGCGCGGCGCCAGTGGCGGCGGCGCTGGGTTCTCCCTTCGATGCTCCCC
TGGACCCGCCGTTTGTGCCTCCGCGGTACCTGCGGCCTACCGGGGGGAGAAACAGCATCCGTTACTCTGAGTTGGCACCC
CTATTCGACACCACCCGTGTGTACCTGGTGGACAACAAGTCAACGGATGTGGCATCCCTGAACTACCAGAACGACCACAG
CAACTTTCTGACCACGGTCATTCAAAACAATGACTACAGCCCGGGGGAGGCAAGCACACAGACCATCAATCTTGACGACC
GGTCGCACTGGGGCGGCGACCTGAAAACCATCCTGCATACCAACATGCCAAATGTGAACGAGTTCATGTTTACCAATAAG
TTTAAGGCGCGGGTGATGGTGTCGCGCTTGCCTACTAAGGACAATCAGGTGGAGCTGAAATACGAGTGGGTGGAGTTCAC
GCTGCCCGAGGGCAACTACTCCGAGACCATGACCATAGACCTTATGAACAACGCGATCGTGGAGCACTACTTGAAAGTGG
GCAGACAGAACGGGGTTCTGGAAAGCGACATCGGGGTAAAGTTTGACACCCGCAACTTCAGACTGGGGTTTGACCCCGTC
ACTGGTCTTGTCATGCCTGGGGTATATACAAACGAAGCCTTCCATCCAGACATCATTTTGCTGCCAGGATGCGGGGTGGAC
TTCACCCACAGCCGCCTGAGCAACTTGTTGGGCATCCGCAAGCGGCAACCCTTCCAGGAGGGCTTTAGGATCACCTACGA
TGATCTGGAGGGTGGTAACATTCCCGCACTGTTGGATGTGGACGCCTACCAGGCGAGCTTGAAAGATGACACCGAACAGG
GCGGGGGTGGCGCAGGCGGCAGCAACAGCAGTGGCAGCGGCGCGGAAGAGAACTCCAACGCGGCAGCCGCGGCAATG
CAGCCGGTGGAGGACATGAACGATCATGCCATTCGCGGCGACACCTTTGCCACACGGGCTGAGGAGAAGCGCGCTGAGG
CCGAAGCAGCGGCCGAAGCTGCCGCCCCCGCTGCGCAACCCGAGGTCGAGAAGCCTCAGAAGAAACCGGTGATCAAAC
CCCTGACAGAGGACAGCAAGAAACGCAGTTACAACCTAATAAGCAATGACAGCACCTTCACCCAGTACCGCAGCTGGTA
CCTTGCATACAACTACGGCGACCCTCAGACCGGAATCCGCTCATGGACCCTGCTTTGCACTCCTGACGTAACCTGCGGCTC
GGAGCAGGTCTACTGGTCGTTGCCAGACATGATGCAAGACCCCGTGACCTTCCGCTCCACGCGCCAGATCAGCAACTTTC
CGGTGGTGGGCGCCGAGCTGTTGCCCGTGCACTCCAAGAGCTTCTACAACGACCAGGCCGTCTACTCCCAACTCATCCGC

CAGTTTACCTCTCTGACCCACGTGTTCAATCGCTTTCCCGAGAACCAGATTTTGGCGCGCCCGCCAGCCCCCACCATCACC
ACCGTCAGTGAAAACGTTCCTGCTCTCACAGATCACGGGACGCTACCGCTGCGCAACAGCATCGGAGGAGTCCAGCGAG
TGACCATTACTGACGCCAGACGCCGCACCTGCCCCTACGTTTACAAGGCCCTGGGCATAGTCTCGCCGCGCGTCCTATCG
AGCCGCACTTTTTGAGCAAGCATGTCCATCCTTATATCGCCCAGCAATAACACAGGCTGGGGCCTGCGCTTCCCAAGCAAG
ATGTTTGGCGGGGCCAAGAAGCGCTCCGACCAACACCCAGTGCGCGTGCGCGGGCACTACCGCGCGCCCTGGGGCGCG
CACAAACGCGGCCGCACTGGGCGCACCACCGTCGATGACGCCATCGACGCGGTGGTGGAGGAGGCGCGCAACTACACG
CCCACGCCGCCACCAGTGTCCACAGTGGACGCGGCCATTCAGACCGTGGTGCGCGGAGCCCGGCGCTATGCTAAAATGA
AGAGACGGCGGAGGCGCGTAGCACGTCGCCACCGCCGCCGACCCGGCACTGCCGCCCAACGCGCGGCGGCGGCCCTGC
TTAACCGCGCACGTCGCACCGGCCGACGGGCGGCCATGCGGGCCGCTCGAAGGCTGGCCGCGGGTATTGTCACTGTGCC
CCCCAGGTCCAGGCGACGAGCGGCCGCCGCAGCAGCCGCGGCCATTAGTGCTATGACTCAGGGTCGCAGGGGCAACGT
GTATTGGGTGCGCGACTCGGTTAGCGGCCTGCGCGTGCCCGTGCGCACCCGCCCCCCGCGCAACTAGATTGCAAGAAAA
AACTACTTAGACTCGTACTGTTGTATGTATCCAGCGGCGGCGGCGCGCAACGAAGCTATGTCCAAGCGCAAAATCAAAGA
AGAGATGCTCCAGGTCATCGCGCCGGAGATCTATGGCCCCCCGAAGAAGGAAGAGCAGGATTACAAGCCCCGAAAGCTA
AAGCGGGTCAAAAAGAAAAAGAAAGATGATGATGATGAACTTGACGACGAGGTGGAACTGCTGCACGCTACCGCGCCCA
GGCGACGGGTACAGTGGAAAGGTCGACGCGTAAAACGTGTTTTGCGACCCGGCACCACCGTAGTCTTTACGCCCGGTGA
GCGCTCCACCCGCACCTACAAGCGCGTGTATGATGAGGTGTACGGCGACGAGGACCTGCTTGAGCAGGCCAACGAGCGC
CTCGGGGAGTTTGCCTACGGAAAGCGGCATAAGGACATGCTGGCGTTGCCGCTGGACGAGGGCAACCCAACACCTAGCC
TAAAGCCCGTAACACTGCAGCAGGTGCTGCCCGCGCTTGCACCGTCCGAAGAAAAGCGCGGCCTAAAGCGCGAGTCTGG
TGACTTGGCACCCACCGTGCAGCTGATGGTACCCAAGCGCCAGCGACTGGAAGATGTCTTGGAAAAAATGACCGTGGAAC
CTGGGCTGGAGCCCGAGGTCCGCGTGCGGCCAATCAAGCAGGTGGCGCCGGGACTGGGCGTGCAGACCGTGGACGTTC
AGATACCCACTACCAGTAGCACCAGTATTGCCACCGCCACAGAGGGCATGGAGACACAAACGTCCCCGGTTGCCTCAGC
GGTGGCGGATGCCGCGGTGCAGGCGGTCGCTGCGGCCGCGTCCAAGACCTCTACGGAGGTGCAAACGGACCCGTGGAT
GTTTCGCGTTTCAGCCCCCCGGCGCCCGCGCCGTTCGAGGAAGTACGGCGCCGCCAGCGCGCTACTGCCCGAATATGCCC
TACATCCTTCCATTGCGCCTACCCCCGGCTATCGTGGCTACACCTACCGCCCCAGAAGACGAGCAACTACCCGACGCCGA
ACCACCACTGGAACCCGCCGCCGCCGTCGCCGTCGCCAGCCCGTGCTGGCCCCGATTTCCGTGCGCAGGGTGGCTCGCG
AAGGAGGCAGGACCCTGGTGCTGCCAACAGCGCGCTACCACCCCAGCATCGTTTAAAAGCCGGTCTTTGTGGTTCTTGCA
GATATGGCCCTCACCTGCCGCCTCCGTTTCCCGGTGCCGGGATTCCGAGGAAGAATGCACCGTAGGAGGGGCATGGCCG
GCCACGGCCTGACGGGCGGCATGCGTCGTGCGCACCACCGGCGGCGGCGCGCGTCGCACCGTCGCATGCGCGGCGGTA
TCCTGCCCCTCCTTATTCCACTGATCGCCGCGGCGATTGGCGCCGTGCCCGGAATTGCATCCGTGGCCTTGCAGGCGCAG
AGACACTGATTAAAAACAAGTTGCATGTGGAAAAATCAAAATAAAAAGTCTGGACTCTCACGCTCGCTTGGTCCTGTAACT
ATTTTGTAGAATGGAAGACATCAACTTTGCGTCTCTGGCCCCGCGACACGGCTCGCGCCCGTTCATGGGAAACTGGCAAG
ATATCGGCACCAGCAATATGAGCGGTGGCGCCTTCAGCTGGGGCTCGCTGTGGAGCGGCATTAAAAATTTCGGTTCCACC
GTTAAGAACTATGGCAGCAAGGCCTGGAACAGCAGCACAGGCCAGATGCTGAGGGATAAGTTGAAAGAGCAAAATTTCC
AACAAAAGGTGGTAGATGGCCTGGCCTCTGGCATTAGCGGGGTGGTGGACCTGGCCAACCAGGCAGTGCAAAATAAGAT
TAACAGTAAGCTTGATCCCCGCCCTCCCGTAGAGGAGCCTCCACCGGCCGTGGAGACAGTGTCTCCAGAGGGGCGTGGC
GAAAAGCGTCCGCGCCCCGACAGGGAAGAAACTCTGGTGACGCAAATAGACGAGCCTCCCTCGTACGAGGAGGCACTAA
AGCAAGGCCTGCCCACCACCCGTCCCATCGCGCCCATGGCTACCGGAGTGCTGGGCCAGCACACACCCGTAACGCTGGA
CCTGCCTCCCCCCGCCGACACCCAGCAGAAACCTGTGCTGCCAGGCCCGACCGCCGTTGTTGTAACCCGTCCTAGCCGCG
CGTCCCTGCGCCGCGCCGCCAGCGGTCCGCGATCGTTGCGGCCCGTAGCCAGTGGCAACTGGCAAAGCACACTGAACAG
CATCGTGGGTCTGGGGGTGCAATCCCTGAAGCGCCGACGATGCTTCTGATAGCTAACGTGTCGTATGTGTGTCATGTATGC
GTCCATGTCGCCGCCAGAGGAGCTGCTGAGCCGCCGCGCGCCCGCTTTCCAAGATGGCTACCCCTTCGATGATGCCGCAG
TGGTCTTACATGCACATCTCGGGCCAGGACGCCTCGGAGTACCTGAGCCCCGGGCTGGTGCAGTTTGCCCGCGCCACCGA
GACGTACTTCAGCCTGAATAACAAGTTTAGAAACCCCACGGTGGCGCCTACGCACGACGTGACCACAGACCGGTCCCAGC
GTTTGACGCTGCGGTTCATCCCTGTGGACCGTGAGGATACTGCGTACTCGTACAAGGCGCGGTTCACCCTAGCTGTGGGTG
ATAACCGTGTGCTGGACATGGCTTCCACGTACTTTGACATCCGCGGCGTGCTGGACAGGGGCCCTACTTTTAAGCCCTACT
CTGGCACTGCCTACAACGCCCTGGCTCCCAAGGGTGCCCCAAATCCTTGCGAATGGGATGAAGCTGCTACTGCTCTTGAA
ATAAACCTAGAAGAAGAGGACGATGACAACGAAGACGAAGTAGACGAGCAAGCTGAGCAGCAAAAAACTCACGTATTTG
GGCAGGCGCCTTATTCTGGTATAAATATTACAAAGGAGGGTATTCAAATAGGTGTCGAAGGTCAAACACCTAAATATGCCG
ATAAAACATTTCAACCTGAACCTCAAATAGGAGAATCTCAGTGGTACGAAACAGAAATTAATCATGCAGCTGGGAGAGTC

CTAAAAAAGACTACCCCAATGAAACCATGTTACGGTTCATATGCAAAACCCACAAATGAAAATGGAGGGCAAGGCATTCT
TGTAAAGCAACAAAATGGAAAGCTAGAAAGTCAAGTGGAAATGCAATTTTTCTCAACTACTGAGGCAGCCGCAGGCAATG
GTGATAACTTGACTCCTAAAGTGGTATTGTACAGTGAAGATGTAGATATAGAAACCCCAGACACTCATATTTCTTACATGCC
CACTATTAAGGAAGGTAACTCACGAGAACTAATGGGCCAACAATCTATGCCCAACAGGCCTAATTACATTGCTTTTAGGGA
CAATTTTATTGGTCTAATGTATTACAACAGCACGGGTAATATGGGTGTTCTGGCGGGCCAAGCATCGCAGTTGAATGCTGTT
GTAGATTTGCAAGACAGAAACACAGAGCTTTCATACCAGCTTTTGCTTGATTCCATTGGTGATAGAACCAGGTACTTTTCTA
TGTGGAATCAGGCTGTTGACAGCTATGATCCAGATGTTAGAATTATTGAAAATCATGGAACTGAAGATGAACTTCCAAATT
ACTGCTTTCCACTGGGAGGTGTGATTAATACAGAGACTCTTACCAAGGTAAAACCTAAAACAGGTCAGGAAAATGGATGG
GAAAAAGATGCTACAGAATTTTCAGATAAAAATGAAATAAGAGTTGGAAATAATTTTGCCATGGAAATCAATCTAAATGCC
AACCTGTGGAGAAATTTCCTGTACTCCAACATAGCGCTGTATTTGCCCGACAAGCTAAAGTACAGTCCTTCCAACGTAAAA
ATTTCTGATAACCCAAACACCTACGACTACATGAACAAGCGAGTGGTGGCTCCCGGGCTAGTGGACTGCTACATTAACCTT
GGAGCACGCTGGTCCCTTGACTATATGGACAACGTCAACCCATTTAACCACCACCGCAATGCTGGCCTGCGCTACCGCTC
AATGTTGCTGGGCAATGGTCGCTATGTGCCCTTCCACATCCAGGTGCCTCAGAAGTTCTTTGCCATTAAAAACCTCCTTCTC
CTGCCGGGCTCATACACCTACGAGTGGAACTTCAGGAAGGATGTTAACATGGTTCTGCAGAGCTCCCTAGGAAATGACCT
AAGGGTTGACGGAGCCAGCATTAAGTTTGATAGCATTTGCCTTTACGCCACCTTCTTCCCCATGGCCCACAACACCGCCTC
CACGCTTGAGGCCATGCTTAGAAACGACACCAACGACCAGTCCTTTAACGACTATCTCTCCGCCGCCAACATGCTCTACCC
TATACCCGCCAACGCTACCAACGTGCCCATATCCATCCCCTCCCGCAACTGGGCGGCTTTCCGCGGCTGGGCCTTCACGC
GCCTTAAGACTAAGGAAACCCCATCACTGGGCTCGGGCTACGACCCTTATTACACCTACTCTGGCTCTATACCCTACCTAG
ATGGAACCTTTTACCTCAACCACACCTTTAAGAAGGTGGCCATTACCTTTGACTCTTCTGTCAGCTGGCCTGGCAATGACCG
CCTGCTTACCCCCAACGAGTTTGAAATTAAGCGCTCAGTTGACGGGGAGGGTTACAACGTTGCCCAGTGTAACATGACCA
AAGACTGGTTCCTGGTACAAATGCTAGCTAACTATAACATTGGCTACCAGGGCTTCTATATCCCAGAGAGCTACAAGGACC
GCATGTACTCCTTCTTTAGAAACTTCCAGCCCATGAGCCGTCAGGTGGTGGATGATACTAAATACAAGGACTACCAACAGG
TGGGCATCCTACACCAACACAACAACTCTGGATTTGTTGGCTACCTTGCCCCCACCATGCGCGAAGGACAGGCCTACCCT
GCTAACTTCCCCTATCCGCTTATAGGCAAGACCGCAGTTGACAGCATTACCCAGAAAAAGTTTCTTTGCGATCGCACCCTTT
GGCGCATCCCATTCTCCAGTAACTTTATGTCCATGGGCGCACTCACAGACCTGGGCCAAAACCTTCTCTACGCCAACTCCG
CCCACGCGCTAGACATGACTTTTGAGGTGGATCCCATGGACGAGCCCACCCTTCTTTATGTTTTGTTTGAAGTCTTTGACGT
GGTCCGTGTGCACCAGCCGCACCGCGGCGTCATCGAAACCGTGTACCTGCGCACGCCCTTCTCGGCCGGCAACGCCACA
ACATAAAGAAGCAAGCAACATCAACAACAGCTGCCGCCATGGGCTCCAGTGAGCAGGAACTGAAAGCCATTGTCAAAGA
TCTTGGTTGTGGGCCATATTTTTTGGGCACCTATGACAAGCGCTTTCCAGGCTTTGTTTCTCCACACAAGCTCGCCTGCGCC
ATAGTCAATACGGCCGGTCGCGAGACTGGGGGCGTACACTGGATGGCCTTTGCCTGGAACCCGCACTCAAAAACATGCTA
CCTCTTTGAGCCCTTTGGCTTTTCTGACCAGCGACTCAAGCAGGTTTACCAGTTTGAGTACGAGTCACTCCTGCGCCGTAGC
GCCATTGCTTCTTCCCCCGACCGCTGTATAACGCTGGAAAAGTCCACCCAAAGCGTACAGGGGCCCAACTCGGCCGCCTG
TGGACTATTCTGCTGCATGTTTCTCCACGCCTTTGCCAACTGGCCCCAAACTCCCATGGATCACAACCCCACCATGAACCTT
ATTACCGGGGTACCCAACTCCATGCTCAACAGTCCCCAGGTACAGCCCACCCTGCGTCGCAACCAGGAACAGCTCTACAG
CTTCCTGGAGCGCCACTCGCCCTACTTCCGCAGCCACAGTGCGCAGATTAGGAGCGCCACTTCTTTTTGTCACTTGAAAAA
CATGTAAAAATAATGTACTAGAGACACTTTCAATAAAGGCAAATGCTTTTATTTGTACACTCTCGGGTGATTATTTACCCCCA
CCCTTGCCGTCTGCGCCGTTTAAAAATCAAAGGGGTTCTGCCGCGCATCGCTATGCGCCACTGGCAGGGACACGTTGCGA
TACTGGTGTTTAGTGCTCCACTTAAACTCAGGCACAACCATCCGCGGCAGCTCGGTGAAGTTTTCACTCCACAGGCTGCGC
ACCATCACCAACGCGTTTAGCAGGTCGGGCGCCGATATCTTGAAGTCGCAGTTGGGGCCTCCGCCCTGCGCGCGCGAGTT
GCGATACACAGGGTTGCAGCACTGGAACACTATCAGCGCCGGGTGGTGCACGCTGGCCAGCACGCTCTTGTCGGAGATC
AGATCCGCGTCCAGGTCCTCCGCGTTGCTCAGGGCGAACGGAGTCAACTTTGGTAGCTGCCTTCCCAAAAAGGGCGCGTG
CCCAGGCTTTGAGTTGCACTCGCACCGTAGTGGCATCAAAAGGTGACCGTGCCCGGTCTGGGCGTTAGGATACAGCGCCT
GCATAAAAGCCTTGATCTGCTTAAAAGCCACCTGAGCCTTTGCGCCTTCAGAGAAGAACATGCCGCAAGACTTGCCGGAA
AACTGATTGGCCGGACAGGCCGCGTCGTGCACGCAGCACCTTGCGTCGGTGTTGGAGATCTGCACCACATTTCGGCCCCA
CCGGTTCTTCACGATCTTGGCCTTGCTAGACTGCTCCTTCAGCGCGCGCTGCCCGTTTTCGCTCGTCACATCCATTTCAATC
ACGTGCTCCTTATTTATCATAATGCTTCCGTGTAGACACTTAAGCTCGCCTTCGATCTCAGCGCAGCGGTGCAGCCACAAC
GCGCAGCCCGTGGGCTCGTGATGCTTGTAGGTCACCTCTGCAAACGACTGCAGGTACGCCTGCAGGAATCGCCCCATCAT
CGTCACAAAGGTCTTGTTGCTGGTGAAGGTCAGCTGCAACCCGCGGTGCTCCTCGTTCAGCCAGGTCTTGCATACGGCCG
CCAGAGCTTCCACTTGGTCAGGCAGTAGTTTGAAGTTCGCCTTTAGATCGTTATCCACGTGGTACTTGTCCATCAGCGCGCG

CGCAGCCTCCATGCCCTTCTCCCACGCAGACACGATCGGCACACTCAGCGGGTTCATCACCGTAATTTCACTTTCCGCTTC
GCTGGGCTCTTCCTCTTCCTCTTGCGTCCGCATACCACGCGCCACTGGGTCGTCTTCATTCAGCCGCCGCACTGTGCGCTTA
CCTCCTTTGCCATGCTTGATTAGCACCGGTGGGTTGCTGAAACCCACCATTTGTAGCGCCACATCTTCTCTTTCTTCCTCGCT
GTCCACGATTACCTCTGGTGATGGCGGGCGCTCGGGCTTGGGAGAAGGGCGCTTCTTTTTCTTCTTGGGCGCAATGGCCAA
ATCCGCCGCCGAGGTCGATGGCCGCGGGCTGGGTGTGCGCGGCACCAGCGCGTCTTGTGATGAGTCTTCCTCGTCCTCGG
ACTCGATACGCCGCCTCATCCGCTTTTTTGGGGGCGCCCGGGGAGGCGGCGGCGACGGGGACGGGGACGACACGTCCTC
CATGGTTGGGGGACGTCGCGCCGCACCGCGTCCGCGCTCGGGGGTGGTTTCGCGCTGCTCCTCTTCCCGACTGGCCATTT
CCTTCTCCTATAGGCAGAAAAAGATCATGGAGTCAGTCGAGAAGAAGGACAGCCTAACCGCCCCCTCTGAGTTCGCCACC
ACCGCCTCCACCGATGCCGCCAACGCGCCTACCACCTTCCCCGTCGAGGCACCCCCGCTTGAGGAGGAGGAAGTGATTAT
CGAGCAGGACCCAGGTTTTGTAAGCGAAGACGACGAGGACCGCTCAGTACCAACAGAGGATAAAAAGCAAGACCAGGA
CAACGCAGAGGCAAACGAGGAACAAGTCGGGCGGGGGGACGAAAGGCATGGCGACTACCTAGATGTGGGAGACGACGT
GCTGTTGAAGCATCTGCAGCGCCAGTGCGCCATTATCTGCGACGCGTTGCAAGAGCGCAGCGATGTGCCCCTCGCCATAG
CGGATGTCAGCCTTGCCTACGAACGCCACCTATTCTCACCGCGCGTACCCCCCAAACGCCAAGAAAACGGCACATGCGAG
CCCAACCCGCGCCTCAACTTCTACCCCGTATTTGCCGTGCCAGAGGTGCTTGCCACCTATCACATCTTTTTCCAAAACTGCA
AGATACCCCTATCCTGCCGTGCCAACCGCAGCCGAGCGGACAAGCAGCTGGCCTTGCGGCAGGGCGCTGTCATACCTGAT
ATCGCCTCGCTCAACGAAGTGCCAAAAATCTTTGAGGGTCTTGGACGCGACGAGAAGCGCGCGGCAAACGCTCTGCAAC
AGGAAAACAGCGAAAATGAAAGTCACTCTGGAGTGTTGGTGGAACTCGAGGGTGACAACGCGCGCCTAGCCGTACTAAA
ACGCAGCATCGAGGTCACCCACTTTGCCTACCCGGCACTTAACCTACCCCCCAAGGTCATGAGCACAGTCATGAGTGAGC
TGATCGTGCGCCGTGCGCAGCCCCTGGAGAGGGATGCAAATTTGCAAGAACAAACAGAGGAGGGCCTACCCGCAGTTGG
CGACGAGCAGCTAGCGCGCTGGCTTCAAACGCGCGAGCCTGCCGACTTGGAGGAGCGACGCAAACTAATGATGGCCGCA
GTGCTCGTTACCGTGGAGCTTGAGTGCATGCAGCGGTTCTTTGCTGACCCGGAGATGCAGCGCAAGCTAGAGGAAACATT
GCACTACACCTTTCGACAGGGCTACGTACGCCAGGCCTGCAAGATCTCCAACGTGGAGCTCTGCAACCTGGTCTCCTACCT
TGGAATTTTGCACGAAAACCGCCTTGGGCAAAACGTGCTTCATTCCACGCTCAAGGGCGAGGCGCGCCGCGACTACGTCC
GCGACTGCGTTTACTTATTTCTATGCTACACCTGGCAGACGGCCATGGGCGTTTGGCAGCAGTGCTTGGAGGAGTGCAACC
TCAAGGAGCTGCAGAAACTGCTAAAGCAAAACTTGAAGGACCTATGGACGGCCTTCAACGAGCGCTCCGTGGCCGCGCA
CCTGGCGGACATCATTTTCCCCGAACGCCTGCTTAAAACCCTGCAACAGGGTCTGCCAGACTTCACCAGTCAAAGCATGTT
GCAGAACTTTAGGAACTTTATCCTAGAGCGCTCAGGAATCTTGCCCGCCACCTGCTGTGCACTTCCTAGCGACTTTGTGCC
CATTAAGTACCGCGAATGCCCTCCGCCGCTTTGGGGCCACTGCTACCTTCTGCAGCTAGCCAACTACCTTGCCTACCACTC
TGACATAATGGAAGACGTGAGCGGTGACGGTCTACTGGAGTGTCACTGTCGCTGCAACCTATGCACCCCGCACCGCTCCC
TGGTTTGCAATTCGCAGCTGCTTAACGAAAGTCAAATTATCGGTACCTTTGAGCTGCAGGGTCCCTCGCCTGACGAAAAGT
CCGCGGCTCCGGGGTTGAAACTCACTCCGGGGCTGTGGACGTCGGCTTACCTTCGCAAATTTGTACCTGAGGACTACCAC
GCCCACGAGATTAGGTTCTACGAAGACCAATCCCGCCCGCCTAATGCGGAGCTTACCGCCTGCGTCATTACCCAGGGCCA
CATTCTTGGCCAATTGCAAGCCATCAACAAAGCCCGCCAAGAGTTTCTGCTACGAAAGGGACGGGGGGTTTACTTGGACC
CCCAGTCCGGCGAGGAGCTCAACCCAATCCCCCCGCCGCCGCAGCCCTATCAGCAGCAGCCGCGGGCCCTTGCTTCCCA
GGATGGCACCCAAAAAGAAGCTGCAGCTGCCGCCGCCACCCACGGACGAGGAGGAATACTGGGACAGTCAGGCAGAGG
AGGTTTTGGACGAGGAGGAGGAGGACATGATGGAAGACTGGGAGAGCCTAGACGAGGAAGCTTCCGAGGTCGAAGAGG
TGTCAGACGAAACACCGTCACCCTCGGTCGCATTCCCCTCGCCGGCGCCCCAGAAATCGGCAACCGGTTCCAGCATGGCT
ACAACCTCCGCTCCTCAGGCGCCGCCGGCACTGCCCGTTCGCCGACCCAACCGTAGATGGGACACCACTGGAACCAGGG
CCGGTAAGTCCAAGCAGCCGCCGCCGTTAGCCCAAGAGCAACAACAGCGCCAAGGCTACCGCTCATGGCGCGGGCACA
AGAACGCCATAGTTGCTTGCTTGCAAGACTGTGGGGGCAACATCTCCTTCGCCCGCCGCTTTCTTCTCTACCATCACGGCG
TGGCCTTCCCCCGTAACATCCTGCATTACTACCGTCATCTCTACAGCCCATACTGCACCGGCGGCAGCGGCAGCAACAGC
AGCGGCCACACAGAAGCAAAGGCGACCGGATAGCAAGACTCTGACAAAGCCCAAGAAATCCACAGCGGCGGCAGCAGC
AGGAGGAGGAGCGCTGCGTCTGGCGCCCAACGAACCCGTATCGACCCGCGAGCTTAGAAACAGGATTTTTCCCACTCTGT
ATGCTATATTTCAACAGAGCAGGGGCCAAGAACAAGAGCTGAAAATAAAAAACAGGTCTCTGCGATCCCTCACCCGCAGC
TGCCTGTATCACAAAAGCGAAGATCAGCTTCGGCGCACGCTGGAAGACGCGGAGGCTCTCTTCAGTAAATACTGCGCGCT
GACTCTTAAGGACTAGTTTCGCGCCCTTTCTCAAATTTAAGCGCGAAAACTACGTCATCTCCAGCGGCCACACCCGGCGCC
AGCACCTGTTGTCAGCGCCATTATGAGCAAGGAAATTCCCACGCCCTACATGTGGAGTTACCAGCCACAAATGGGACTTG
CGGCTGGAGCTGCCCAAGACTACTCAACCCGAATAAACTACATGAGCGCGGACCCCACATGATATCCCGGGTCAACGG
AATACGCGCCCACCGAAACCGAATTCTCCTGGAACAGGCGGCTATTACCACCACACCTCGTAATAACCTTAATCCCCGTAG

TTGGCCCGCTGCCCTGGTGTACCAGGAAAGTCCCGCTCCCACCACTGTGGTACTTCCCAGAGACGCCCAGGCCGAAGTTC
AGATGACTAACTCAGGGGCGCAGCTTGCGGGCGGCTTTCGTCACAGGGTGCGGTCGCCCGGGCAGGGTATAACTCACCT
GACAATCAGAGGGCGAGGTATTCAGCTCAACGACGAGTCGGTGAGCTCCTCGCTTGGTCTCCGTCCGGACGGGACATTTC
AGATCGGCGGCGCCGGCCGCTCTTCATTCACGCCTCGTCAGGCAATCCTAACTCTGCAGACCTCGTCCTCTGAGCCGCGCT
CTGGAGGCATTGGAACTCTGCAATTTATTGAGGAGTTTGTGCCATCGGTCTACTTTAACCCCTTCTCGGGACCTCCCGGCCA
CTATCCGGATCAATTTATTCCTAACTTTGACGCGGTAAAGGACTCGGCGGACGGCTACGACTGAATGTTAAGTGGAGAGGC
AGAGCAACTGCGCCTGAAACACCTGGTCCACTGTCGCCGCCACAAGTGCTTTGCCCGCGACTCCGGTGAGTTTTGCTACTT
TGAATTGCCCGAGGATCATATCGAGGGCCCGGCGCACGGCGTCCGGCTTACCGCCCAGGGAGAGCTTGCCCGTAGCCTG
ATTCGGGAGTTTACCCAGCGCCCCCTGCTAGTTGAGCGGGACAGGGGACCCTGTGTTCTCACTGTGATTTGCAACTGTCCT
AACCCTGGATTACATCAAGATCTTTGTTGCCATCTCTGTGCTGAGTATAATAAATACAGAAATTAAAATATACTGGGGCTCC
TATCGCCATCCTGTAAACGCCACCGTCTTCACCCGCCCAAGCAAACCAAGGCGAACCTTACCTGGTACTTTTAACATCTCT
CCCTCTGTGATTTACAACAGTTTCAACCCAGACGGAGTGAGTCTACGAGAGAACCTCTCCGAGCTCAGCTACTCCATCAGA
AAAAACACCACCCTCCTTACCTGCCGGGAACGTACGAGTGCGTCACCGGCCGCTGCACCACACCTACCGCCTGACCGTAA
ACCAGACTTTTTCCGGACAGACCTCAATAACTCTGTTTACCAGAACAGGAGGTGAGCTTAGAAAACCCTTAGGGTATTAGG
CCAAAGGCGCAGCTACTGTGGGGTTTATGAACAATTCAAGCAACTCTACGGGCTATTCTAATTCAGGTTTCTCTAGAAATG
GACGGAATTATTACAGAGCAGCGCCTGCTAGAAAGACGCAGGGCAGCGGCCGAGCAACAGCGCATGAATCAAGAGCTCC
AAGACATGGTTAACTTGCACCAGTGCAAAAGGGGTATCTTTTGTCTGGTAAAGCAGGCCAAAGTCACCTACGACAGTAATA
CCACCGGACACCGCCTTAGCTACAAGTTGCCAACCAAGCGTCAGAAATTGGTGGTCATGGTGGGAGAAAAGCCCATTACC
ATAACTCAGCACTCGGTAGAAACCGAAGGCTGCATTCACTCACCTTGTCAAGGACCTGAGGATCTCTGCACCCTTATTAAG
ACCCTGTGCGGTCTCAAAGATCTTATTCCCTTTAACTAATAAAAAAAAATAATAAAGCATCACTTACTTAAAATCAGTTAGC
AAATTTCTGTCCAGTTTATTCAGCAGCACCTCCTTGCCCTCCTCCCAGCTCTGGTATTGCAGCTTCCTCCTGGCTGCAAACTT
TCTCCACAATCTAAATGGAATGTCAGTTTCCTCCTGTTCCTGTCCATCCGCACCCACTATCTTCATGTTGTTGCAGATGAAGC
GCGCAAGACCGTCTGAAGATACCTTCAACCCCGTGTATCCATATGACACGGAAACCGGTCCTCCAACTGTGCCTTTTCTTA
CTCCTCCCTTTGTATCCCCCAATGGGTTTCAAGAGAGTCCCCCTGGGGTACTCTCTTTGCGCCTATCCGAACCTCTAGTTAC
CTCCAATGGCATGCTTGCGCTCAAAATGGGCAACGGCCTCTCTCTGGACGAGGCCGGCAACCTTACCTCCCAAAATGTAA
CCACTGTGAGCCCACCTCTCAAAAAAACCAAGTCAAACATAAACCTGGAAATATCTGCACCCCTCACAGTTACCTCAGAA
GCCCTAACTGTGGCTGCCGCCGCACCTCTAATGGTCGCGGGCAACACACTCACCATGCAATCACAGGCCCCGCTAACCGT
GCACGACTCCAAACTTAGCATTGCCACCCAAGGACCCCTCACAGTGTCAGAAGGAAAGCTAGCCCTGCAAACATCAGGCC
CCCTCACCACCACCGATAGCAGTACCCTTACTATCACTGCCTCACCCCCTCTAACTACTGCCACTGGTAGCTTGGGCATTG
ACTTGAAAGAGCCCATTTATACACAAAATGGAAAACTAGGACTAAAGTACGGGGCTCCTTTGCATGTAACAGACGACCTA
AACACTTTGACCGTAGCAACTGGTCCAGGTGTGACTATTAATAATACTTCCTTGCAAACTAAAGTTACTGGAGCCTTGGGTT
TTGATTCACAAGGCAATATGCAACTTAATGTAGCAGGAGGACTAAGGATTGATTCTCAAAACAGACGCCTTATACTTGATG
TTAGTTATCCGTTTGATGCTCAAAACCAACTAAATCTAAGACTAGGACAGGGCCCTCTTTTTATAAACTCAGCCCACAACTT
GGATATTAACTACAACAAAGGCCTTTACTTGTTTACAGCTTCAAACAATTCCAAAAAGCTTGAGGTTAACCTAAGCACTGCC
AAGGGGTTGATGTTTGACGCTACAGCCATAGCCATTAATGCAGGAGATGGGCTTGAATTTGGTTCACCTAATGCACCAAAC
ACAAATCCCCTCAAAACAAAAATTGGCCATGGCCTAGAATTTGATTCAAACAAGGCTATGGTTCCTAAACTAGGAACTGGC
CTTAGTTTTGACAGCACAGGTGCCATTACAGTAGGAAACAAAAATAATGATAAGCTAACTTTGTGGACCACACCAGCTCCA
TCTCCTAACTGTAGACTAAATGCAGAGAAAGATGCTAAACTCACTTTGGTCTTAACAAAATGTGGCAGTCAAATACTTGCTA
CAGTTTCAGTTTTGGCTGTTAAAGGCAGTTTGGCTCCAATATCTGGAACAGTTCAAAGTGCTCATCTTATTATAAGATTTGAC
GAAAATGGAGTGCTACTAAACAATTCCTTCCTGGACCCAGAATATTGGAACTTTAGAAATGGAGATCTTACTGAAGGCACA
GCCTATACAAACGCTGTTGGATTTATGCCTAACCTATCAGCTTATCCAAAATCTCACGGTAAAACTGCCAAAAGTAACATTG
TCAGTCAAGTTTACTTAAACGGAGACAAAACTAAACCTGTAACACTAACCATTACACTAAACGGTACACAGGAAACAGGA
GACACAACTCCAAGTGCATACTCTATGTCATTTTCATGGGACTGGTCTGGCCACAACTACATTAATGAAATATTTGCCACAT
CCTCTTACACTTTTTCATACATTGCCCAAGAATAAAGAATCGTTTGTGTTATGTTTCAACGTGTTTATTTTTCAATTGCAGAAA
ATTTCGAATCATTTTTCATTCAGTAGTATAGCCCCACCACCACATAGCTTATACAGATCACCGTACCTTAATCAAACTCACA
GAACCCTAGTATTCAACCTGCCACCTCCCTCCCAACACACAGAGTACACAGTCCTTTCTCCCCGGCTGGCCTTAAAAAGCA
TCATATCATGGGTAACAGACATATTCTTAGGTGTTATATTCCACACGGTTTCCTGTCGAGCCAAACGCTCATCAGTGATATT
AATAAACTCCCCGGGCAGCTCACTTAAGTTCATGTCGCTGTCCAGCTGCTGAGCCACAGGCTGCTGTCCAACTTGCGGTTG
CTTAACGGGCGGCGAAGGAGAAGTCCACGCCTACATGGGGGTAGAGTCATAATCGTGCATCAGGATAGGGCGGTGGTGC

TGCAGCAGCGCGAATAAACTGCTGCCGCCGCCGCTCCGTCCTGCAGGAATACAACATGGCAGTGGTCTCCTCAGCGAT
GATTCGCACCGCCCGCAGCATAAGGCGCCTTGTCCTCCGGGCACAGCAGCGCACCCTGATCTCACTTAAATCAGCACAGT
AACTGCAGCACAGCACCACAATATTGTTCAAAATCCCACAGTGCAAGGCGCTGTATCCAAAGCTCATGGCGGGGACCACA
GAACCCACGTGGCCATCATACCACAAGCGCAGGTAGATTAAGTGGCGACCCCTCATAAACACGCTGGACATAAACATTAC
CTCTTTTGGCATGTTGTAATTCACCACCTCCCGGTACCATATAAACCTCTGATTAAACATGGCGCCATCCACCACCATCCTA
AACCAGCTGGCCAAAACCTGCCCGCCGGCTATACACTGCAGGGAACCGGGACTGGAACAATGACAGTGGAGAGCCCAG
GACTCGTAACCATGGATCATCATGCTCGTCATGATATCAATGTTGGCACAACACAGGCACACGTGCATACACTTCCTCAGG
ATTACAAGCTCCTCCCGCGTTAGAACCATATCCCAGGGAACAACCCATTCCTGAATCAGCGTAAATCCCACACTGCAGGG
AAGACCTCGCACGTAACTCACGTTGTGCATTGTCAAAGTGTTACATTCGGGCAGCAGCGGATGATCCTCCAGTATGGTAGC
GCGGGTTTCTGTCTCAAAAGGAGGTAGACGATCCCTACTGTACGGAGTGCGCCGAGACAACCGAGATCGTGTTGGTCGTA
GTGTCATGCCAAATGGAACGCCGGACGTAGTCATATTTCCTGAAGCAAAACCAGGTGCGGGCGTGACAAACAGATCTGCG
TCTCCGGTCTCGCCGCTTAGATCGCTCTGTGTAGTAGTTGTAGTATATCCACTCTCTCAAAGCATCCAGGCGCCCCCTGGCT
TCGGGTTCTATGTAAACTCCTTCATGCGCCGCTGCCCTGATAACATCCACCACCGCAGAATAAGCCACACCCAGCCAACCT
ACACATTCGTTCTGCGAGTCACACACGGGAGGAGCGGGAAGAGCTGGAAGAACCATGTTTTTTTTTTTATTCCAAAAGATT
ATCCAAAACCTCAAAATGAAGATCTATTAAGTGAACGCGCTCCCCTCCGGTGGCGTGGTCAAACTCTACAGCCAAAGAAC
AGATAATGGCATTTGTAAGATGTTGCACAATGGCTTCCAAAAGGCAAACGGCCCTCACGTCCAAGTGGACGTAAAGGCTA
AACCCTTCAGGGTGAATCTCCTCTATAAACATTCCAGCACCTTCAACCATGCCCAAATAATTCTCATCTCGCCACCTTCTCA
ATATATCTCTAAGCAAATCCCGAATATTAAGTCCGGCCATTGTAAAAATCTGCTCCAGAGCGCCCTCCACCTTCAGCCTCAA
GCAGCGAATCATGATTGCAAAAATTCAGGTTCCTCACAGACCTGTATAAGATTCAAAAGCGGAACATTAACAAAAATACCG
CGATCCCGTAGGTCCCTTCGCAGGGCCAGCTGAACATAATCGTGCAGGTCTGCACGGACCAGCGCGGCCACTTCCCCGCC
AGGAACCATGACAAAAGAACCCACACTGATTATGACACGCATACTCGGAGCTATGCTAACCAGCGTAGCCCCGATGTAAG
CTTGTTGCATGGGCGGCGATATAAAATGCAAGGTGCTGCTCAAAAAATCAGGCAAAGCCTCGCGCAAAAAAGAAAGCAC
ATCGTAGTCATGCTCATGCAGATAAAGGCAGGTAAGCTCCGGAACCACCACAGAAAAAGACACCATTTTTCTCTCAAACAT
GTCTGCGGGTTTCTGCATAAACACAAAATAAAATAACAAAAAAACATTTAAACATTAGAAGCCTGTCTTACAACAGGAAAA
ACAACCCTTATAAGCATAAGACGGACTACGGCCATGCCGGCGTGACCGTAAAAAAACTGGTCACCGTGATTAAAAAGCAC
CACCGACAGCTCCTCGGTCATGTCCGGAGTCATAATGTAAGACTCGGTAAACACATCAGGTTGATTCACATCGGTCAGTGC
TAAAAAGCGACCGAAATAGCCCGGGGGAATACATACCCGCAGGCGTAGAGACAACATTACAGCCCCCATAGGAGGTATA
ACAAAATTAATAGGAGAGAAAAACACATAAACACCTGAAAAACCCTCCTGCCTAGGCAAAATAGCACCCTCCCGCTCCAG
AACAACATACAGCGCTTCCACAGCGGCAGCCATAACAGTCAGCCTTACCAGTAAAAAAGAAAACCTATTAAAAAAACACC
ACTCGACACGGCACCAGCTCAATCAGTCACAGTGTAAAAAAGGGCCAAGTGCAGAGCGAGTATATATAGGACTAAAAAAT
GACGTAACGGTTAAAGTCCACAAAAAACACCCAGAAAACCGCACGCGAACCTACGCCCAGAAACGAAAGCCAAAAAACC
CACAACTTCCTCAAATCGTCACTTCCGTTTTCCCACGTTACGTCACTTCCCATTTTAAGAAAACTACAATTCCCAACACATAC
AAGTTACTCCGCCCTAAAACCTACGTCACCCGCCCCGTTCCCACGCCCCGCGCCACGTCACAAACTCCACCCCCTCATTAT
CATATTGGCTTCAATCCAAAATAAGGTATATTATTGATGATGTTAATTAATTTAAATCCGCATGCGATATCGAGCTCTCCCGG
GAATTCGGATCTGCGACGCGAGGCTGGATGGCCTTCCCCATTATGATTCTTCTCGCTTCCGGCGGCATCGGGATGCCCGCG
TTGCAGGCCATGCTGTCCAGGCAGGTAGATGACGACCATCAGGGACAGCTTCAAGGCCAGCAAAAGGCCAGGAACCGTA
AAAAGGCCGCGTTGCTGGCGTTTTTCCATAGGCTCCGCCCCCCTGACGAGCATCACAAAAATCGACGCTCAAGTCAGAGG
TGGCGAAACCCGACAGGACTATAAAGATACCAGGCGTTTCCCCCTGGAAGCTCCCTCGTGCGCTCTCCTGTTCCGACCCTG
CCGCTTACCGGATACCTGTCCGCCTTTCTCCCTTCGGGAAGCGTGGCGCTTTCTCATAGCTCACGCTGTAGGTATCTCAGTT
CGGTGTAGGTCGTTCGCTCCAAGCTGGGCTGTGTGCACGAACCCCCCGTTCAGCCCGACCGCTGCGCCTTATCCGGTAAC
TATCGTCTTGAGTCCAACCCGGTAAGACACGACTTATCGCCACTGGCAGCAGCCACTGGTAACAGGATTAGCAGAGCGAG
GTATGTAGGCGGTGCTACAGAGTTCTTGAAGTGGTGGCCTAACTACGGCTACACTAGAAGGACAGTATTTGGTATCTGCGC
TCTGCTGAAGCCAGTTACCTTCGGAAAAAGAGTTGGTAGCTCTTGATCCGGCAAACAAACCACCGCTGGTAGCGGTGGTTT
TTTTGTTTGCAAGCAGCAGATTACGCGCAGAAAAAAAGGATCTCAAGAAGATCCTTTGATCTTTTCTACGGGGTCTGACGCT
CAGTGGAACGAAAACTCACGTTAAGGGATTTTGGTCATGAGATTATCAAAAAGGATCTTCACCTAGATCCTTTTAAATCAAT
CTAAAGTATATATGAGTAAACTTGGTCTGACAGTTACCAATGCTTAATCAGTGAGGCACCTATCTCAGCGATCTGTCTATTT
CGTTCATCCATAGTTGCCTGACTCCCCGTCGTGTAGATAACTACGATACGGGAGGGCTTACCATCTGGCCCCAGTGCTGCA
ATGATACCGCGAGACCCACGCTCACCGGCTCCAGATTTATCAGCAATAAACCAGCCAGCCGGAAGGGCCGAGCGCAGAA
GTGGTCCTGCAACTTTATCCGCCTCCATCCAGTCTATTAATTGTTGCCGGGAAGCTAGAGTAAGTAGTTCGCCAGTTAATAG

TTTGCGCAACGTTGTTGCCATTGCTACAGGCATCGTGGTGTCACGCTCGTCGTTTGGTATGGCTTCATTCAGCTCCGGTTCC
CAACGATCAAGGCGAGTTACATGATCCCCCATGTTGTGCAAAAAAGCGGTTAGCTCCTTCGGTCCTCCGATCGTTGTCAGA
AGTAAGTTGGCCGCAGTGTTATCACTCATGGTTATGGCAGCACTGCATAATTCTCTTACTGTCATGCCATCCGTAAGATGCT
TTTCTGTGACTGGTGAGTACTCAACCAAGTCATTCTGAGAATAGTGTATGCGGCGACCGAGTTGCTCTTGCCCGGCGTCAA
CACGGGATAATACCGCGCCACATAGCAGAACTTTAAAAGTGCTCATCATTGGAAAACGTTCTTCGGGGCGAAAACTCTCA
AGGATCTTACCGCTGTTGAGATCCAGTTCGATGTAACCCACTCGTGCACCCAACTGATCTTCAGCATCTTTTACTTTCACCA
GCGTTTCTGGGTGAGCAAAAACAGGAAGGCAAAATGCCGCAAAAAAGGGAATAAGGGCGACACGGAAATGTTGAATACT
CATACTCTTCCTTTTTCAATATTATTGAAGCATTTATCAGGGTTATTGTCTCATGAGCGGATACATATTTGAATGTATTTAGAA
AAATAAACAAATAGGGGTTCCGCGCACATTTCCCCGAAAAGTGCCACCTGACGTCTAAGAAACCATTATTATCATGACATT
AACCTATAAAAATAGGCGTATCACGAGGCCCTTTCGTCTTCAAGAATTGGATCCGAATTCCCGGGAGAGCTCGATATCGCA
TGCGGATTTAAATTAATTAA
