# Supplementary figures and images for: Immune Response Elicited by Recombinant Adenovirus-Delivered Glycoprotein B and Nucleocapsid Protein UL18 and UL25 of HSV-1 in Mice
Source: Int J Mol Sci. 2024 Dec 16;25(24):13486. doi: 10.3390/ijms252413486 (PMC11678876; doi:10.3390/ijms252413486)

Figure 1C

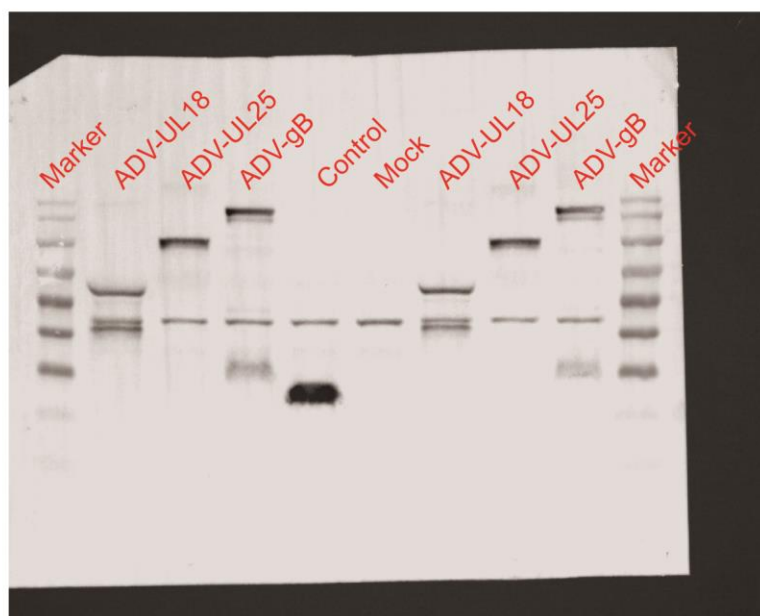

Supplement: Supplementary file 1 [file ijms-25-13486-s001.zip › Supplementary Files/Original WB Figure 1C.pdf]
